# Investigation of Novel Quinoline–Thiazole Derivatives as Antimicrobial Agents: *In Vitro* and *In Silico* Approaches

**DOI:** 10.1021/acsomega.2c06871

**Published:** 2022-12-29

**Authors:** Asaf Evrim Evren, Abdullah Burak Karaduman, Begüm Nurpelin Sağlik, Yusuf Özkay, Leyla Yurttaş

**Affiliations:** † Department of Pharmacy Services, Vocational School of Health Services, Bilecik Şeyh Edebali University, Bilecik 11000, Turkey; ‡ Department of Pharmaceutical Chemistry, Faculty of Pharmacy, Anadolu University, Eskişehir 26470, Turkey; § Department of Pharmaceutical Toxicology, Faculty of Pharmacy, Anadolu University, Eskişehir 26470, Turkey; ∥ Central Research Laboratory, Faculty of Pharmacy, Anadolu University, Eskişehir 26470, Turkey

## Abstract

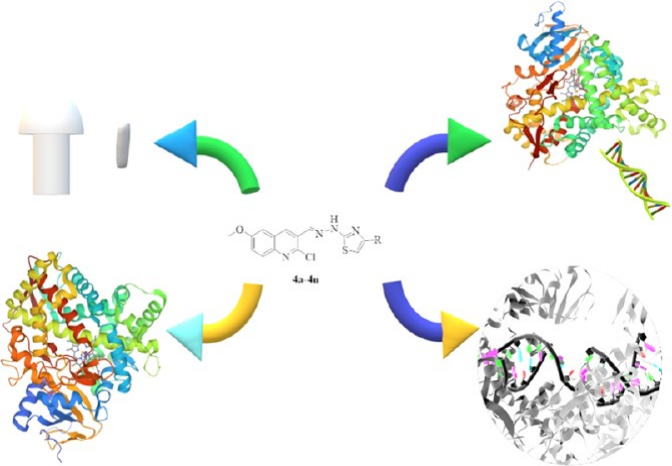

Infectious diseases are a major concern around the world. Today, it is an urgent need for new chemotherapeutics for infectious diseases. Because of that, our group designed, synthesized, and analyzed 14 new quinoline derivatives endowed with the pharmacophore moiety of fluoroquinolones primarily for their antimicrobial effects. Their cytotoxicity effects were tested against six bacterial and four fungal strains and NIH/3T3 cell line. Additionally, their action mechanisms were evaluated against DNA gyrase and lanosterol 14α-demethylase (LMD). Furthermore, to eliminate the potential side effects, the active compounds were evaluated against the aromatase enzyme. The experimental enzymatic results were evaluated for active compounds’ binding modes using molecular docking and molecular dynamics simulation studies. The results were utilized to clarify the structure–activity relationship (SAR). Finally, compound **4m** was the most potent compound for its antifungal activity with low cytotoxicity against healthy cells and fewer possible side effects, while compounds **4j** and **4l** can be used alone for special patients who are suffering from fungal infections in addition to the primer disease.

## 1 Introduction

Infection is described as pathogenic microorganisms’ invasion and/or multiplying in the tissue or organ of another organism and then causing some undesirable effects in the host.^1,2^ There are many pathogenic microorganisms like viruses, bacteria, fungi, yeast, *etc.* However, the incidence of bacterial and fungal infections is quite high worldwide than others.^3^

Although humanity has a symbiotic relationship with nonpathogenic bacteria and fungi, this relationship can be disrupted, and at this time, invasive microorganisms that can take advantage of this situation may cause infection.^4−7^ They can also be contagious; various factors such as water, food, and travel play a role in the spread of these kinds of diseases.^8^ In recent decades, resistance development is a major issue and is classified as drug-induced and microorganism-induced resistances. Mostly, they are related to unnecessary (incorrect dose or use of drugs in unrelated indications)^9^ and multiple drug uses.^10−12^ Additionally, misdiagnosis and wrong treatment methods,^13^ prescriptions written only to eliminate symptoms (symptomatic treatment), or patients abandoning their treatment unfinished^14^ make also some difficulties in the fighting against infectious diseases. Besides that, microbial infections are frequently seen in patients whose immune system is suppressed or insufficient, such as cancer and AIDS patients. In recent studies, the reported mortality and morbidity rates of microbial resistance developed by bacteria and fungi increased the concerns,^15,16^ especially regarding the inefficacy of the treatment applied in hospitals.^17^

Today, although the antimicrobial agents are classified depending on different properties such as action mechanism, spectrum width, and chemical structure, the structure–activity relationship (SAR) comes to the fore for medicinal chemists.^18^ With the clinical use of nalidixic acid in 1964, scientists focused on the antimicrobial activity of quinoline derivatives.^19^ Quinoline derivatives also play a role in cancer treatments (topoisomerase inhibition) due to their action mechanism (DNA-gyrase inhibition).^20−22^ The structures of bacterial and eukaryotic topoisomerase IIs were identified, and they are highly similar to each other.^23^ Even though this link seems satisfying, and the result may perceive as dual activity, they should be tested against healthy human cells in the current preclinical phase.

In addition, quinolones have a good antifungal activity profile^24−26^ besides their antibacterial activity. Currently, the action mechanism of the drugs for the treatment of fungal diseases is generally based on blocking the ergosterol production because of their selectivity profile,^27,28^ and with that, they break the fungal cell integrity. The spectacular pathway for this activity is the inhibition of lanosterol 14α-demethylase (LDM).^29^ Indeed, each antifungal imidazole analogue has previously been studied on P450, and they have been identified as potent ligands of the heme iron atom of P450s.^30^ Moreover, ravuconazole and its analogues (fosravuconazole and isavuconazole) include a thiazole ring as a secondary azole, and albaconazole includes quinazolin-4-one ring, and their action mechanism is mediated through P450 enzymes.^31,32^ Hence, it was proven that the other azole rings [such as (benzo)-1,2,3-triazole,^33,34^ 1,2,4-triazole,^35,36^ 1,3,4-oxadiazole,^37^ and 1,3-thiazole] and non-azole nitrogen-rich rings may have also a good antifungal activity profile against invasive fungi. However, several side effects were reported,^38,39^ probably caused by similarity to the human aromatase (HA) enzyme,^40^ and also they showed some drug–drug interactions.^41^ Hence, the design strategy should also involve inhibiting the LDM while not inhibiting HA.

**Figure 1:**
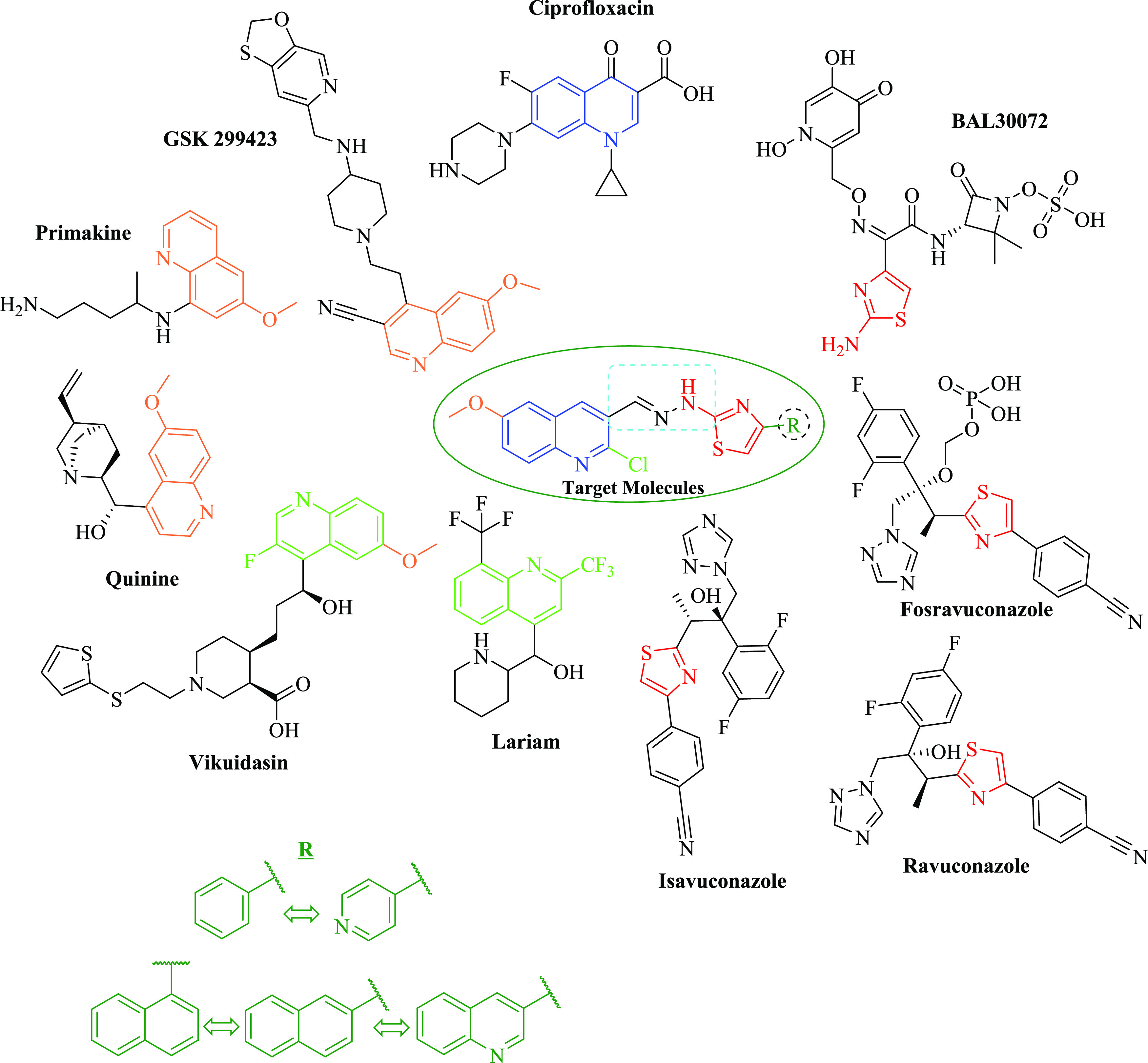
Designing the strategy of new quinoline derivatives as an antimicrobial agent. At the center of the figure, the target compounds are presented. Turquoise drawings schematize the analogue ring systems. Each color represents a moiety of target compounds.

Based on the above information, we designed, synthesized, and analyzed 14 new quinoline derivatives endowed with the pharmacophore moiety of fluoroquinolones to observe the antimicrobial effect. For this purpose, the literature knowledge was used to achieve a hybridization of the pharmacophoric groups of the molecules, as seen in Figure 1. The final molecules include 6-methoxy-2-chloroquinoline as the core structure. Their derivatization was achieved at the fourth position of the thiazole ring, and both aromatic ring systems were linked to each other with the hydrazone moiety. Their structure–activity relationship (SAR) and action mechanism were clarified *viain vitro* and *in silico* studies.

## 2 Results and Discussion

### 2.1 Chemistry

The designed compounds (see Table 1) were synthesized and analyzed. The analyzed spectra are provided in the Supporting Information (Figures S1–S42).

**Table 1:**
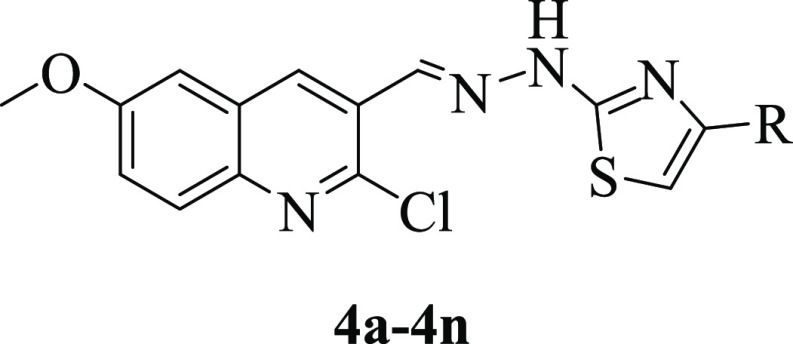

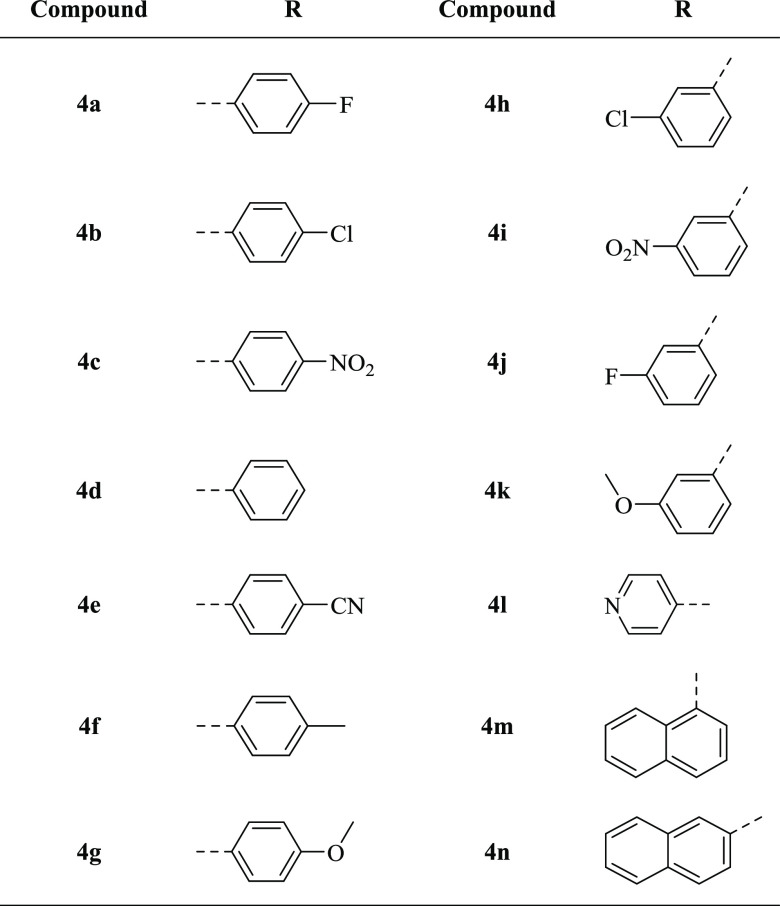
Synthesized Compounds

#### 2.1.1 ^1^H NMR

Thiazole and quinoline are the common ring systems in all final compounds. In the sixth position of the quinoline, there is a methoxy and in the second position there is a chlorine substituent. Different aromatic substitutions were made at the fourth position of the thiazole ring, and the quinoline and the thiazole ring were linked by a hydrazone bridge. By investigating ^1^H NMR spectra, the peak due to the fourth position proton of the quinoline was observed as a singlet between 8.68 and 8.78 ppm. The peak of hydrogen in the eighth position is observed as a doublet in the range of 7.82–7.91 ppm, and it is observed as a multiplet in some spectra because it is overlapped with other peaks. The peak due to the fourth position of the quinoline proton was observed as a doublet in the range of 7.58–7.68 ppm because of the surrounding proton interactions. The peak due to the seventh position of the quinoline was observed in the 7.42–7.52 ppm range as doublets due to the surrounding proton interactions, and as multiplet, because it was partially overlapped with other peaks. The proton peak at the fifth position of the thiazole ring was observed generally as a singlet between 7.21 and 7.81 ppm, and multiplet was obtained since it was partially overlapped with other proton peaks. The two hydrogens, at the second and sixth positions of the 1,4-substituted phenyl derivatives (**4a**–**c** and **4e**–**g**), were observed as doublets between 7.75 and 8.27 ppm, while the other two protons of the third and fifth positions were observed overlapped and as doublets between 6.97 and 8.11 ppm. Hydrogens of 3-substituted phenyl derivatives (**4h**–**k**) were obtained in the range of 6.87–8.65 ppm. According to the literature, the protons of the 4-substituted phenyl ring, 3-substituted phenyl ring, and mono-substituted phenyl ring follow the X_2_Y_2_, XYZW, and X_2_Y_2_Z spin systems, respectively. Although the cleavage patterns of the protons of the 1,4-disubstituted phenyl ring are mostly observed as two doublets (one for positions 2 and 6, one for positions 3 and 5), they can also be observed as singlet and quartet due to the properties of the substitutions.^42−44^ Although proton cleavages of the 1,3-disubstituted phenyl ring are generally observed as one triplet (fifth position), two doublets (fourth and sixth positions), and one singlet (second position), very different cleavages can be observed due to the substitutions.^43,45^

The protons of the methylidene hydrazine (−HC=N–NH−) structure, which is common in all compounds, are generally singlet in the range of 8.41–8.49 ppm and partially multiplet due to mixing with other peaks; nitrogen proton was obtained as a broad singlet between 12.56 and 12.73 ppm. In the literature, they were observed as singlet and broad singlet.^43,46^ When similar molecules were examined in the literature,^47^ it was determined experimentally that compounds with a singlet peak at about 8.50 ppm were *E* isomers. This finding is suggested in this direction in terms of stability due to the presence of bulky groups in the structure of the compound.

In conclusion, the ^1^H NMR data of all synthesized compounds were found to be consistent with the literature data.

#### 2.1.2 ^13^C NMR

According to the ^13^C NMR data of the synthesized compounds, peaks were observed as expected. The common carbons’ peaks and the unique carbons’ peaks of the compounds were examined. Since compounds **4a** and **4j** contain fluorine as a substituent, the spectra of these compounds were obtained as one additional peak, as mentioned in the literature due to C–F cleavages.^48^ The peaks of the carbon due to methylidene hydrazine, which is a common carbon for all compounds, were obtained in the range of 143.23–143.82 ppm similar to the literature.^46^ Aromatic carbons peaked in the range of 102.64–168.62 ppm, and methoxy carbons were seen between 55.55 and 56.24 ppm.

#### 2.1.3 High-Resolution Mass Spectrum

Mass spectra of the final compounds were obtained using the electrospray ionization (ESI) technique.^43^ When the mass spectra were examined, M + 1 peaks were detected in all compounds (**4a**–**n**).

### 2.2 Results of Antibacterial Tests

Interestingly, no antibacterial effect was observed in compounds other than **4g**, **4m**, and **4n**. However, when the antibacterial activities of these three compounds, especially **4g** and **4m**, were compared with the antibacterial effects of ciprofloxacin and chloramphenicol, they have a good antibacterial profile. Results of the antibacterial activity are shown in Table 2.

**Table 2:**
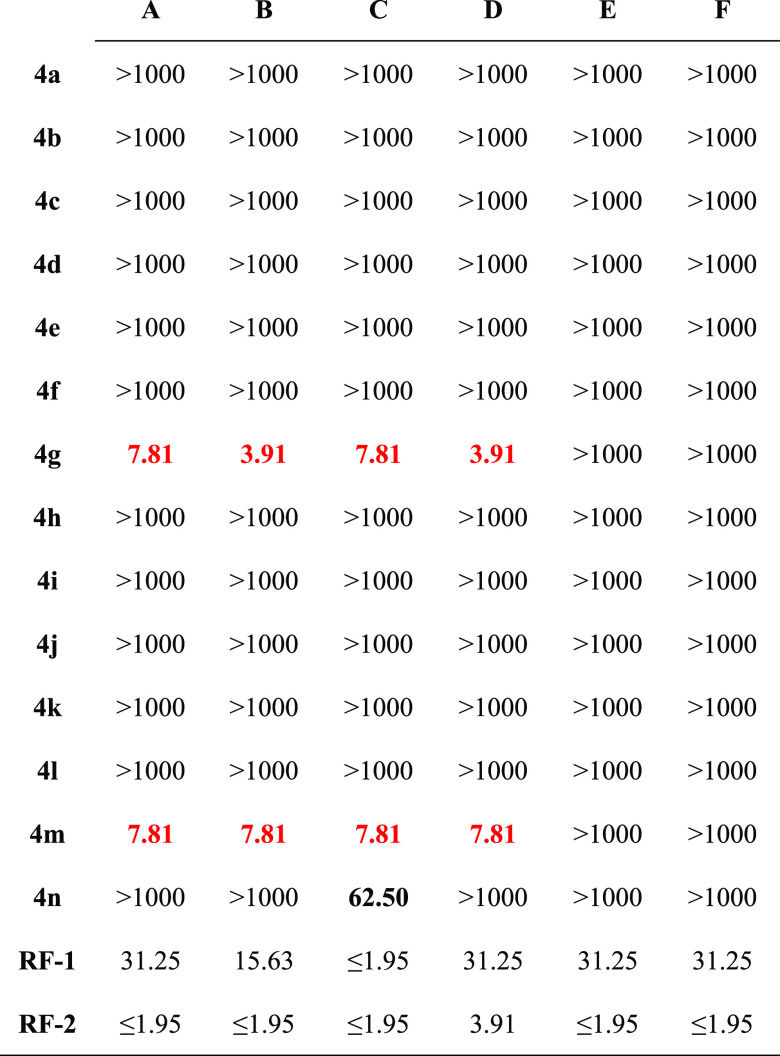
Antibacterial Activity Results of the Final Compounds (μg/mL)^a^

a RF-1: chloramphenicol, RF-2: ciprofloxacin, A: *Escherichia coli* ATCC 35218, B: *E. coli* ATCC 25922, C: *Staphylococcus aureus* ATCC 6538, D: methicillin-resistant *S. aureus* (MRSA) (clinical isolate), E: *Salmonella typhimurium* ATCC 13311, F: *Klebsiella pneumoniae* NCTC 9633. Most active compounds are highlighted in red and moderately active compounds are written in bold.

Minimum inhibitory concentrations (MIC_90_) of chloramphenicol, which inhibit 90% of the microbial population, were determined as 31.25 and 15.63 μg/mL against *E. coli* (ATCC 35218) and *E. coli* (ATCC 25922) strains, respectively. On the other hand, the MIC_90_ of ciprofloxacin was found to be lower than 1.95 μg/mL. Therefore, it was concluded that compound **4g** (MIC_90_: 7.81; 3.91 μg/mL) was four times more effective against both *E. coli* strains (ATCC 35218; ATCC 25922) than chloramphenicol. Like **4g**, compound **4m** (MIC_90_: 7.81; 7.81 μg/mL) was four times more effective against *E. coli* (ATCC 35218) and twice as effective against *E. coli* (ATCC 25922). The MIC_90_ values of chloramphenicol against *S. aureus* (ATCC 6538) and methicillin-resistant *S. aureus* (MRSA) (clinical isolate) strains were ≤1.95 and 31.25 μg/mL, respectively. According to these findings, compound **4g** (MIC_90_: 3.91 μg/mL) was 8 times more effective than the standard drug against methicillin-resistant *S. aureus* (MRSA) (clinical isolate), while compound **4m** (MIC_90_: 7.81 μg/mL) was 4 times more effective.

Compounds **4g**, **4m**, and **4n** showed significant antibacterial activity against *S. aureus* (ATCC 6538) strain at 7.81, 7.81, and 62.50 μg/mL, respectively.

The synthesized compounds (**4a**–**n**) did not show antibacterial activity against *S. typhimurium* (ATCC 13311) and *K. pneumoniae* (NCTC 9633) strains.

### 2.3 Results of Antifungal Tests

Ketoconazole was used as the reference drug to evaluate the anticandidal activities of the final compounds. Its MIC_90_ values were determined as 0.24 μg/mL against *Candida glabrata* (ATCC 90030) and less than 0.06 μg/mL for other species. Results of the antifungal activity are shown in Table 3.

**Table 3:**
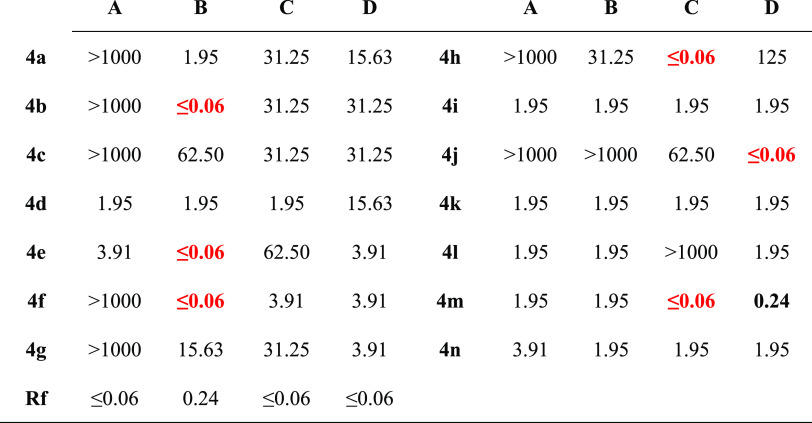
Anticandidal Activity Results of the Final Compounds (μg/mL)^a^

a RF: ketoconazole, A: *Candida albicans* ATCC 24433, B: *C. glabrata* ATCC 90030, C: *Candida krusei* ATCC 6258, D: *Candida parapsilosis* ATCC 22019. Most active compounds are highlighted in red and moderately active compounds are written in bold.

Compounds **4d**, **4i**, **4k**, **4l**, and **4m** were more effective than other derivatives against *C. albicans* (ATCC 24433). The MIC_90_ value of these five compounds was 1.95 μg/mL, and the MIC_90_ value of compounds **4e** and **4n** was determined as 3.91 μg/mL. The other analogues did not show anticandidal activity.

All compounds except **4j** showed cytotoxicity against *C. glabrata* (ATCC 90030). Compounds **4b**, **4e**, and **4f** showed extraordinary anticandidal activity when compared with other analogues and ketoconazole. Their MIC_90_ values were determined less than 0.06 μg/mL. Besides that, the MIC_90_ values of compounds **4a**, **4d**, **4i**, and **4k**–**n** were 1.95 μg/mL. The anticandidal activity against *C. glabrata* (ATCC 90030) was seen at 15.63 μg/mL for **4g**, at 31.25 μg/mL for **4h**, and 62.50 μg/mL for **4c**.

The MIC_90_ values of **4h** and **4m** against *C. krusei* (ATCC 6258) were ≤0.06 μg/mL. However, the MIC_90_ values of compounds **4d**, **4i**, **4k**, and **4n** were 1.95 μg/mL, 3.91 μg/mL for **4f**, 31.25 μg/mL for **4a**–**c** and **4g**; and 62.50 μg/mL for **4e** and **4j**. Despite the very high cytotoxicity of the 13 derivatives, compound **4l** did not show cytotoxicity.

All compounds have anticandidal effects against *C. parapsilosis* (ATCC 22019) compared to other *Candida* species. In particular, compound **4j** has even higher efficacy on *C. parapsilosis* (ATCC 22019) than the reference drug, although it did not show activity against *C. albicans* (ATCC 24433) and *C. glabrata* (ATCC 90030) cells. The MIC_90_ value of **4j** was less than 0.06 μg/mL. In addition, compound **4m** (MIC_90_: 0.24 μg/mL) showed significant cytotoxicity against other *Candida* species.

As a result, compounds **4d**, **4e**, **4i**, **4k**, **4m**, and **4n** showed anticandidal activity at very low concentrations against all *Candida*. Specifically, the chlorine (**4b, 4h**) substitution at the para or meta positions, the cyano group (**4e**) at the para position, the methyl group (**4f**) at the meta position, the flour atom (**4j**) at the meta position of the phenyl ring, or naphthalene-1-yl (**4m**) substitution were marked as remarkable derivatives.

Antifungal activity against *C. albicans* (ATCC 24433) did not increase, even more, and disappeared in the presence of substitution on the phenyl ring. The anticandidal activity was preserved when pyridine or naphthalene was substituted instead of the phenyl derivatives.

When phenyl was substituted at the meta position, anticandidal activity against *C. glabrata* (ATCC 90030) was preserved or disappeared. Furthermore, the substitutions of 4-Cl, 4-CN, or 4-CH_3_ phenyl increased the activity tremendously. In both the third and fourth position substitutions, the electron-donating groups showed higher activity than the electron-withdrawing groups. It was observed that the activity was preserved in the replacement of phenyl with other rings. Compounds showing selectivity for this species were identified as **4b**, **4e**, and **4f**.

The effect of para substitution of the phenyl on the antifungal activity against *C. krusei* (ATCC 6258) resulted in decreased efficacy. In addition, the electron-donating groups showed higher activity than the electron-withdrawing groups similar to anti-*C. glabrata*. The activity vanished by replacing the phenyl with a pyridine, and in contrast, replacing the phenyl with a naphthalene-1-yl caused the activity to increase significantly. Furthermore, using naphthalene-2-yl instead of phenyl resulted in the activity being preserved. This strain was found to be sensitive to compounds **4h** and **4m**.

In the analysis of the anticandidal activity against *C. parapsilosis* (ATCC 22019), the presence of an electron-donating group at the para position of the phenyl ring was observed to increase the activity 4 times compared to the meta position substitutions. However, 3- or 4-chloro substitution on the phenyl reduces the activity. If the group or atom could make a hydrogen bond and it is at the third position of the phenyl, then it could increase the activity 8-fold; otherwise, the activity decreases 8-fold. Otherwise, if the substitutions could form the hydrogen bond, then the activity of these substitutions could be 64 times more than other substitutions. Besides that, replacing the phenyl ring with a pyridine and naphthalene-2-yl moieties or with naphthalene-1-yl moiety increased the activity 8 times or 64 times, respectively. Compound **4j** was identified as a selective molecule for this species.

When the results were evaluated for all fungi species, it was determined that there was no difference between the electron-donating or -withdrawing substituents. Moreover, compounds (**4i** and **4k**) having 3-NO_2_ and 3-OCH_3_ groups showed similar anticandidal activity at the same concentrations against all fungi species. Naphthalen-1-yl substitution (**4m**) was determined as the most active compound against all fungal cells.

### 2.4 Results of Cytotoxic Effects on NIH/3T3 Cell Line

The cytotoxicity effect of the compounds was tested against NIH/3T3 healthy cell line and determined as IC_50_ (μM) (displayed in Table 4).

**Table 4:** IC_50_ (μM) of the Compounds against NIH/3T3 Cells

compound	IC_50_ ± STD	compound	IC_50_ ± STD
**4a**	39.83 ± 1.45	**4h**	14.48 ± 0.57
**4b**	14.72 ± 0.34	**4i**	8.54 ± 0.26
**4c**	31.60 ± 0.87	**4j**	10.00 ± 0.39
**4d**	1.26 ± 0.033	**4k**	12.04 ± 0.44
**4e**	>1000	**4l**	>1000
**4f**	>1000	**4m**	34.51 ± 0.88
**4g**	10.40 ± 0.22	**4n**	>1000

The results can be sorted from the most cytotoxic to the less cytotoxic compounds as **4d** > **4i** > **4j** > **4g** > **4k** > **4h** > **4b** > **4c** > **4m** > **4e** = **4f** = **4l** = **4n** against the NIH/3T3 healthy cell line. In fact, except for compound **4d**, it can be suggested that all compounds were found safe considering that their MIC_90_/IC_50_ ratio is less than 1. Moreover, IC_50_ values of compounds **4e**, **4f**, **4l**, and **4n** were higher than 1000 μM.

### 2.5 Results of Enzyme Inhibition Tests

#### 2.5.1 DNA-Gyrase Enzyme Inhibition

**Figure 2:**
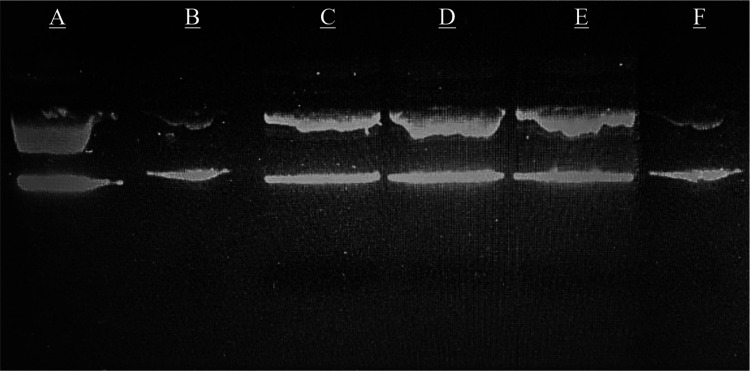
Inhibition visualities of compounds **4g** and **4m** and ciprofloxacin against *E. coli* DNA gyrase by electrophoresis. (A) Relax (pHOT1) DNA image: no chemicals and DNA gyrase; (B) supercoiled DNA image: relaxed (pHOT1) DNA with gyrase, no chemicals; (C) compound **4g**: relax (pHOT1) DNA, gyrase, and chemical dissolved in dimethyl sulfoxide (DMSO); (D) compound 4m: relax (pHOT1) has DNA, gyrase, and chemical dissolved in DMSO, (E) positive control: there is relax (pHOT1) DNA, gyrase, and ciprofloxacin dissolved in DMSO; and (F) negative control: there is relax (pHOT1) DNA, gyrase, and DMSO only.

The inhibition activity on the DNA-gyrase enzyme was performed *via* an electrophoretic method for ciprofloxacin, compounds **4g** and **4m**. The obtained results are displayed in Figure 2.

In the figure, the reference drug and the synthesized compounds have very similar appearances, and there was no peak belonging to the supercoiled DNA. Thus, it can be concluded that the antibacterial effect of compounds **4g** and **4m** was caused by the inhibition of the DNA-gyrase enzyme.

#### 2.5.2 Lanosterol 14α-Demethylase (LMD, CYP51A1) Inhibition Tests

The activity of the target compounds was tested against *Candida* strains *via* the determination of ergosterol production. The results are shown in Table 5.

**Table 5:** CYP51A1 Inhibition% Results of the Final Compounds (μg/mL)

	*C. glabrata*			
	concentrations (μg/mL)			
compounds	3.91	0.98	0.24	0.06
**4b**	70.345 ± 2.638	60.197 ± 2.095	51.754 ± 1.612	36.198 ± 0.965
**4e**	25.398 ± 0.832	21.721 ± 0.955	18.289 ± 0.785	16.680 ± 0.774
**4f**	79.524 ± 2.141	65.073 ± 1.676	53.774 ± 2.390	42.132 ± 1.561
ketoconazole	89.368 ± 3.236	72.768 ± 2.868	61.856 ± 1.833	50.422 ± 1.162
fluconazole	86.512 ± 2.752	70.144 ± 2.052	63.679 ± 2.185	46.267 ± 0.936

	*C. krusei*			
	concentrations (μg/mL)			
compounds	3.91	0.98	0.24	0.06
**4h**	82.223 ± 2.575	67.651 ± 1.736	60.184 ± 1.985	45.530 ± 0.961
**4i**	20.021 ± 0.848	18.361 ± 0.767	14.178 ± 0.655	11.662 ± 0.479
**4m**	85.408 ± 3.502	70.574 ± 2.648	61.289 ± 1.461	48.431 ± 2.036
ketoconazole	87.657 ± 3.436	75.647 ± 2.749	60.630 ± 2.875	52.865 ± 1.662
fluconazole	85.168 ± 3.055	72.523 ± 1.961	60.132 ± 2.042	43.378 ± 0.937

	*C. parapsilosis*			
	concentrations (μg/mL)			
compounds	3.91	0.98	0.24	0.06
**4j**	75.164 ± 2.828	63.508 ± 1.948	55.281 ± 1.174	41.867 ± 0.968
**4k**	23.518 ± 1.051	17.437 ± 0.861	15.662 ± 0.616	11.449 ± 0.518
**4l**	78.667 ± 2.237	62.949 ± 2.626	56.137 ± 0.928	40.328 ± 1.326
ketoconazole	88.245 ± 2.662	71.864 ± 2.229	63.419 ± 2.074	55.348 ± 1.091
fluconazole	87.786 ± 3.147	73.204 ± 2.031	61.533 ± 1.282	49.579 ± 1.733

The test results showed that the activation mechanism of some active compounds (**4b**, **4f**, **4h**, **4j**, **4l**, and **4m**) was achieved *via* blocking ergosterol production, which eventually ruins the cell integrity. The fact that compounds **4e**, **4i**, and **4k** did not decrease the ergosterol production compared to the standard drugs led to the conclusion that their antifungal activity does not occur *via* inhibition of the LMD enzyme. As a result, we determined that even though all compounds did not show their antifungal activity *via* LMD enzyme inhibition, the active compounds (**4b**, **4f**, **4h**, **4j**, **4l**, and **4m**) showed their anticandidal activity *via* LMD enzyme inhibition. For the next study, we aimed the determination of the antifungal mechanism of compounds **4e**, **4i**, and **4k**.

#### 2.5.3 Human Aromatase (HA) Inhibition

It was mentioned in the Introduction section that antifungal agents acting *via* a mechanism that involves the LDM pathway can cause some side effects. Thus, we aim to eliminate those possible side effects, so we also tested the antifungal active compounds on HA. The results are shared in Table 6. (Only compounds with inhibiting HA enzyme IC_50_ values were shared among antifungal active compounds.)

**Table 6:** Aromatase IC_50_ Results of the Final Compounds (μM)

compound		compound	
**4i**	0.033 ± 0.001	**4k**	0.038 ± 0.001
**4j**	0.047 ± 0.001	**4l**	0.042 ± 0.002
letrozole	0.026 ± 0.001		

The IC_50_ values of the active compounds **4i**, **4j**, **4k**, and **4l** were 0.038, 0.042, 0.047, and 0.033 μM against aromatase enzyme, respectively. The IC_50_ values of these compounds were found very close to the standard drug (letrozole, 0.026 μM). The remaining active antifungal compounds did not show aromatase inhibition activity.

### 2.6 Results of *In Silico* Studies

#### 2.6.1 Results of ADME Parameters and Lipinski’s Rule of Five

The evaluation of some properties of the compounds and whether they violate Lipinski’s rule of five are given in Table 7. This rule does not determine whether the compounds are pharmacologically active but indicates that compounds that comply with the rule are less likely to be eliminated during clinical trials and thus have a better chance of reaching the market.

**Table 7:** Physicochemical, Pharmacokinetic, and Pharmaceutical Chemistry Properties of the Synthesized Compounds^a^

	PP			pharmacokinetic properties				PC	
	HBA	HBD	TPSA	Log *P*	Log *S*	MBE	Log *K*_p_	LK	SK
**4a**	5	1	87.64	4.97	–7.48	high	–4.65		3.17
**4b**	4	1	87.64	5.19	–8.02	high	–4.38		3.18
**4c**	6	1	133.46	3.91	–8.15	low	–5.01		3.25
**4d**	4	1	87.64	4.65	–7.38	high	–4.61		3.16
**4e**	5	1	111.43	4.44	–7.58	high	–4.97		3.26
**4f**	4	1	87.64	4.99	–7.75	high	–4.44		3.28
**4g**	5	1	96.87	4.65	–7.54	high	–4.82		3.31
**4h**	4	1	87.64	5.19	–8.02	high	–4.38		3.17
**4i**	6	1	133.46	3.98	–8.15	low	–5.01		3.31
**4j**	5	1	87.64	4.98	–7.48	high	–4.65		3.17
**4k**	5	1	96.87	4.66	–7.54	high	–4.82		3.34
**4l**	5	1	100.53	3.92	–6.54	high	–5.38		3.11
**4m**	4	1	87.64	5.54	–8.68	low	–4.03		3.38
**4n**	4	1	87.64	5.55	–8.68	low	–4.03		3.38
**RD**_**1**_	5	2	74.57	1.10	0.00	high	–9.09		2.51
**RD**_**2**_	5	0	69.06	3.55	–5.51	high	–6.46	+(1)	4.45

a PP: physicochemical properties, HBA: number of hydrogen bond acceptor, HBD: number of hydrogen bond donor, TPSA: topological polar surface area (Å^2^), Log *P*: partition coefficient, Log *S*: solubility coefficient in water, MBE: absorption level by the gastrointestinal system, Log *K*_p_: skin absorption coefficient (cm/sn), PC: pharmaceutical chemistry; LK: violation number of rule of five, SK: synthesis ease score in terms of medicinal chemistry (1: very easy, 10: very hard, *r*^2^: 0.94), RD_1_: reference drug-1 (ciprofloxacin), RF_2_: reference drug-2 (ketoconazole).

According to the results, the compounds are predicted to be suitable for oral use since the final molecules do not violate Lipinski’s RoF. However, it was calculated that molecules carrying NO_2_ phenyl or naphthalene moieties may have low absorption from the gastrointestinal tract. When the rates of compounds passing through the horny layer, known as the rate-limiting step, are evaluated for topical drug applications,^49^ it is predicted that all compounds are suitable for that. The synthesis feasibility of the target compounds in terms of medicinal chemistry was determined as easy compared to that of ketoconazole and difficult compared to that of ciprofloxacin. In conclusion, the prediction results of the compounds suggest that they can be used for both oral and topical uses.

#### 2.6.2 Binding Modes on DNA-Gyrase Enzyme and SAR

**Figure 3:**
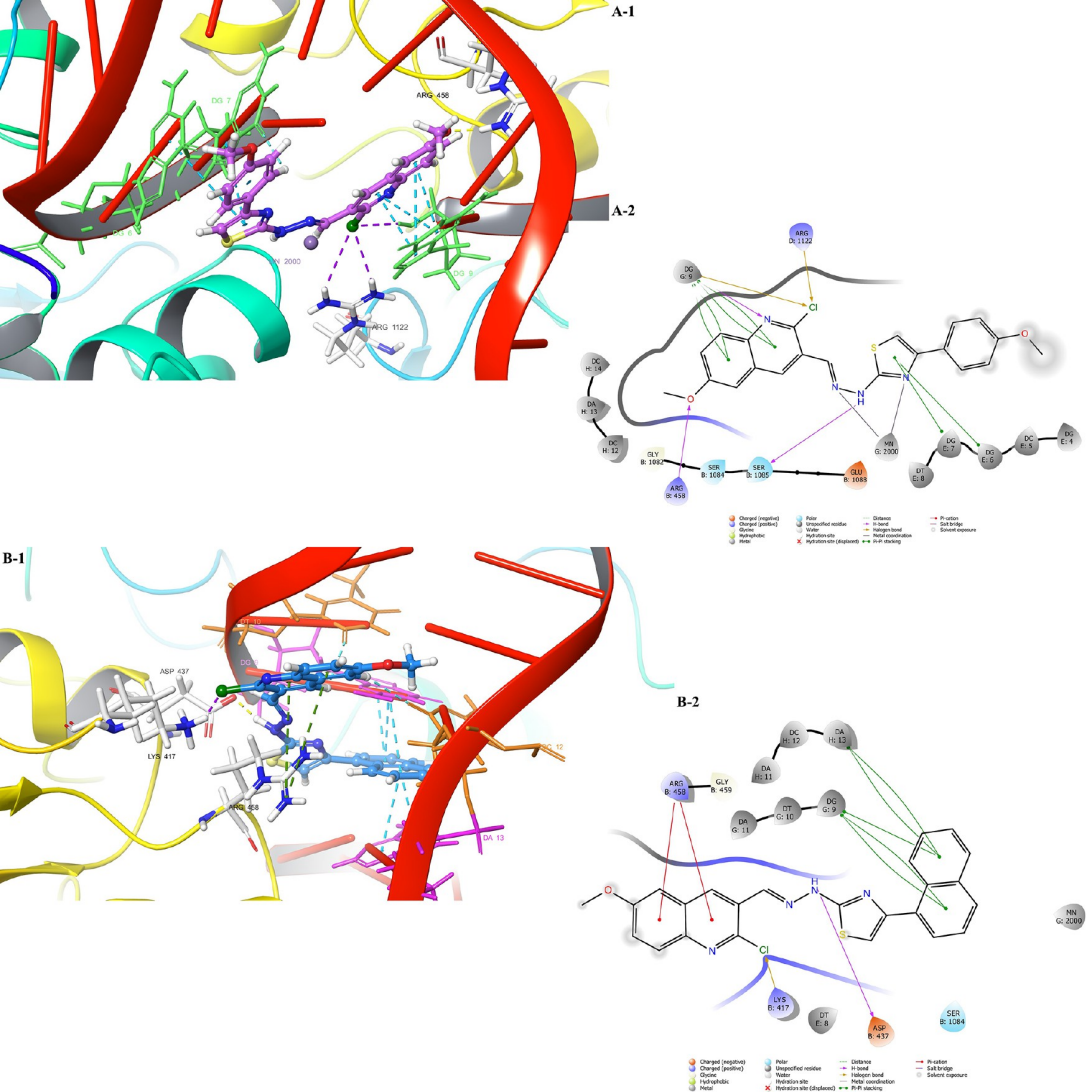
Docking poses of the antibacterial active compounds in the active side of DNA gyrase (PDB ID: 2XCT). 2D and 3D poses of the compounds are viewed. (A) Compound **4g** and (B) compound **4m**. Nucleotides are represented by the residue-type color. For clarity, only interacted residues are viewed.

The best docking poses and their interaction indices are shared in Figure 3 and Table 8.

**Table 8:** Interaction Index for the Active Compounds—DNA-Gyrase Enzyme Complex

compound	ligand moiety	residue	interaction type, count
**4g**	oxygen of quinoline methoxy	chain B: Arg458	1 H-bond
	N_2_ of hydrazone	Ser1085	1 H-bond
	chlorine of quinoline	chain D: Arg1122	1 halogen bond
	thiazole ring	chain E: DG6	1 π–π stacking
	thiazole ring	DG7	1 π–π stacking
	N of quinoline ring	chain G: DG9	1 H-bond
	chlorine of quinoline	DG9	1 halogen bond
	quinoline ring	DG9	4 π–π stackings
	N_1_ of hydrazone	Mn2000	1 metal chelate
	N of thiazole	Mn2000	1 metal chelate
**4m**	chlorine of quinoline	chain B: Lys417	1 halogen bond
	N_1_ of hydrazone	Asp437	1 H-bond
	quinoline ring	Arg458	2 π–cation interactions
	naphthalene ring	chain G: DG9	3 π–π stackings
	naphthalene ring	chain H: DA13	2 π–π stackings

According to the literature,^50^ the interactions with Arg458 and Asp437 amino acids have an important impact on the inhibition activity of DNA gyrase since those connections can stabilize both the DNA and gyrase enzyme; thus, the complex will not go under the supercoiled state. On the other hand, a divalent metal atom (Mg^2+^) is also important for DNA-gyrase activation since it is believed to be necessary to both cleave and re-ligate the DNA. In this study, both compounds interacted with Arg458, but only compound **4m** formed a H-bond with the Asp437 residue, and only compound **4g** was chelated with G:Mg2000. Therewithal, both compounds made hydrophobic interactions with different nucleotides (G:DG6, DG7, DG9, and H:DA13), but a common one was the DG9 nucleotide. Moreover, compound **4g** formed a H-bond with this nucleotide. All of these differences are probably caused by the hydrophobic part of the substituents. Because the naphthalene ring (**4m**) can interact with nucleotides *via* π–π stacking, it can most likely be easily localized. Besides that, the 4-methoxyphenyl moiety (**4g**) was under solvent exposition. In conclusion, both compounds showed interest in the DNA-gyrase complex differently, but they occupied the same pocket of the complex to bind. And these interactions are in harmony with the *in vitro* results.

**Figure 4:**
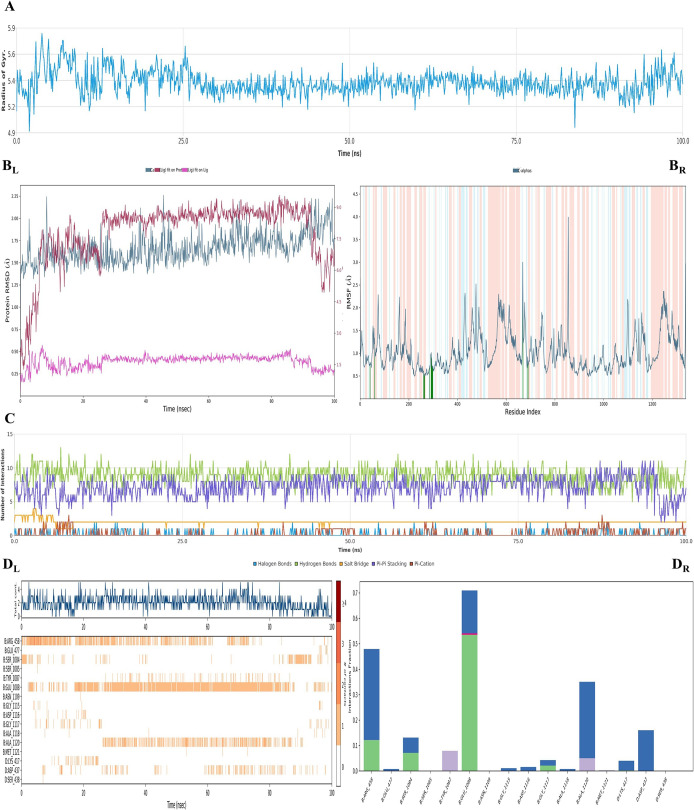

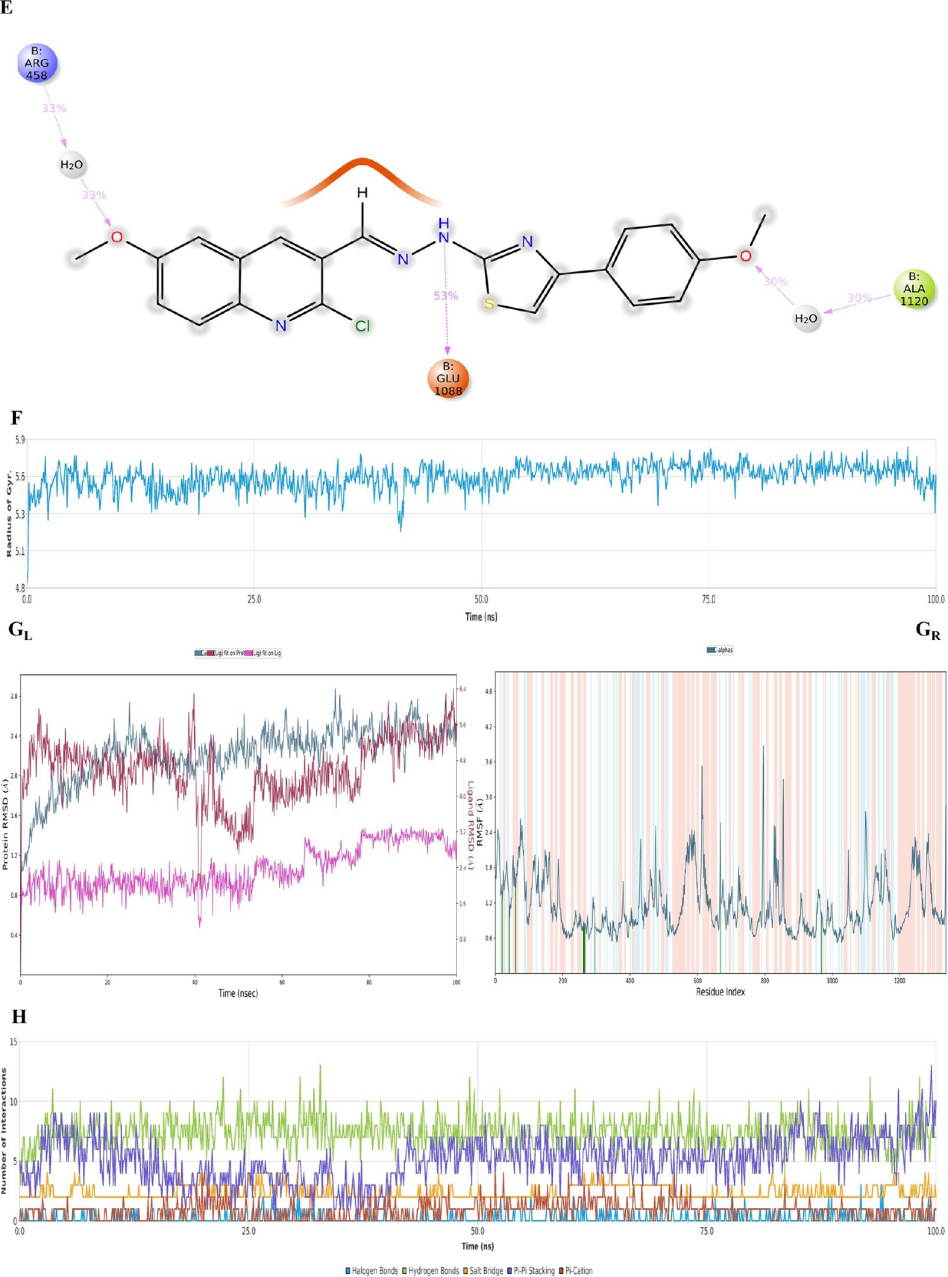

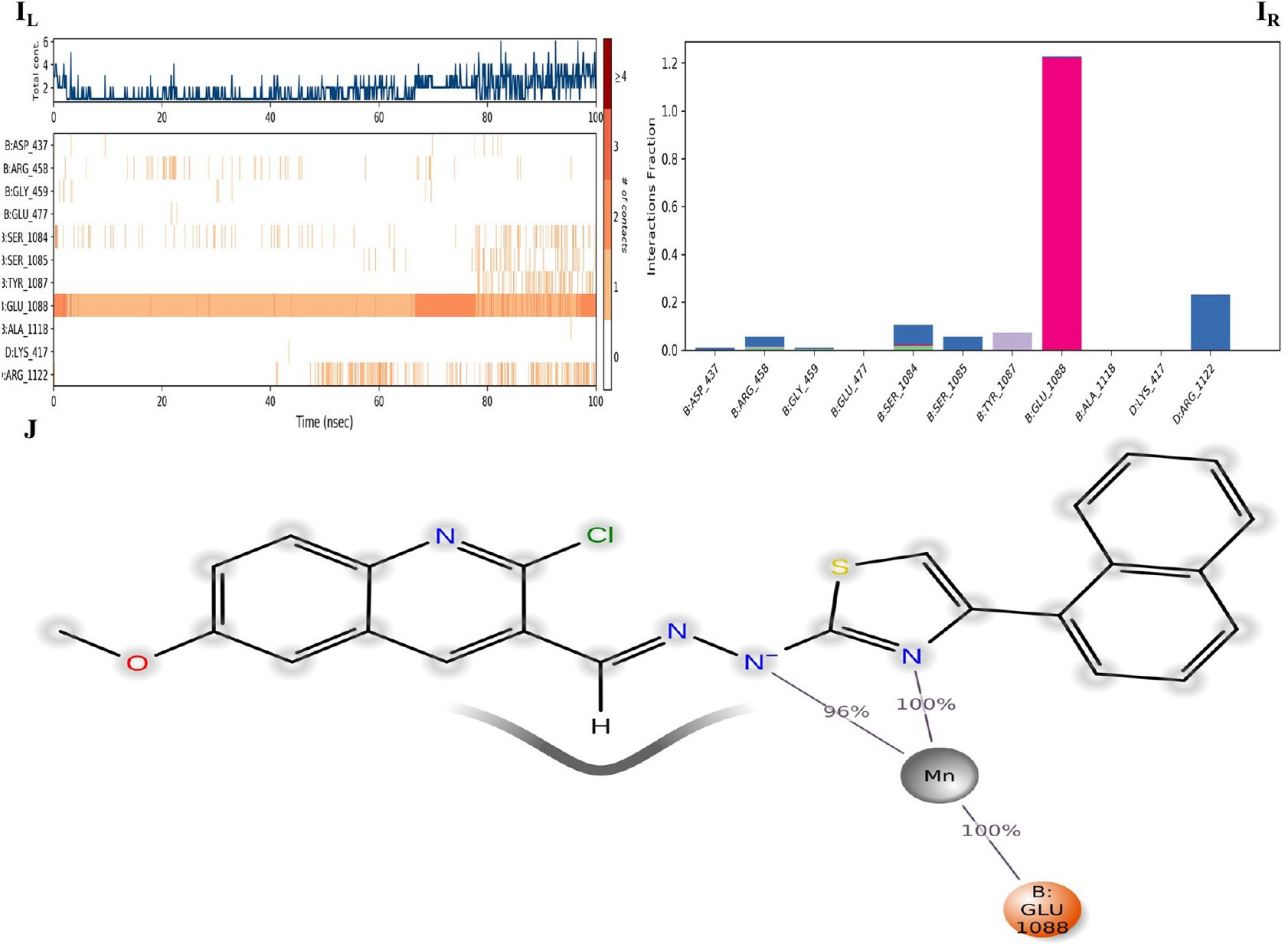
Plots of the MDS results for **4g**– and **4m**–DNA–DNA-gyrase complexes. The stability properties (*R*_g_, root-mean-square deviation (RMSD), and root-mean-square fluctuation (RMSF) plots, respectively) are shown in (A), (B), (F), and (G) sections. The interaction properties ((C, H) number of interactions–interaction types–time plot; (D_R_, I_R_) interaction fraction–residue diagram; (D_L_, I_L_) total connections–residues–time plot; (E, J) 2D interaction pose with connection strength (cutoff = 0.2) at the active region) are in (C)–(E) and (H)–(J) sections, respectively.

To understand the effects of environmental changes and clarify SAR more specifically, the molecular dynamics simulations (MDS) study was performed for both compounds (**4g** and **4m**). The results are shown in Figure 4.

For both complexes, the stability of the systems was preserved, as shown in Figure 4A,B,F,G. The primer influential residue for the stabilization was determined as Glu1088. However, compounds **4g** and **4m** displayed different localizations, similar to docking studies, and thus their interacted parts with Glu1088 differed, as shown in Figure 4C–E,H–J. The hydrazone moiety of compound **4g** directly interacted with Glu1088, while both the hydrazone moiety and thiazole nitrogen of compound **4m** interacted *via* metal-chelating with the same residue. The bond strength of compound **4m** was greater than that of **4g**’s. On the other hand, compound **4m** also interacted with Arg1122 *via* water-mediated H-bond even if it was just a pinch, but it did not show sufficient affinity to Arg458 or other amino acids as found in the docking study; hence, it suggests that its inhibition activity was rooted in metal-chelating. On the contrary, compound **4g** formed two water-mediated H-bonds with Arg458 and Ala1120, yet all of its interactions were not found to be stable at the end of the simulation. Besides that, since the interactions with DNA nucleotides are not generated as the plot by the program, those interactions can be viewed in Video 1_4g and Video 1_4m. As seen, both compounds have interacted with DT:8, DG:9, DC:12, and DA:13 nucleotides *via* π–π stackings. Especially, compound **4g** stacked to DG:9 nucleotide; therefore, we suggest that its activity was based on this connection. So, as a result, those compounds were in good relationship with the DNA; hence, it was concluded that they poisoned the DNA nucleotides to show their function in the same way as fluoroquinolone drugs.

#### 2.6.3 Binding Modes on Lanosterol 14α-Demethylase Enzyme and SAR

**Figure 5:**
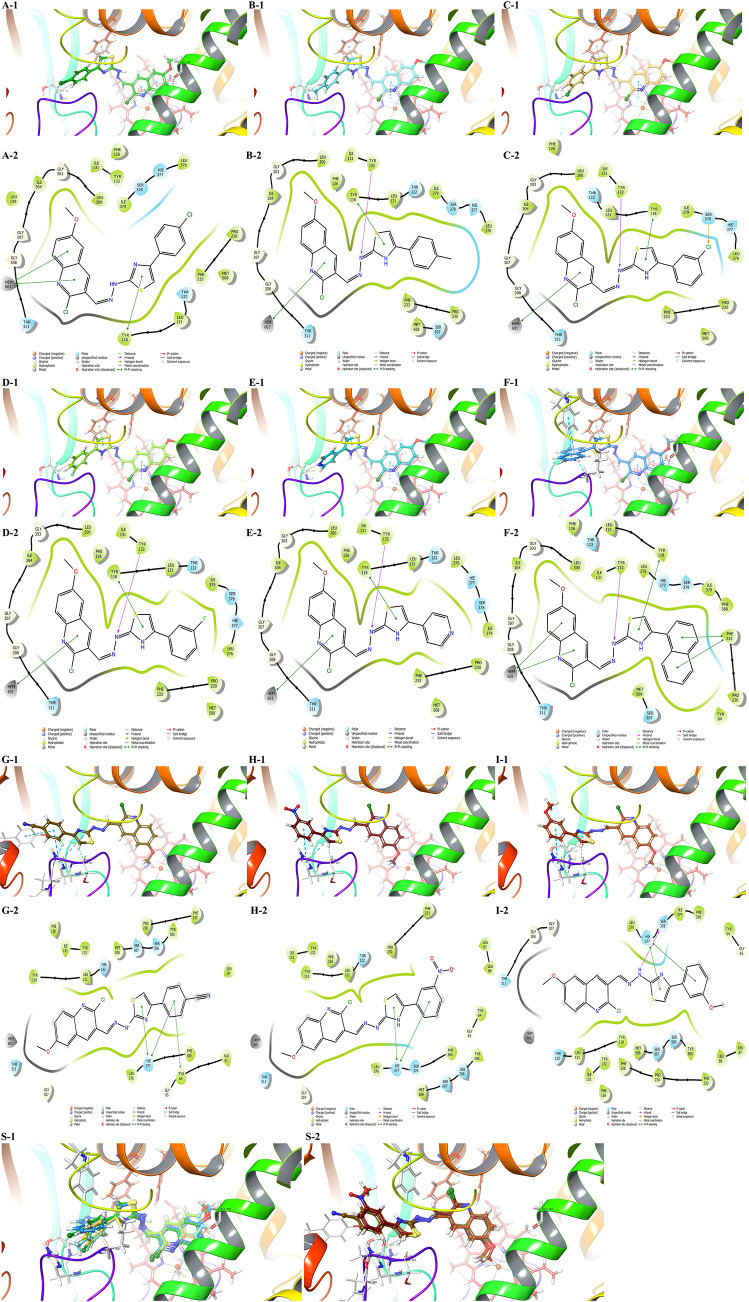
Docking poses of the antifungal active compounds in the active side of the LDM enzyme (PDB ID: 5TZ1). 2D and 3D poses of the compounds were viewed. (A) Compound **4b**; (B) compound **4f**; (C): compound (**4h**); (D) compound **4j**; (E) compound **4l**; (F) compound **4m**; (G) compound **4e**; (H) compound **4i**; and (I) compound **4k**. (S-1) 3D superimposed of the active compounds against the CYP51A1 enzyme; (S-2) 3D superimposed of the inactive compounds against the CYP51A1 enzyme.

The best docking poses and their interaction indices are shared in Figure 5 and Table 9.

**Table 9:** Interaction Index for the Active Compounds—Lanosterol 14α-Demethylase Enzyme Complex

compound	ligand moiety	residue	interaction type, count
**4b**	thiazole ring	Tyr118	1 π–π stacking
	H_7_ of the quinoline ring	Gly303	1 ar H-bond
	H_2_ of the phenyl ring	Ser378	1 ar H-bond
	quinoline ring	HEM601	2 π–π stackings
**4f**	thiazole ring	Tyr118	1 π–π stacking
	N_1_ of hydrazone	Tyr132	1 H-bond
	H_3_ of the phenyl ring	Ser378	1 ar H-bond
	quinoline ring	HEM601	2 π–π stackings
**4h**	thiazole ring	Tyr118	1 π–π stacking
	N_1_ of hydrazone	Tyr132	1 H-bond
	Cl of the phenyl ring	Ser378	1 halogen bond
	quinoline ring	HEM601	2 π–π stackings
**4j**	thiazole ring	Tyr118	1 π–π stacking
	N_1_ of hydrazone	Tyr132	1 H-bond
	H_2_ of the phenyl ring	Ser378	1 ar H-bond
	quinoline ring	HEM601	2 π–π stackings
**4l**	thiazole ring	Tyr118	1 π–π stacking
	N_1_ of hydrazone	Tyr132	1 H-bond
	H_3_ of the phenyl ring	Ser378	1 ar H-bond
	quinoline ring	HEM601	2 π–π stackings
**4m**	thiazole ring	Tyr118	1 π–π stackings
	H_2_ of the naphthalene ring	Tyr118	1 ar-H-bond
	N_1_ of hydrazone	Tyr132	1 H-bond
	Naphthalene ring	Phe233	2 π–π stackings
	H_7_ of the quinoline ring	Gly303	1 ar H-bond
	H_7_ of the naphthalene ring	Met508	1 ar H-bond
	quinoline ring	HEM601	3 π–π stackings
**4e**	phenyl ring	Tyr64	1 π–π stacking
	phenyl ring	His377	1 π–π stacking
	thiazole ring	His377	1 π–π stacking
	H_2_ of the phenyl ring	Ty505	1 ar H-bond
	H_5_ of the thiazole ring	Ser507	1 ar H-bond
**4i**	phenyl ring	His377	1 1 π–π stacking
	thiazole ring	His377	1 π–π stacking
	H_5_ of the thiazole ring	Ser507	1 ar H-bond
**4k**	phenyl ring	His377	1 1 π–π stacking
	thiazole ring	His377	1 π–π stacking
	N_1_ of hydrazone	Ser378	1 ar H-bond
	H_5_ of the thiazole ring	Ser507	1 ar H-bond

Tyr118 residue and HEM protein, according to the literature,^51^ play critical roles in inhibition activity. Additionally, Tyr132, Gly303, and Ser378 amino acids are defined as ligand-contacting residues and they have an additional role for different fungal species. Moreover, Met508 is identified as a substrate-binding region like Tyr118 and Tyr132, but it is not responsible for supporting HEM protein. That is why it is suggested that among the other active pocket amino residues, Tyr118 and HEM are essential for the activity, but alone their interaction with β4 hairpin amino acids (like Ser507 and Met508) is not sufficient to inhibit the enzyme activity. However, the interaction with the β4 hairpin region may increase the activity.^52^

In this study, the results revealed that the active compounds against CYP51A have two common interactions. One was found between the thiazole ring and Tyr118 amino acid, and the other was found between the quinoline ring and HEM601, both of which were π–π stackings. Additionally, the hydrazone moiety of the active compounds formed one H-bond with Tyr132 residue, except **4b**; nonetheless, compound **4b** has two aromatic H-bonds in addition to two common interactions with Gly303 and Ser378 amino acids. As mentioned above, all interactions support the results of antifungal tests and enzyme activities.

Besides, compounds **4e**, **4i**, and **4k** could not sit in the active pocket due to their substitutions. Therefore, their action mechanism should go on another pathway. All three have the capability to form a H-bond. Also, docking results point out that they were under solvent exposure; in other words, maybe some of their physicochemical properties such as TPSA and lipophilicity are not proper to the CYP51A enzyme.

In conclusion, the hybridization of the quinoline, hydrazone, and 4-arylthiazole moieties is significantly effective on this enzyme, which explains how they interacted. Also, 2-chloro-6-methoxyquinolin ring was marked as a pharmacophore group since it contacted the HEM protein.

**Figure 6:**
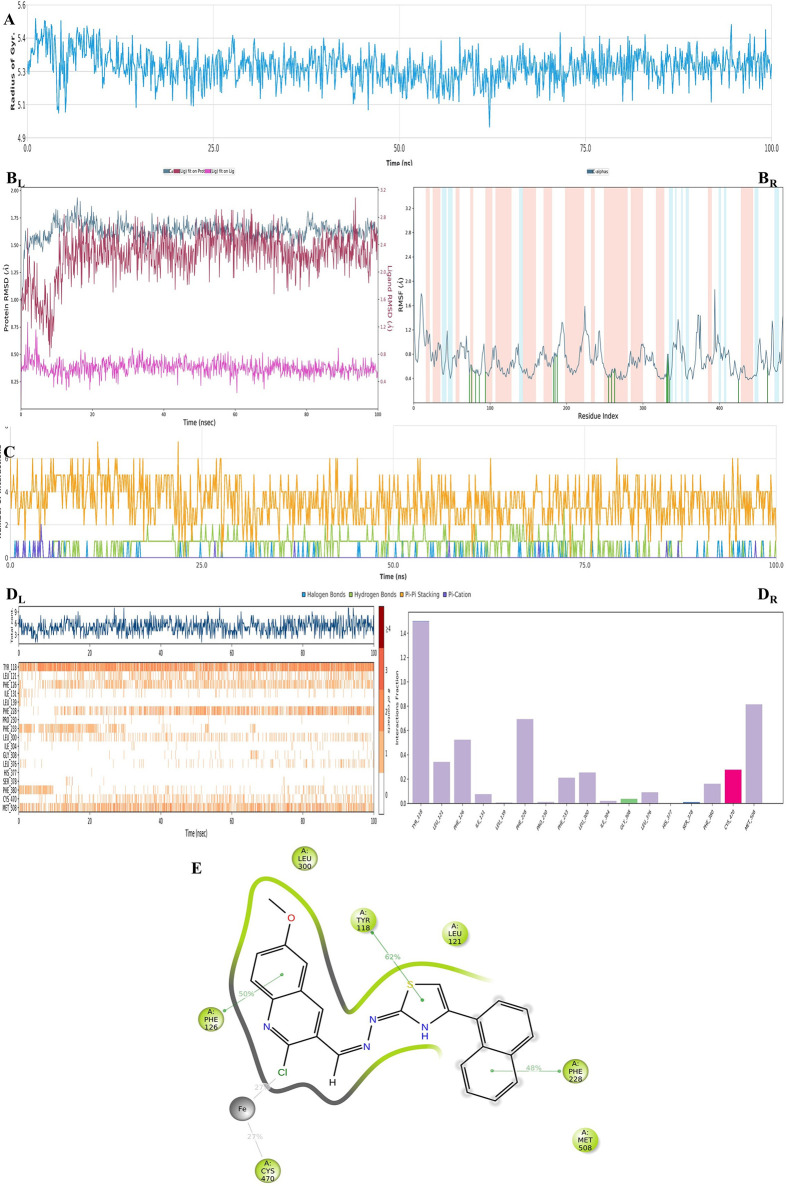
Plots of the MDS results for compound **4m**–LDM enzyme complex. The stability properties (*R*_g_, RMSD, and RMSF plots, respectively) are in (A) and (B) sections. The interaction properties ((C) number of interactions–interaction types–time plot; (D_R_) interaction fraction–residue diagram; (D_L_) total connections–residues–time plot; and (E) 2D interaction pose with connection strength (cutoff = 0.2) at the active region) are in (C)–(E) sections.

For further understanding of the SAR, MDS analysis was performed using the best pose of the **4m**–LDS enzyme complex, and this complex was used as a pattern for other active analogues. The results are shown in Figure 6.

As seen in Figure 6A,B, the complex was stable during the entire simulation. The firm interactions were observed between the ligand and Tyr118 (helix B′), Phe228 (helix F″), and Met508 (β4 hairpin) residues. Chelating with the iron of the HEME protein starts approximately 10 ns after the interaction with Phe228 amino acid, and at the same time, the interaction with Phe380 is over. The RMSD plot and Video 2 clearly show that compound **4m** has undergone a conformational change. As with the molecular docking result, the interactions between Tyr118 and HEM protein (and also its iron) were confirmed by the MDS study for the importance of its inhibition activity. Aromatic substitution (in this case, a 4-naphthalenylthiazole ring was used to exemplify their analogues) interacted with Phe228. This residue is a part of the substrate access channel,^52^ and our study showed that this access point was blocked by the naphthalene moiety of the ligand. Therefore, we foresee that the bulky, aromatic groups may increase inhibition activity *via* occluding the access channel. Although our docking results did not show this interaction with any active compounds, this interaction was confirmed in MDS results for compound **4m**. It will probably result in similar interaction between the phenyl ring and Phe228 for the other active compounds.

In conclusion, the 2-chloro-6-methoxyquinoline ring and its thiazole derivatives whose structural importance was explained in *in silico* studies have great potential against the LMD enzyme. For further studies, these moieties will be reconsidered and optimized according to the above information.

#### 2.6.4 Aromatase Enzyme Binding Modes and SAR

**Figure 7:**
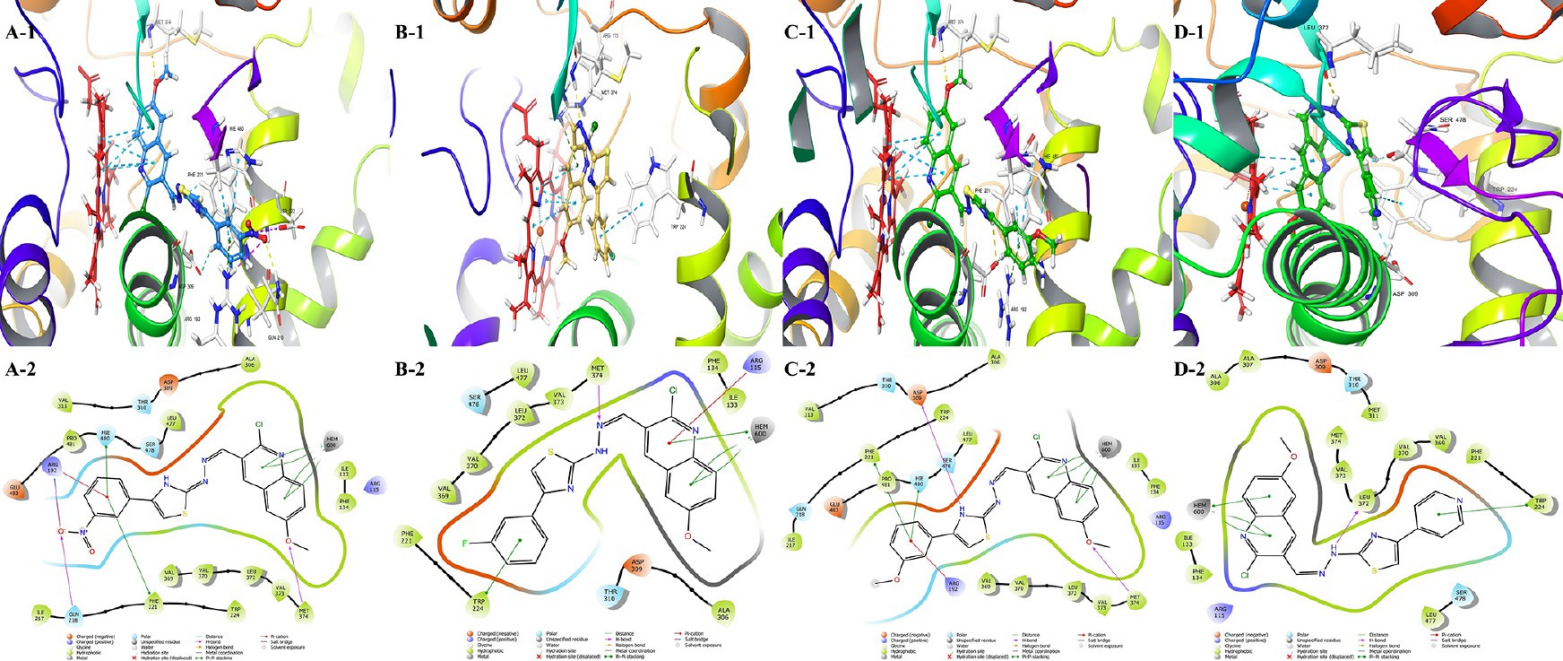
Docking poses of the active compounds in the active side of the aromatase enzyme (PDB ID: 3EQM). 2D and 3D poses of the compounds were viewed. (A) Compound **4i**, (B) compound **4j**, (C) compound **4k**, and (D) compound **4l**.

The best docking poses and their interaction indices are shared in Figure 7 and Table 10.

**Table 10:** Interaction Index for the Active Compounds—Aromatase Enzyme Complex

compound	ligand moiety	residue	interaction type, count
**4i**	NO_2_ group of the phenyl ring	Ar192	1 salt bridge
	phenyl ring	Arg192	1 π–cation interaction
	NO_2_ group of the phenyl ring	Gln218	1 H-bond
	phenyl ring	Phe221	1 π–π stacking
	N_1_H of hydrazone	Phe221	1 Ar H-bond
	NO_2_ group of the phenyl ring	Asp222	1 salt bridge
	C_5_H of the phenyl ring	Asp309	1 Ar H-bond
	methoxy group of the quinoline ring	Met374	1 H-bond
	phenyl ring	His480	1 π–π stacking
	NO_2_ group of the phenyl ring	His480	1 Ar H-bond
	quinoline ring	HEM600	5 π–π stackings
**4j**	quinoline ring	Ar115	1 π–cation interaction
	phenyl ring	Trp224	1 π–π stacking
	N_1_ of hydrazone	Met374	1 H-bond
	quinoline ring	HEM600	4 π–π stackings
**4k**	phenyl ring	Arg192	1 π–cation interaction
	phenyl ring	Phe221	1 π–π stacking
	NH of the thiazole ring	Asp309	1 H-bond
	C_5_H of the phenyl ring	Asp309	1 Ar H-bond
	methoxy group of the quinoline ring	Met374	1 H-bond
	phenyl ring	His480	1 π–π stacking
	NO_2_ group of the phenyl ring	His480	1 Ar H-bond
	quinoline ring	HEM600	4 π–π stackings
**4l**	pyridine ring	Trp224	1 π–π stacking
	C_5_H of the pyridine ring	Asp309	1 Ar H-bond
	N_1_H of hydrazone	Leu372	1 H-bond
	C_2_H of the pyridine ring	Ser478	1 Ar H-bond
	quinoline ring	HEM600	3 π–π stackings

According to the works of the literature,^53,54^ the substrate is held above the HEME protein by the 3′-flanking loop region (Pro368–Met374), the I helix (between Glu302 and Thr310), the B′–C loop (containing Ile133 and Phe134), and the four sheets (containing Ser478 and His480). Therefore, in addition to interactions with the HEM protein, interactions with these amino acids are also important to show the ligands’ inhibition activity. Also, Met374, Asp309, and Thr310 amino acids were identified as the most important residues in the active regions. Our docking study showed that all active compounds (**4i**–**l**) interacted with HEM600 (π–π stackings). Compounds **4j** and **4l** have also interacted with Trp224 as common residues. Besides, only compound **4j** interacted with Arg115, and only compound **4l** formed a H-bond with Leu372. Moreover, only compound **4l** did not form a H-bond with Met374 amino acid, but instead of that, compound **4l** interacted with Leu372, which is a member of the same β-strand region. The most active compounds **4i** and **4k** were fit at the aromatase enzyme active region very similarly. Both interacted with Ar192, Phe221, Asp309, Met374, His480, and HEM600. The differences between them are the interaction numbers of the above residues and interactions with Gln218 and Asp222. Only compound **4i** forms H-bonds with these residues, so its inhibitory effect may be increased because of that. As a result, *in vitro* results are supported by the docking results, and the *in silico* study gave a first sight of the structure–activity relationship at the molecular level.

**Figure 8:**
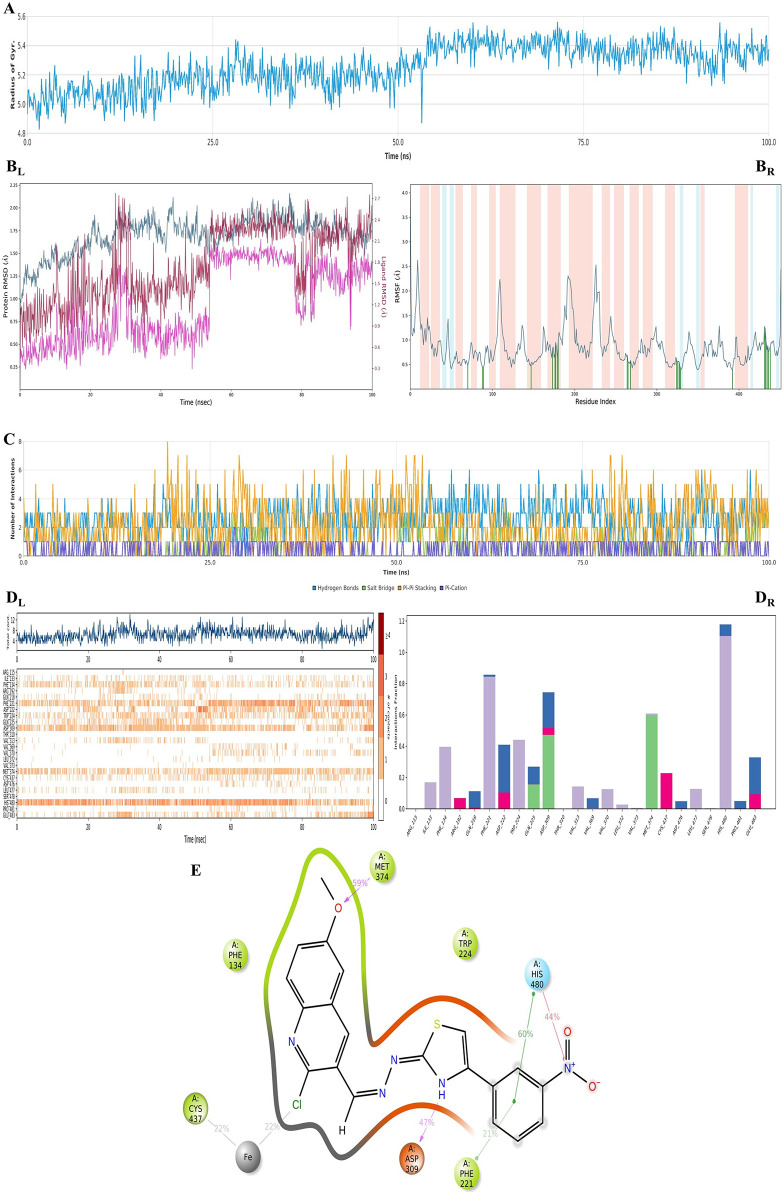
Plots of the MDS results for compound **4i**–aromatase enzyme complex. The stability properties (*R*_g_, RMSD, and RMSF plots, respectively) are in (A) and (B) sections. The interaction properties ((C) number of interactions–interaction types–time plot; (D_R_) interaction fraction–residue diagram; (D_L_) total connections–residues–time plot; and (E) 2D interaction pose with connection strength (cutoff = 0.2) at the active region) are in (C)–(E) sections.

For further understanding of the SAR, MDS analysis was performed using the best pose of the **4i**–aromatase enzyme complex, and this complex was used as a pattern for other active analogues. The results are shown in Figure 8.

Figure 8A,B points out that the stability of the complex was protected during the simulation. According to the inhibition mechanism of the aromatase enzyme, the effect of ligands has been related to either stabilization of the loop amino acids or stabilization of other regional amino acids on the active site of the enzyme that interacted with loop amino acids. Therefore, stability is mainly related to the stabilization of the loop regions. In this case (see Figure 8C–E and Video 3), it can be suggested that Arg309 (I helices), Met374 (3′-flanking loop), and His480 (β4 sheet) residues have a pivotal role considering their bond strength and their interaction continuity. Therefore, enzyme substrates cannot achieve the active region for aromatization to be ergosterol. Furthermore, the chlorine of compound **4i**’s quinoline ring chelated with the iron of the HEM protein, which is critical for the inhibition activity of the aromatase enzyme. In conclusion, the 2-chloro-6-methoxyquinoline ring was declared as the pharmacophore structure for aromatase inhibition, and its mechanism was explained.

## 3 SAR Summary

The compounds were designed and synthesized for a good antimicrobial activity profile with fewer side effects. Even though the primary aim was to find antibacterial activity with less toxicity against healthy cells, we found that their antifungal activity was more distinctive. Thus, our study was shaped by the antifungal activity after this step. For this, the active compounds were tested for their inhibition effect against LDM, and the possible side effects of the compounds were determined *via* testing the active compounds against the aromatase enzyme in addition to their cytotoxicity profile on healthy cells. After elimination by various tests, only compounds **4b**, **4f**, **4h**, and **4m** have passed the tests as antifungal agents. We suggest that these compounds can be used as antifungal agents after the completion of all testing studies. Moreover, mechanistic studies showed that the core structure of 2-chloro-6-methoxyquinoline and thiazole hybridization has great antifungal potential with fewer side effect profiles unless the substitutions are not in the third position on the phenyl ring or heteroaromatics. The bulky groups (such as 1-naphthalene) also affected the activity positively. On the other hand, all of these unfavorable groups increased the inhibitory activity of the aromatase enzyme. Thus, mentioned as unfavorable, third substitutions can be used in special cases who are suffering from fungal infections in addition to primer diseases such as cancer and transplant patients, but it is not the aim of this study. Hence, those purposes should be evaluated for further study.

## 4 Conclusions

A new series of quinoline derivatives were designed, synthesized, and analyzed. The final molecules were evaluated for their antimicrobial properties and then clarified their action mechanisms using various *in vitro* and *in silico* methods. Their antimicrobial activity was tested against six bacteria and four *Candida* species. Their cytotoxicity effects were determined against NIH/3T3 cell lines, and their action mechanisms were investigated on DNA gyrase, LDM, and aromatase enzyme. All of these results were inspected at the molecular level by docking and dynamic simulation studies and clarified their action mechanisms. Compounds **4b**, **4f**, **4h**, and **4m** have a good antifungal profile with low side effects, while compounds **4j** and **4l** can be used alone for special patients suffering from fungal infection in addition to primer disease considering their possible side effects. At least the drug interactions can be minimalized for these patients. Generally, it was announced that compound **4m** has potential antimicrobial, especially, anticandidal activity, with a trustable therapeutic index and potentially fewer side effects.

## 5 Experimental Section

### 5.1 Chemistry

All chemicals were purchased from Sigma-Aldrich Chemical Co. (Sigma-Aldrich Corp., St. Louis, MO) and Merck Chemicals (Merck KGaA, Darmstadt, Germany). All melting points (m.p.) were determined by an MP90 digital melting point apparatus (Mettler Toledo, Ohio) and were uncorrected. All reactions were monitored by thin-layer chromatography (TLC) using Silica Gel 60 F254 TLC plates (Merck KGaA, Darmstadt, Germany). Spectroscopic data were recorded with the following instruments: ^1^H nuclear magnetic resonance (NMR) Bruker DPX-300 FT NMR spectrometer, ^13^C NMR, Bruker DPX 75 MHz spectrometer (Bruker Bioscience, Billerica, MA); mass (high-resolution mass spectrometry (HRMS)) spectra were recorded on a liquid chromatography connected with hybrid ion-trap and time-of-flight mass spectrometry (Shimadzu) using electrospray ionization.

#### 5.1.1 Synthesis of 4′-Methoxyacetanilide (**1**)

4-Anisidine (0.065 mol) and triethylamine (TEA) (0.078 mol) were dissolved in dichloromethane in a flask and placed in an ice bath at 0–5 °C. On the other hand, acetyl chloride (0.09 mol) was dissolved in dichloromethane in the dropping funnel and dropped carefully into the mixture in the flask. In the meantime, care was taken to mix the mixture vigorously. After the dripping was finished, it was left to stir for another 3 h at room temperature. The end of the reaction was checked with TLC. The solvent was completely evaporated, and the solid was washed with water, filtered, and then dried. The product was crystallized from ethanol.

#### 5.1.2 Synthesis of 2-Chloro-6-methoxyquinoline-3-carbaldehyde (**2**)

*N*-(4-Methoxyphenyl)acetamide (**1**, 1 equiv) was stirred with Vilsmeier reagent in a hot water bath. After 4 h of stirring, the reaction was checked with TLC. Then, the mixture in the balloon was poured into the icy water. The solid product was washed with water, filtered, and left to dry. The dried product was crystallized from ethanol.

#### 5.1.3 Synthesis of 2-[(2-Chloro-6-methoxyquinolin-3-yl)methylene]hydrazine-1-carbothioamide (**3**)

2-Chloro-6-methoxyquinoline-3-carbaldehyde (**2**, 0.040 mol) and thiosemicarbazide (0.040 mol) were refluxed in ethanol. The reaction was terminated by TLC control. The precipitated part was filtered and separated from the alcohol. After the solvent was evaporated, the crude product was crystallized from ethanol.

#### 5.1.4 2-[2-((2-Chloro-6-methoxyquinolin-3-yl)methylene)hydrazinyl]-4-Substituted Thiazole Derivatives (**4a**–**m**)

1-Aryl-2-bromoethanone derivatives taken in equal moles with 2-[(2-chloro-6-methoxyquinolin-3-yl)methylene]hydrazine-1-carbothioamide were refluxed in alcohol for 2 h. The end of the reaction was controlled with TLC, and the final products were filtered and separated from the alcohol. After the solvent was evaporated, the crude products were recrystallized from ethanol.

**Scheme 1:**
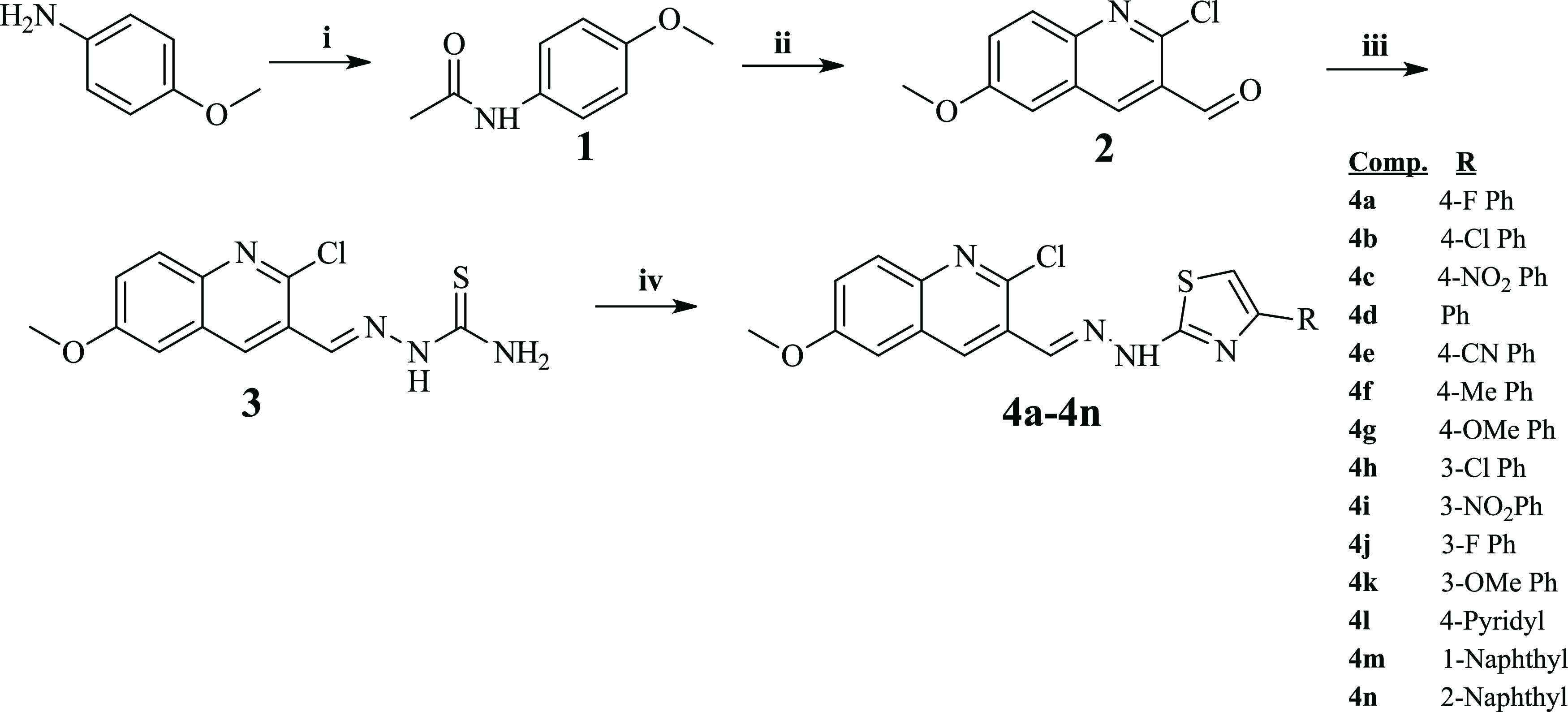
General Procedure for the Synthesis of the Compounds Reactions and conditions: (i) CH_3_COCl, tetrahydrofuran (THF), TEA, 0 °C, after dropping, room temperature (rt), 4 h; (ii) Vilsmeier–Haack reagent, 0 °C, then water bath, 8 h; (iii) thiosemicarbazide, EtOH, rt, 2 h; and (iv) 2-bromoethanone derivatives, EtOH, rt, 2 h.

The reaction procedure is displayed in Scheme 1. The synthesized compounds and their results of the spectra are shared between Sections 1.1 and 1.14 in the Supporting Information.

### 5.2 Antimicrobial Activity Studies

*E. coli* (ATCC 35218), *E. coli* (ATCC 25922), *S. aureus* (ATCC 6538), methicillin-resistant *S. aureus* (MRSA) (clinical isolate), *S. typhimurium* (ATCC 13311), and *K. pneumoniae* (NCTC 9633) cells for antibacterial effect, and *C. albicans* (ATCC 24433), *C. glabrata* (ATCC 90030), *C. krusei* (ATCC 6258), and *C. parapsilosis* (ATCC 22019) for the antifungal effect of the final compounds were used to determine the MIC_90_ values. The broth microdilution procedure specified in the M07-A9 document of the Clinical and Laboratory Standards Institute (CLSI) was used for antibacterial study,^55^ and the EDef 7.1 document published by EUCAST was used for the antifungal study.^56^ For both activity studies, the detailed procedures were explained in previous studies.^57,58^

### 5.3 Cytotoxic Evaluation of the Final Compounds

NIH/3T3 cells were incubated in Dulbecco’s modified Eagle’s medium (DMEM) (Sigma-Aldrich, St. Louis, MO), added with fetal calf serum, penicillin (100 IU/mL), streptomycin (100 mg/mL), and 7.5% NaHCO_3_ at 37 °C in a humidified atmosphere of 95% air and 5% CO_2_. NIH/3T3 cells were seeded into the 96-well plates at a density of 1 × 10^4^ cells. After 24 h of incubating period, the culture media were removed, and test compounds were added. After 24 h of incubation period, colorimetric measurements were performed by a microplate reader (Biotek) at 540 nm. Inhibition % at all concentrations was determined using the formula below, and the IC_50_ values were calculated from a dose–response curve obtained by plotting the percentage inhibition *versus* the log concentration with the use of Microsoft Excel 2013. The results were displayed as mean ± standard deviation (SD).^59−61^

### 5.4 Enzyme Inhibition Tests

#### 5.4.1 Inhibition of DNA-Gyrase Enzyme

DNA gyrase obtained from *E. coli* was used to detect the inhibition of the superhelix activity of DNA gyrase. The method was performed according to the kit protocol described by the supplier (SKU TG2000G-3, TopoGen). The detailed procedures were explained in the previous study.^62^

#### 5.4.2 Inhibition of Fungal LDM Enzyme

Ergosterol level was determined using the extract of total sterols from *C. albicans* (and its other species) following the method described by Breivik and Owades.^63^ Quantification of the ergosterol level in this extract was carried out by the recently described method.^58,64−66^ According to these described studies, in the current work, compounds **4b**, **4e**, **4f**, and **4h**–**m** were undertaken to investigate their action mechanism. Thus, the LC-MS-MS-based method that quantifies the ergosterol level was applied. All active anticandidal compounds, ketoconazole, and fluconazole were used at 3.91, 0.98, 0.24, and 0.06 μg/mL concentrations. Ergosterol quantity in negative control samples was regarded as 100%. All concentrations were analyzed in quadruplicate, and the results were expressed as mean ± standard deviation (SD).

#### 5.4.3 Inhibition of Human Aromatase Enzyme

Since targeting human aromatase enzyme was reported for antifungal activity and it plays a pivotal role in fungal cell function,^40^ compounds that showed antifungal activity were evaluated for inhibition activity on aromatase enzyme using a kit procedure (Bio Vision, Aromatase (CYP19A) Inhibitor Screening Kit (Fluorometric)), as described in previous studies.^67−69^

### 5.5 *In Silico* Studies

The pharmacokinetic profile was predicted *viain silico* methods. The active compounds may be considered in the future for *in vivo* pharmacokinetic studies as the current study is involved basically in the evaluation of the activity. Therefore, the medicinal chemistry and pharmacokinetic profiles of the designed compounds were calculated using the Swiss-ADME web-based program.^70^

All docking studies on various enzymes were performed using the Schrodinger Maestro Suite program. The interfaces of this program are used for the protein preparation process, ligand preparation process, grid generation, docking, and visualization studies.^71−73^ The crystal structure of the enzymes was retrieved from the Protein Data Bank server (PDB codes: 2XCT, 3EQM, and 5TZ1 for DNA gyrase, aromatase, and lanosterol 14α-demethylase). All ligands were set to the physiological pH (pH = 7.4) at the protonation step. The proteins were prepared according to the previous studies.^34,62,67^

The molecular dynamics simulations (MDS) were performed using the Maestro Desmond interface program.^74^ All molecular dynamics simulations (MDS) for 100 ns were carried out to analyze the stability of the identified hits from the *in vitro* docking results. Preparing the system setup, performing molecular dynamics simulations, and calculating the interaction analysis were carried out according to the same procedure as previous studies.^67,75^ All systems were set up using “System Builder” in Maestro. The complex structure was subjected to energy minimization (OPLS3e standard force field). Transferable intermolecular potential with the 3-point water model was used for the creation of the hydration model. The neutralization of the system was achieved using Na^+^ and Cl^–^ ions. The molecular dynamic simulation was performed following the completion of the system setup. The radius of gyration (*R*_g_), root-mean-square fluctuation (RMSF), and root-mean-square deviation (RMSD) values were calculated by the Desmond application.

## Supporting Information Available

Analytical spectra (HRMS, ^1^H NMR, and ^13^C NMR), analytical results, and InChI descriptions of the synthesized compounds (PDF)
DNA-gyrase–DNA complex (Video 1_**4g**) (AVI)
DNA-gyrase–DNA complex (Video 1_**4m**) (AVI)
LDM complex (Video 2) (AVI)
Aromatase complex (Video 3) (AVI)

## Supplementary Material

ao2c06871_si_001.pdf

ao2c06871_si_002.avi

ao2c06871_si_003.avi

ao2c06871_si_004.avi

ao2c06871_si_005.avi
