# Supplementary material for: Investigation of Novel Quinoline–Thiazole Derivatives as Antimicrobial Agents: In Vitro and In Silico Approaches
Source: ACS Omega. 2022 Dec 29;8(1):1410–29. doi: 10.1021/acsomega.2c06871 (PMC9835529; doi:10.1021/acsomega.2c06871)
Supplement: Supplementary file 1 — ao2c06871_si_001.pdf [file ao2c06871_si_001.pdf]

Investigation of novel quinoline-thiazole derivatives as antimicrobial agents: *In vitro* and *in silico* approaches

Asaf Evrim EVREN[a,b,*], Abdullah Burak KARADUMAN[c], Begüm Nurpelin SAĞLIK[b,d], Yusuf ÖZKAY[b,d], Leyla YURTTAŞ[b,*]

[a] Bilecik Şeyh Edebali University, Vocational School of Health Services, Department of Pharmacy Services, 11000, Bilecik, Turkey.

[b] Department of Pharmaceutical Chemistry, Faculty of Pharmacy, Anadolu University, Eskişehir 26470, Turkey.

[c] Department of Pharmaceutical Toxicology, Faculty of Pharmacy, Anadolu University, Eskişehir 26470, Turkey.

[d] Central Research Laboratory, Faculty of Pharmacy, Anadolu University, Eskişehir 26470, Turkey.

*Corresponding author(s). Asaf Evrim EVREN, Leyla YURTTAŞ

E-mail address(es): asafevrimevren@anadolu.edu.tr; asafevrim.evren@bilecik.edu.tr

lyurttas@anadolu.edu.tr

Keywords: DNA gyrase, lanosterol 14α-demethylase, aromatase, quinoline, thiazole, molecular docking, molecular dynamic simulation

1. Analytical results of the compounds

1.1. 2-{2-[(2-Chloro-6-methoxyquinolin-3-yl)methylene]hydrazinyl}4-(4-florophenyl) thiazole (4a)

M.p: 193-195°C. Appearance: yellow solid. Yield: 80%.

^{1}H-NMR (300 MHz, DMSO-*d6*): δ: 3.99 (3H, s, O-CH_3), 7.31 (2H, t, *J*= 8.92 Hz, phenyl H_3,H_5), 7.47 (H, s, thiazole H_5), 7.52 (H, dd, J_1= 2.82 Hz, J_2= 9.18 Hz, quinoline H_7), 7.68 (H, d, *J*= 2.76 Hz, quinoline H_5), 7.91 (H, d, *J*= 9.20 Hz, quinoline H_8), 7.97 (2H, q, J_1= 5.57 Hz, J_2= 8.82 Hz, phenyl $H_{2,6}$), 8.49 (H, s, -N=C-H), 8.78 (H, s, quinoline H_4), 12.68 (H, brs, =N-N-H).

^{13}C-NMR (75 MHz, DMSO-*d6*): δ: 56.24 (C, s, O-CH_3), 104.56 (C, s, thiazole C_5), 106.82 (C, s, quinoline C_5), 115.83 and 116.11 (2C, s, phenyl $C_{3,5}$), 124.39 (C, s, quinoline C_7), 126.73 (C, s, quinoline C_3), 127.95 and 128.06 (2C, s, phenyl $C_{2,6}$), 128.72 (C, s, quinoline C_8), 129.51 (C, s, quinoline C_{4a}), 131.59 (C, s, phenyl C_1), 133.61 (C, s, quinoline C_4), 143.27 (C, s, N=C), 145.83 (C, s, quinoline C_{8a}), 158.54 (C, s, quinoline C_2), 160. 50 (2C, s, quinoline C_6, thiazole C_4), 163.74 (C, s, phenyl C_4), 168.20 (C, s, thiazole C_2).

HRMS (-m/z): $[M+H]^+$: For $C_{20}H_{14}N_4OFSCl$ calculated: 413.0634, found: 413.0622

InChI=1S/C20H14ClFN4OS/c1-27-16-6-7-17-13(9-16)8-14(19(21)24-17)10-23-26-20-25-18(11-28-20)12-2-4-15(22)5-3-12/h2-11H,1H3,(H,25,26)/b23-10+

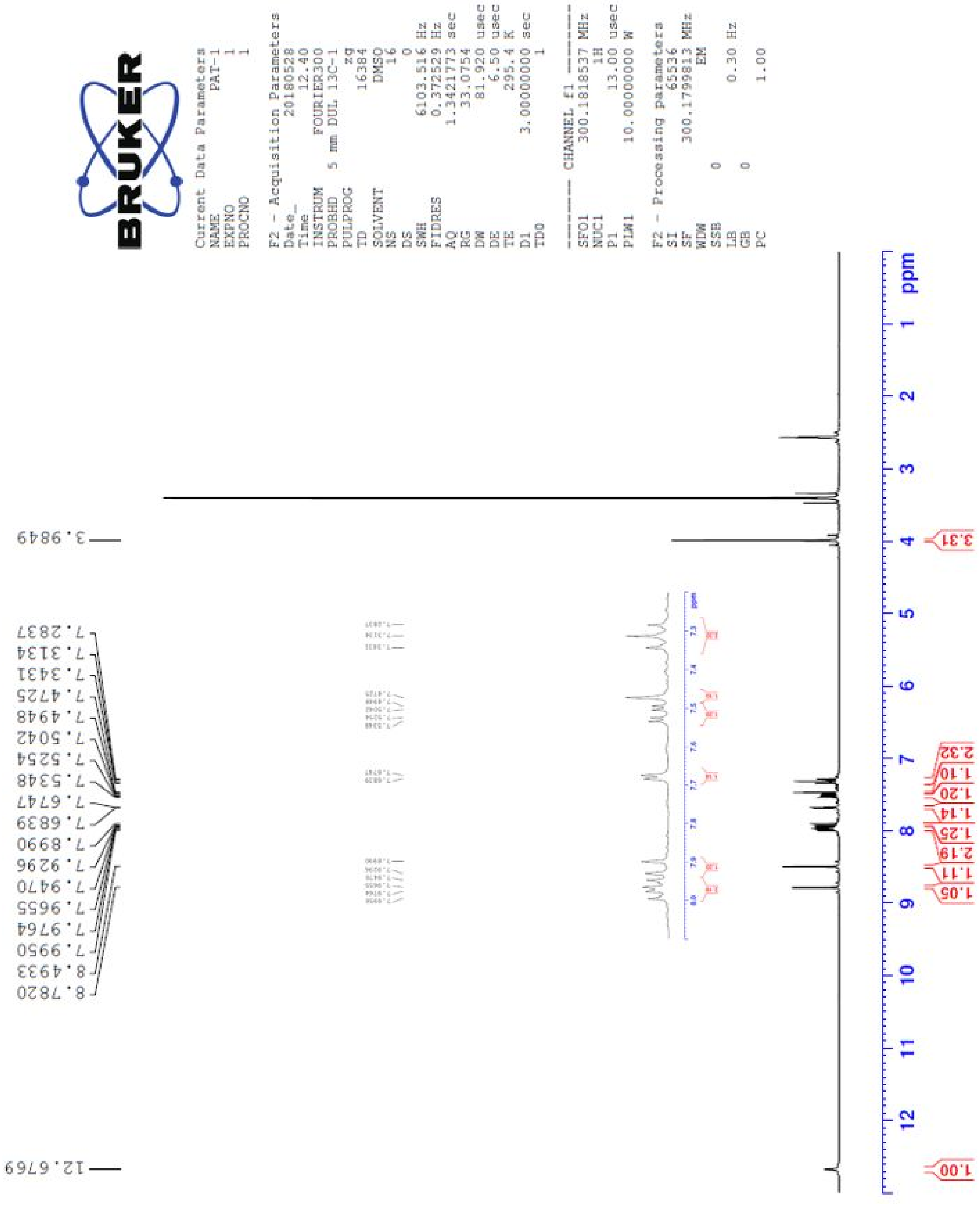

Figure S1. *Compound 4a ^{1}HNMR spectrum*

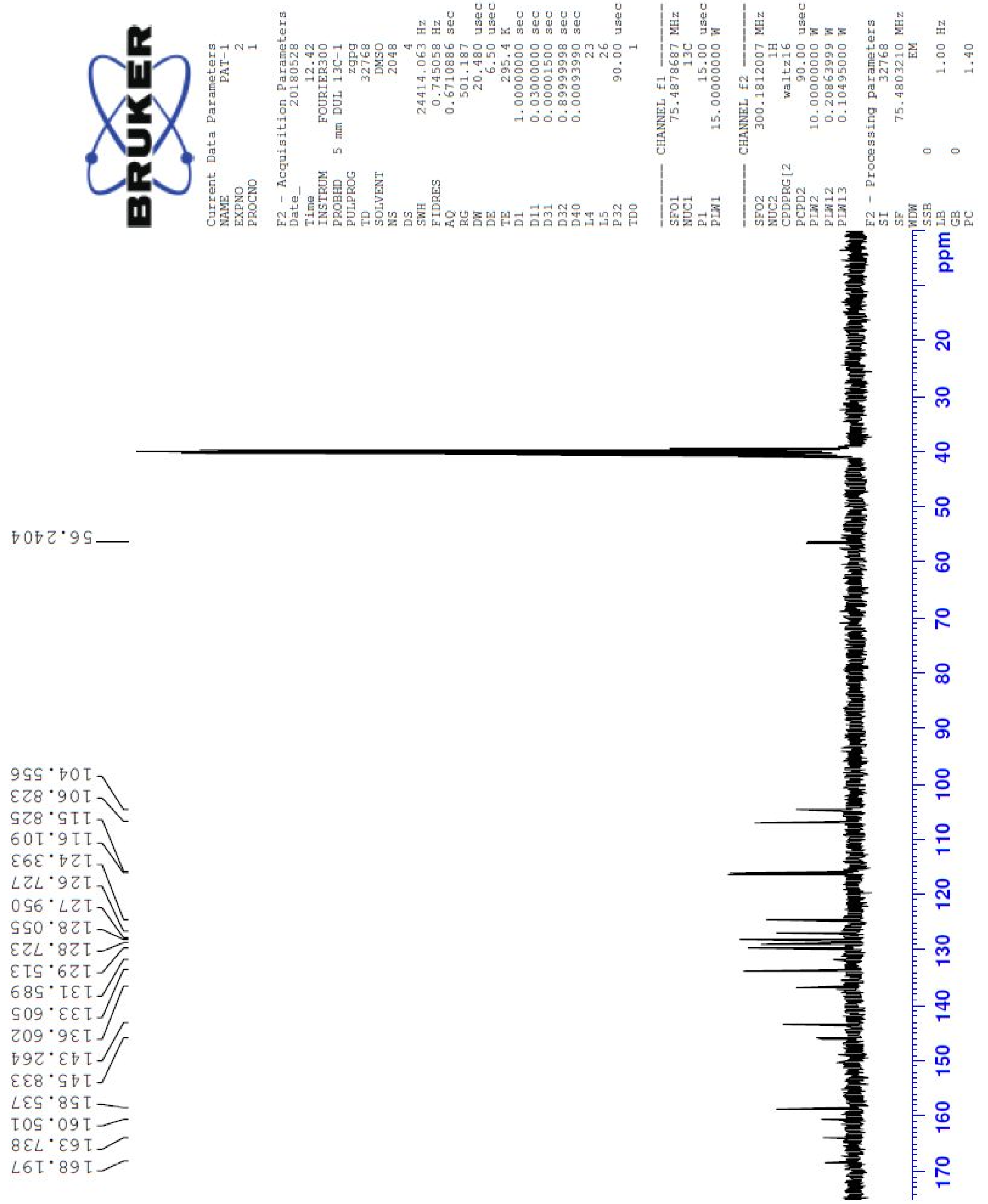

Figure S2. *Compound 4a ^{13}CNMR spectrum*

Data File: C:\LabSolutions\Data\Analiz\Lyuttas\PAT-1_20.lcd

Elmt	Val.	Min	Max	Elmt	Val.	Min	Max	Elmt	Val.	Min	Max	Elmt	Val.	Min	Max	Use Adduct
H	1	0	31	O	2	1	2	S	2	0	1	Ru	2	0	0	H
C	4	18	25	F	1	0	1	Cl	1	1	2	Pd	2	0	0	
N	3	4	5	P	3	0	0	Br	1	0	0	I	3	0	0	

Error Margin (ppm): 10
HC Ratio: unlimited
Max Isotopes: 3
MSn Iso RI (%): 10.00

DBE Range: 8.0 - 17.0
Apply N Rule: yes
Isotope RI (%): 1.00
MSn Logic Mode: AND

Electron Ions: both
Use MSn Info: yes
Isotope Res: 9000
Max Results: 500

Event#: 1 MS(E+) Ret. Time : 9.893 -> 10.133 Scan# : 1485 -> 1521

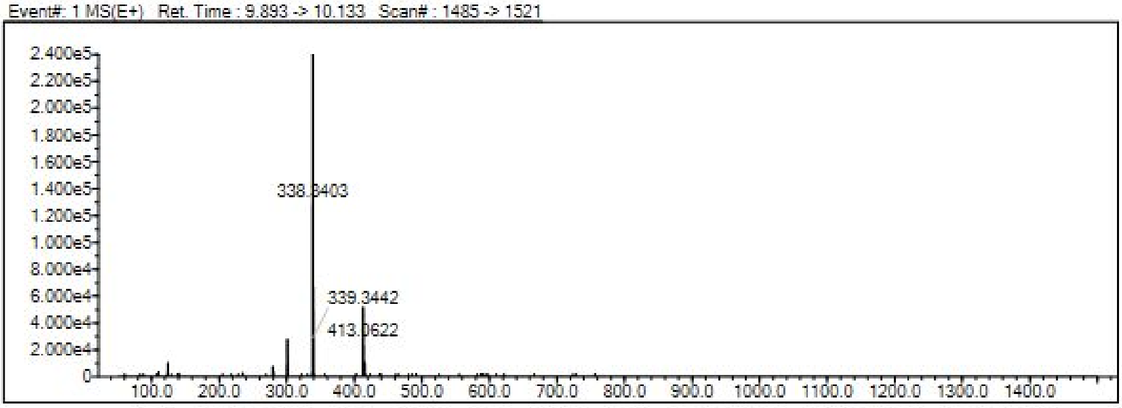

Measured region for 413.0622 m/z

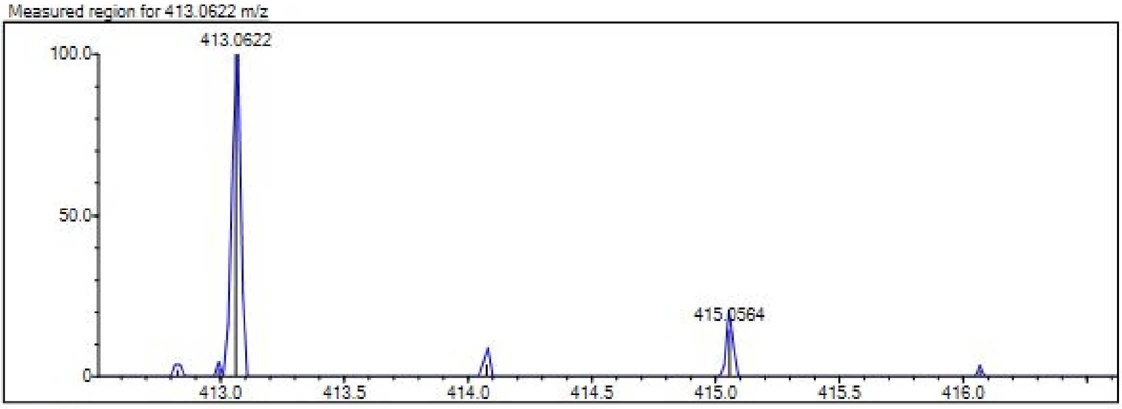

C20 H14 N4 O F S Cl [M+H]+ : Predicted region for 413.0634 m/z

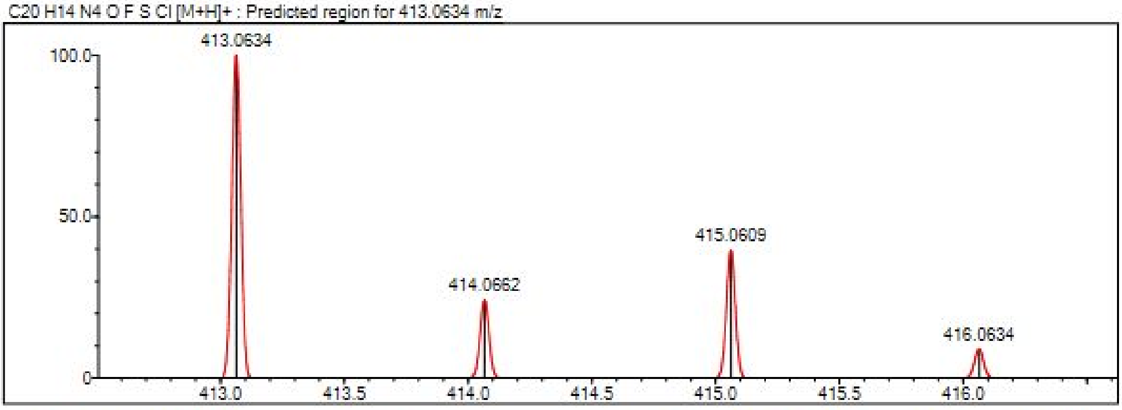

Rank	Score	Formula (M)	Ion	Meas. m/z	Pred. m/z	Df. (mDa)	Df. (ppm)	Iso	DBE
1	31.01	C20 H14 N4 O F S Cl	[M+H]+	413.0622	413.0634	-1.2	-2.91	32.56	15.0

Figure S3. *Compound 4a HRMS spectrum*

1.2. 2-{2-[(2-Chloro-6-methoxyquinolin-3-yl)methylene]hydrazinyl}-4-(4-chlorophenyl)thiazole (4b)

M.p: 195-197 °C. Appearance: Yellow powder. Yield: 82%.

^{1}H-NMR (300 MHz, DMSO-*d6*): δ: 3.92 (3H, s, O-CH_3), 7.44 (H, d, *J*=2.76 Hz, thiazole H_5), 7.48 (2H, d, *J*= 8.49 Hz, phenyl $H_{3,5}$), 7.50 (H, d, *J*=2.88 Hz, quinoline H_7), 7.62 (H, d, *J*=2.73 Hz, quinoline H_5), 7.84 (H, d, *J*= 9.32 Hz, quinoline H_8), 7.89 (2H, d, *J*= 8.59 Hz, phenyl $H_{2,6}$), 8.43 (H, s, -N=C-H), 8.72 (H, s, quinoline H_4), 12.63 (H, brs, =N-N-H).

^{13}C-NMR (75 MHz, DMSO-*d6*): δ: 56.21 (C, s, O-CH_3), 105.60 (C, s, thiazole C_5), 106.82 (C, s, quinoline C_5), 124.41 (C, s, quinoline C_7), 126.69 (2C, s, phenyl $C_{2,6}$), 127.72 (C, s, quinoline C_3), 128.72 (C, s, quinoline C_{4a}), 129.14 (C, s, quinoline C_8), 129.51 (2C, s, phenyl $C_{3,5}$), 132.50 (C, s, phenyl C_1), 133.62 (C, s, phenyl C_4), and 133.83 (C, s, quinoline C_4), 136.71 (C, s, quinoline C_2), 143.27 (C, s, N=C), 145.83 (C, s, quinoline C_{8a}), 158.53 (2C, s, quinoline C_6, thiazole C_4), 168.24 (C, s, thiazole C_2).

HRMS (-m/z): [M+H]$^+$: For $C_{20}H_{14}N_4OSCl_2$ calculated: 429.0338, found: 429.0337

InChI=1S/C20H14Cl2N4OS/c1-27-16-6-7-17-13(9-16)8-14(19(22)24-17)10-23-26-20-25-18(11-28-20)12-2-4-15(21)5-3-12/h2-11H,1H3,(H,25,26)/b23-10+

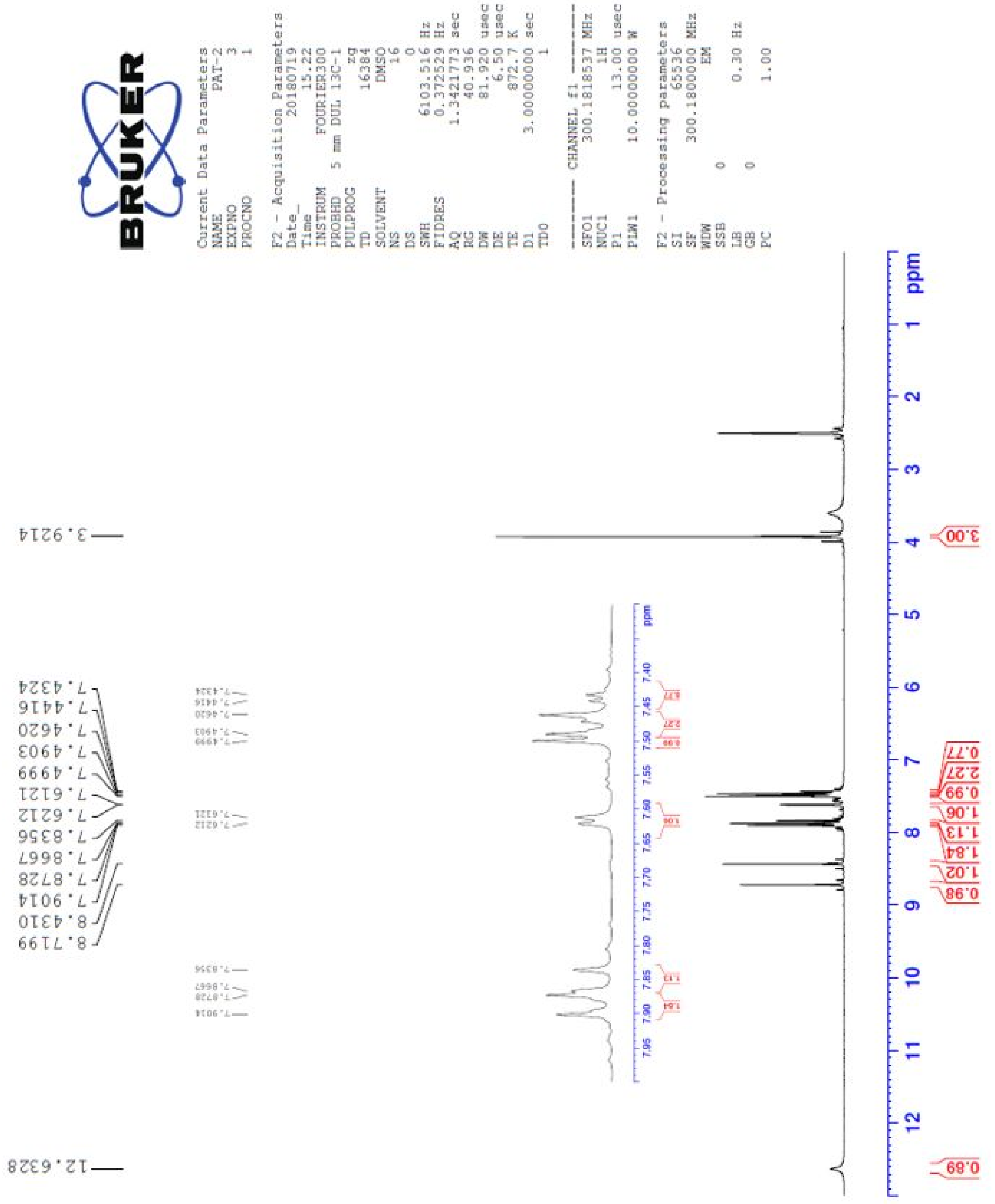

Figure S4. *Compound 4b ^{1}HNMR spectrum*

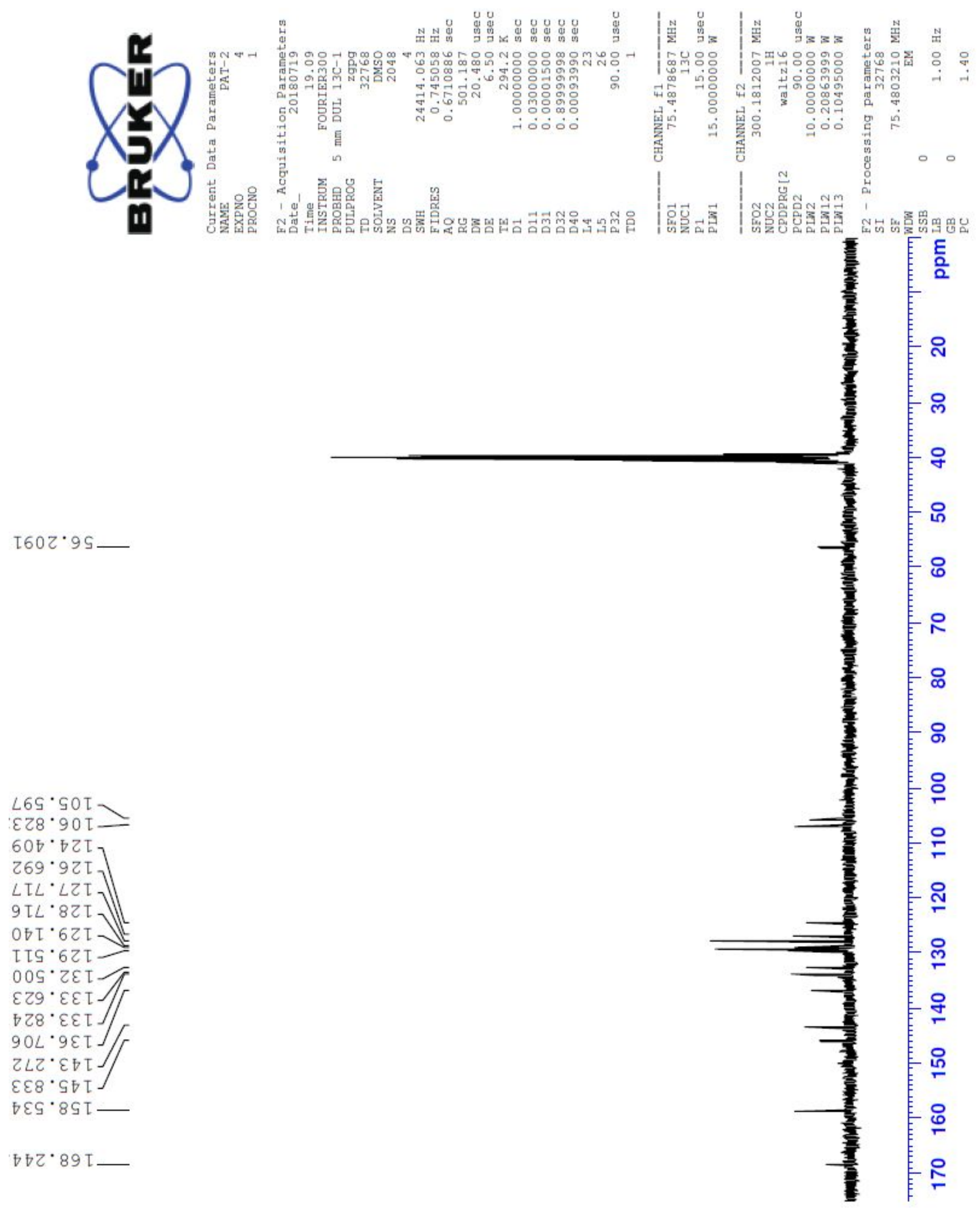

Figure S5. *Compound 4b ^{13}CNMR spectrum*

Formula Predictor Report - PAT-3_22.lcd

Page 1 of 1

Data File: C:\LabSolutions\Data\Analiz\Lyuttas\PAT-3_22.lcd

Elmt	Val.	Min	Max	Elmt	Val.	Min	Max	Elmt	Val.	Min	Max	Elmt	Val.	Min	Max	Use Adduct
H	1	0	31	O	2	1	2	S	2	0	1	Ru	2	0	0	H
C	4	18	25	F	1	0	0	Cl	1	1	2	Pd	2	0	0	
N	3	4	5	P	3	0	0	Br	1	0	0	I	3	0	0	

Error Margin (ppm): 10
HC Ratio: unlimited
Max Isotopes: 3
MSn Iso RI (%): 10.00

DBE Range: 8.0 - 17.0
Apply N Rule: yes
Isotope RI (%): 1.00
MSn Logic Mode: AND

Electron Ions: both
Use MSn Info: yes
Isotope Res: 9000
Max Results: 500

Event#: 1 MS(E+) Ret. Time : 8.840 -> 9.200 Scan# : 1327 -> 1381

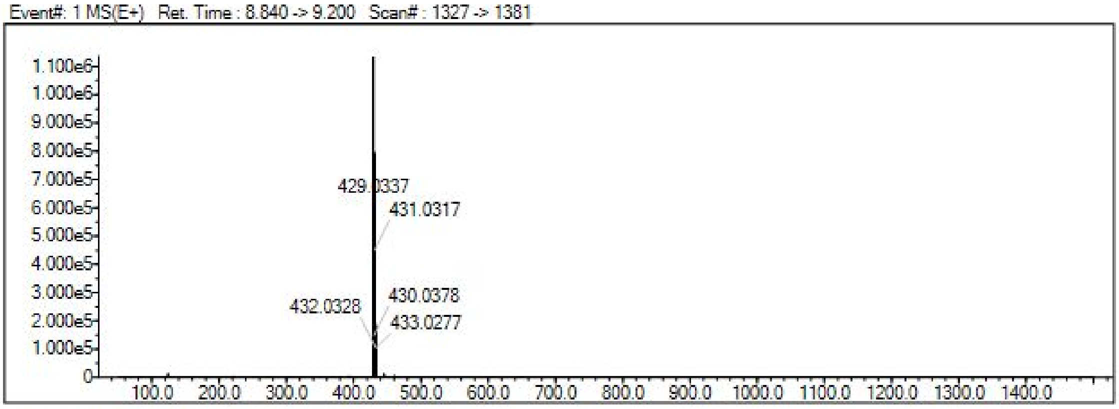

Measured region for 429.0337 m/z

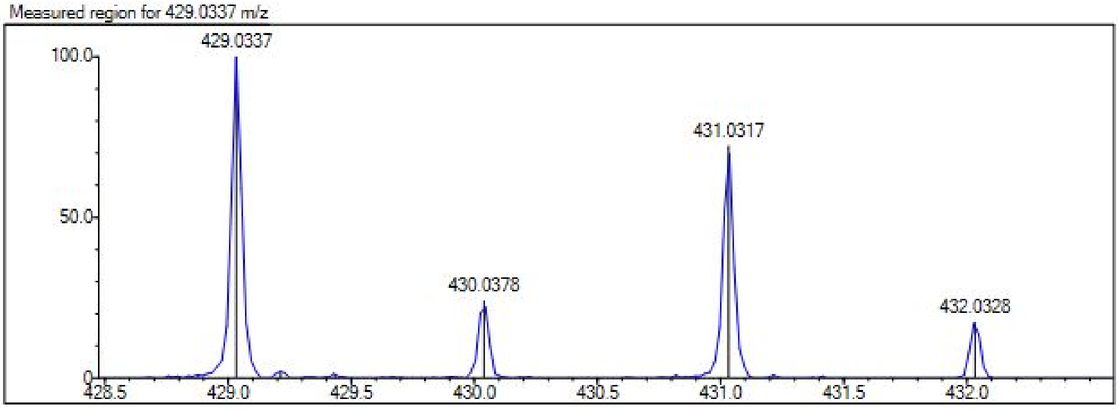

C20 H14 N4 O S Cl2 [M+H]+ : Predicted region for 429.0338 m/z

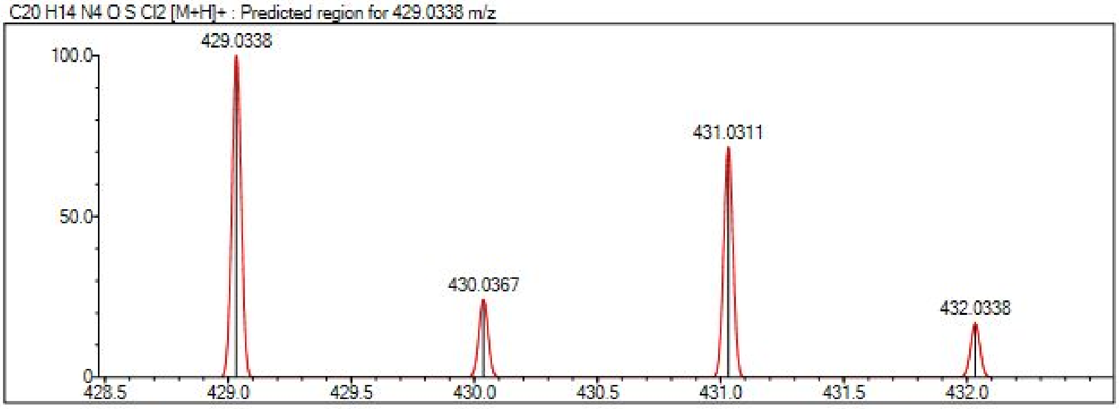

Rank	Score	Formula (M)	Ion	Meas. m/z	Pred. m/z	Df. (mDa)	Df. (ppm)	Iso	DBE
1	91.46	C20 H14 N4 O S Cl2	[M+H]+	429.0337	429.0338	-0.1	-0.23	91.46	15.0

Figure S6. *Compound 4b HRMS spectrum*

1.3. 2-{2-[(2-Chloro-6-methoxyquinolin-3-yl)methylene]hydrazinyl}-4-(4-nitrophenyl)thiazole (4c)

M.p: 245-253 °C. Appearance: Dark yellow powder. Yield: %85.

^{1}H-NMR (300 MHz, DMSO-*d6*): δ: 3.92 (3H, s, O-CH_3), 7.45 (H, dd, J_1=2.82 Hz, J_2=9.18 Hz, quinoline H_7), 7.60 (H, d, *J*=2.76 Hz, quinoline H_5), 7.81 (H, s, thiazole H_5), 7.84 (H, d, *J*=9.25 Hz, quinoline H_8), 8.11 (2H, d, *J*=8.98 Hz, phenyl $H_{3,5}$), 8.27 (2H, d, *J*= 9.00 Hz, phenyl $H_{2,6}$), 8.43 (H, s, -N=C-H), 8.71 (H, s, quinoline H_4), 12.71 (H, brs, =N-N-H).

^{13}C-NMR (75 MHz, DMSO-*d6*): δ: 56.23 (C, s, O-CH_3), 106.82 (C, s, quinoline C_5), 109.65 (C, s, thiazole C_5), 124.43 (C, s, quinoline C_7), 124.59 (2C, s, phenyl $C_{3,5}$), 126.59 (C, s, quinoline C_3), 126.82 (2C, s, phenyl $C_{2,6}$), 128.68 (C, s, quinoline C_{4a}), 129.50 (C, s, quinoline C_8), 133.70 (C, s, phenyl C_1), 137.05 (C, s, quinoline C_4), 140.93 (C, s, thiazole C_4), 143.30 (C, s, N=C), 145.83 (C, s, quinoline C_{8a}), 146.71 (C, s, phenyl C_4) , 149.10 (C, s, quinoline C_2), 158.53 (C, s, quinoline C_6), 168.55 (C, s, thiazole C_2).

HRMS (-m/z): [M+H]$^+$: For $C_{20}H_{14}N_5O_3SCl$ calculated: 440.0579, found: 440.0577

InChI=1S/C20H14ClN5O3S/c1-29-16-6-7-17-13(9-16)8-14(19(21)23-17)10-22-25-20-24-18(11-30-20)12-2-4-15(5-3-12)26(27)28/h2-11H,1H3,(H,24,25)/b22-10+

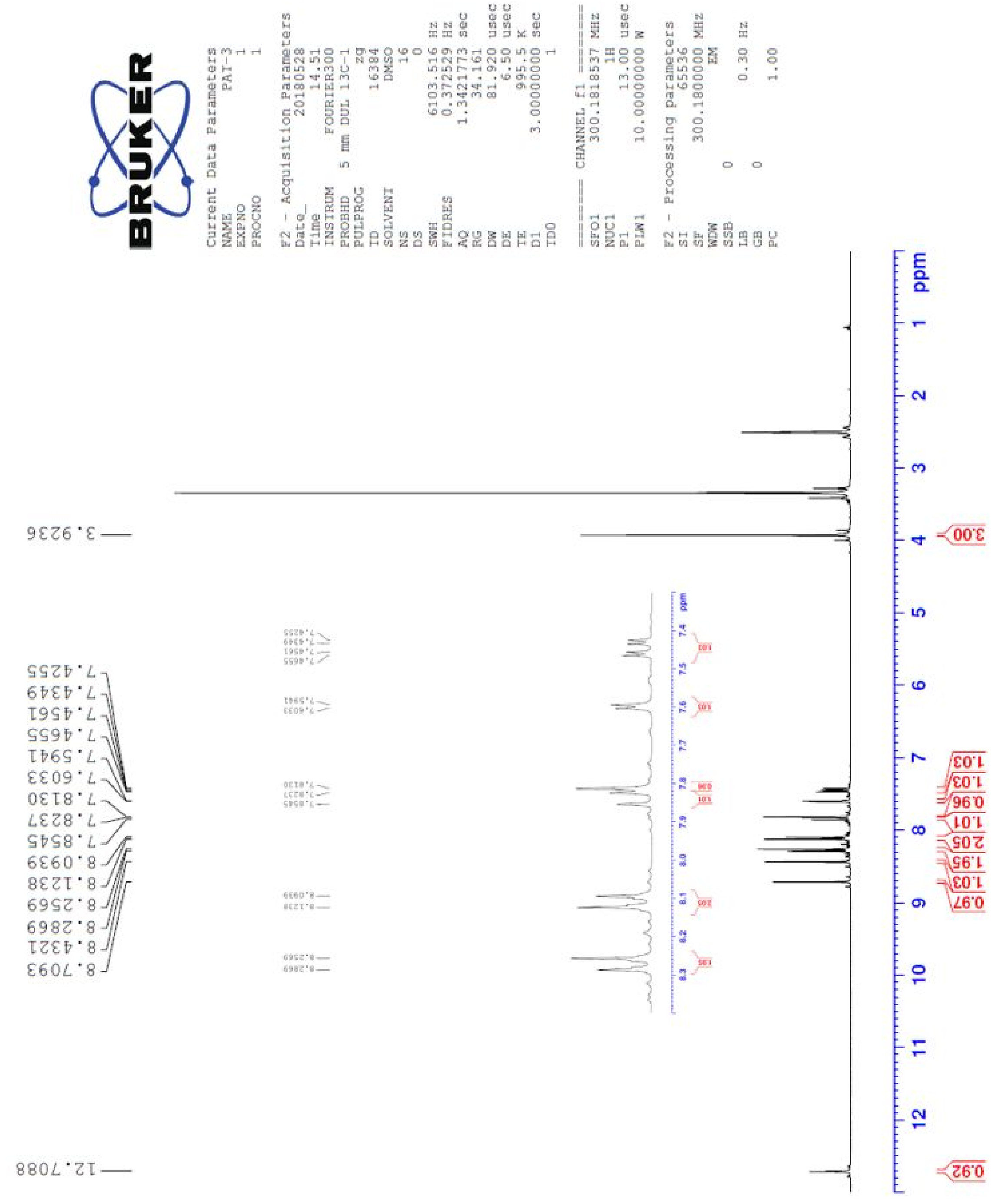

Figure S7. *Compound 4c ¹HNMR spectrum*

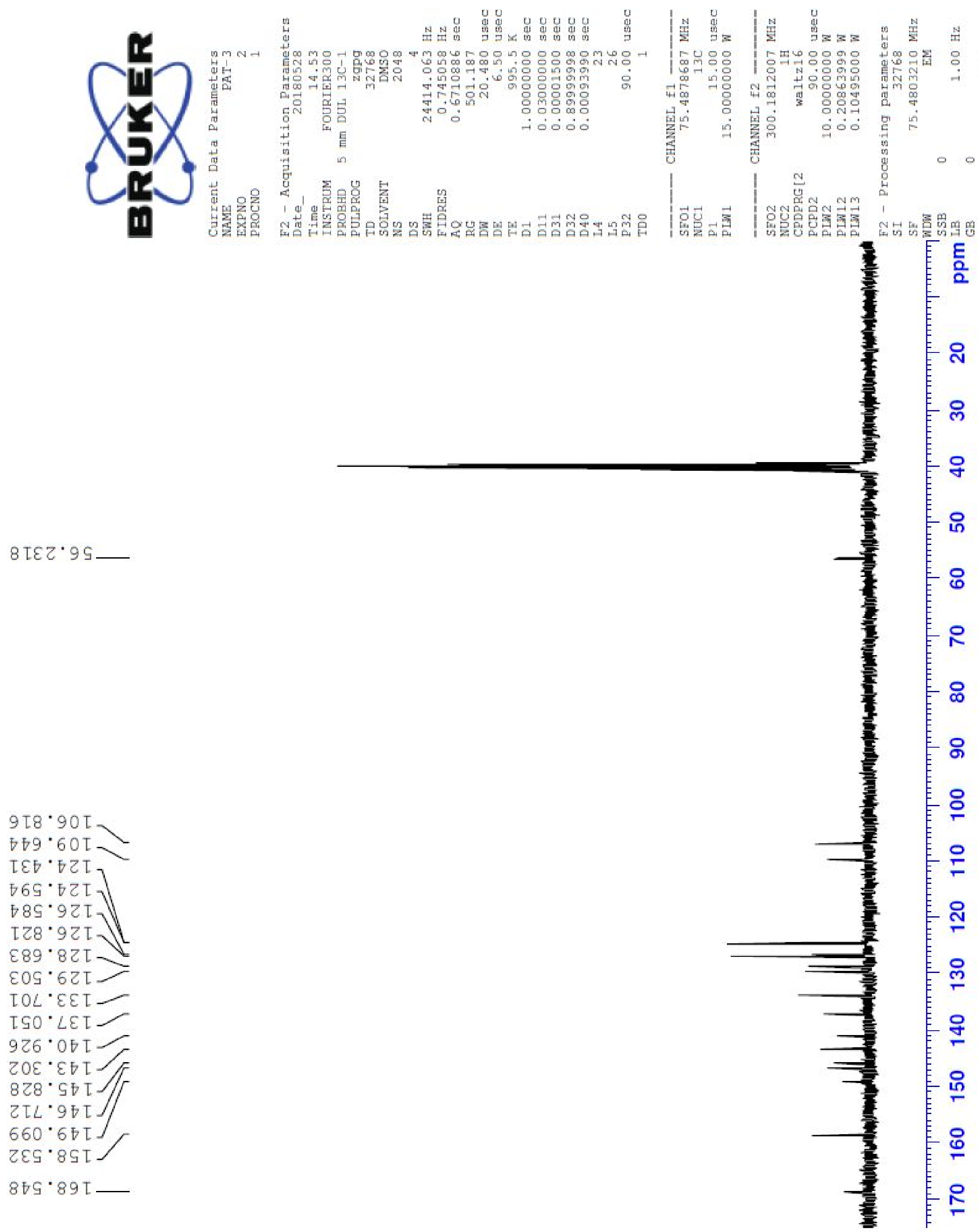

Figure S8. *Compound 4c ^{13}CNMR spectrum*

Data File: C:\LabSolutions\Data\Analiz\Lyuttas\PAT-3_22.lcd

Elmt	Val.	Min	Max	Elmt	Val.	Min	Max	Elmt	Val.	Min	Max	Elmt	Val.	Min	Max	Use Adduct
H	1	0	31	O	2	1	3	S	2	0	1	Ru	2	0	0	H
C	4	18	25	F	1	0	0	Cl	1	1	2	Pd	2	0	0	
N	3	4	5	P	3	0	0	Br	1	0	0	I	3	0	0	

Error Margin (ppm):	10	DBE Range:	8.0 - 17.0	Electron Ions:	both
HC Ratio:	unlimited	Apply N Rule:	yes	Use MSn Info:	yes
Max Isotopes:	3	Isotope RI (%):	1.00	Isotope Res:	9000
MSn Iso RI (%):	10.00	MSn Logic Mode:	AND	Max Results:	500

Event#: 1 MS(E+) Ret. Time : 9.813 -> 10.160 Scan# : 1473 -> 1525

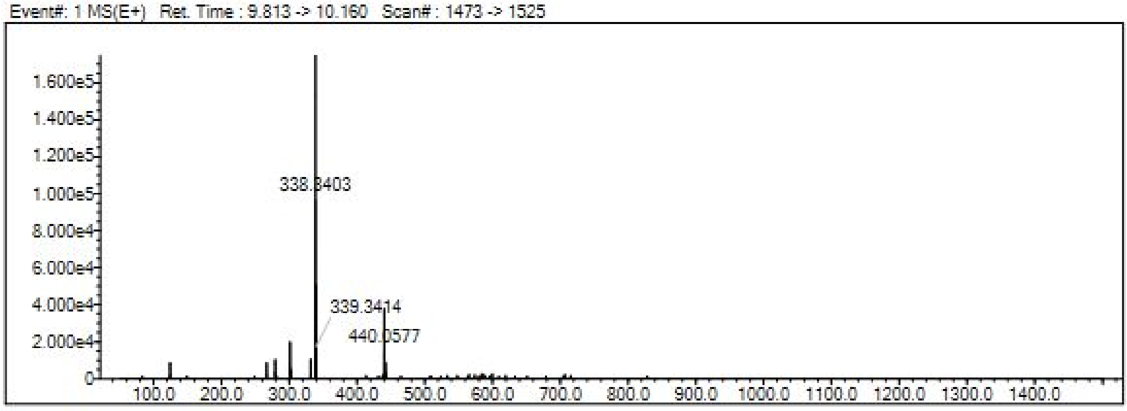

Measured region for 440.0577 m/z

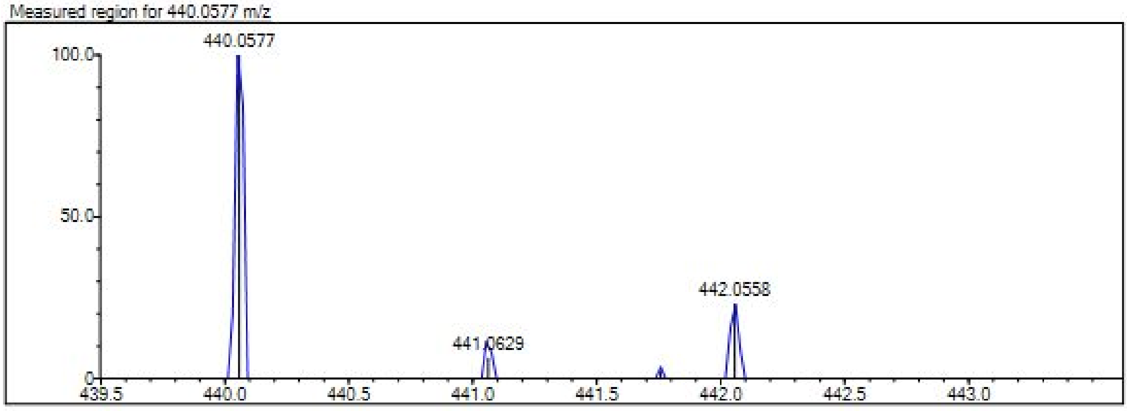

C20 H14 N5 O3 S Cl [M+H]+ : Predicted region for 440.0579 m/z

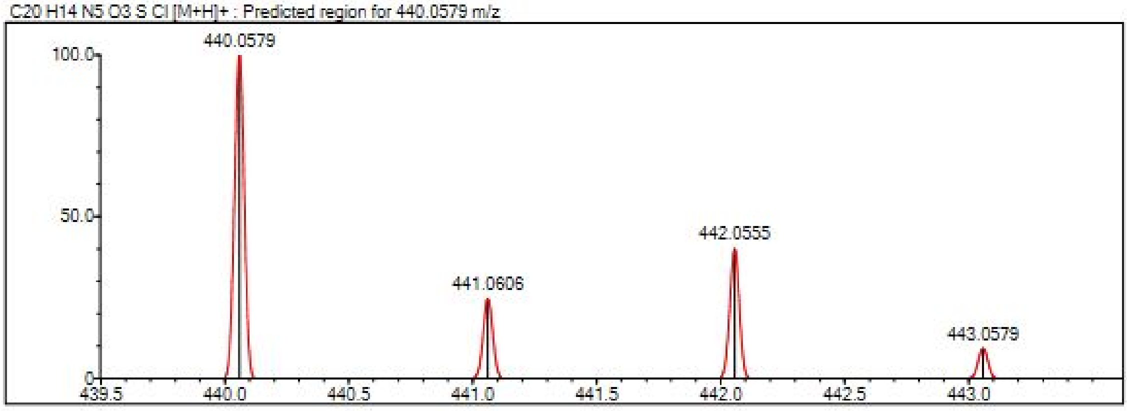

Rank	Score	Formula (M)	Ion	Meas. m/z	Pred. m/z	Df. (mDa)	Df. (ppm)	Iso	DBE
1	52.34	C20 H14 N5 O3 S Cl	[M+H]+	440.0577	440.0579	-0.2	-0.45	52.34	16.0

Figure S9. *Compound 4c HRMS spectrum*

1.4. 2-{2-[(2-Chloro-6-methoxyquinolin-3-yl)methylene]hydrazinyl}-4-phenylthiazole (4d)

M.p: 220-222 °C. Appearance: light yellow powder. Yield: %73.

^{1}H-NMR (300 MHz, DMSO-*d6*): δ: 3.92 (3H, s, O-CH_3), 7.31 (H, t, *J*=7.28, phenyl H_4), 7.41 (2H, d, J= 7.92 Hz, phenyl $H_{3,5}$), 7.43 (H, s, thiazole H_5), 7.47 (H, dd, J_1=2.79 Hz, J_2= 6.63 Hz, quinoline H_7), 7.61 (H, d, *J*= 2.76 Hz, quinoline H_5), 7.84-7.89 (3H, m, phenyl $H_{2,6}$, quinoline H_8), 8.43 (H, s, -N=C-H), 8.72 (H, s, quinoline H_4), 12.62 (H, brs, =N-N-H).

^{13}C-NMR (75 MHz, DMSO-*d6*): δ: 56.24 (O-CH_3), 104.78 (C, s, thiazole C_5), 106.82 (C, s, quinoline C_5), 124.37 (C, s, quinoline C_7), 126.01 (C, s, phenyl $C_{2,6}$), 126.75 (C, s, quinoline C_3), 128.12 (C, s, quinoline C_{4a}), 128.73 (C, s, quinoline C_8), 129.13 (C, s, phenyl $C_{3,5}$), 129.51 (C, s, phenyl C_4), 133.57 (C, s, phenyl C_1), 134.50 (C, s, quinoline C_4), 143.52 (C, s, N=C), 145.83 (C, s, quinoline C_{8a}), 151.17 (C, s, quinoline C_2), 158.53 (C, s, quinoline C_6, thiazole C_4), 168.11 (C, s, thiazole C_2).

HRMS (-m/z): [M+H]$^+$: For $C_{20}H_{15}N_4OSCl$ calculated: 395.0728 , found: 395.0745

InChI=1S/C20H15ClN4OS/c1-26-16-7-8-17-14(10-16)9-15(19(21)23-17)11-22-25-20-24-18(12-27-20)13-5-3-2-4-6-13/h2-12H,1H3,(H,24,25)/b22-11+

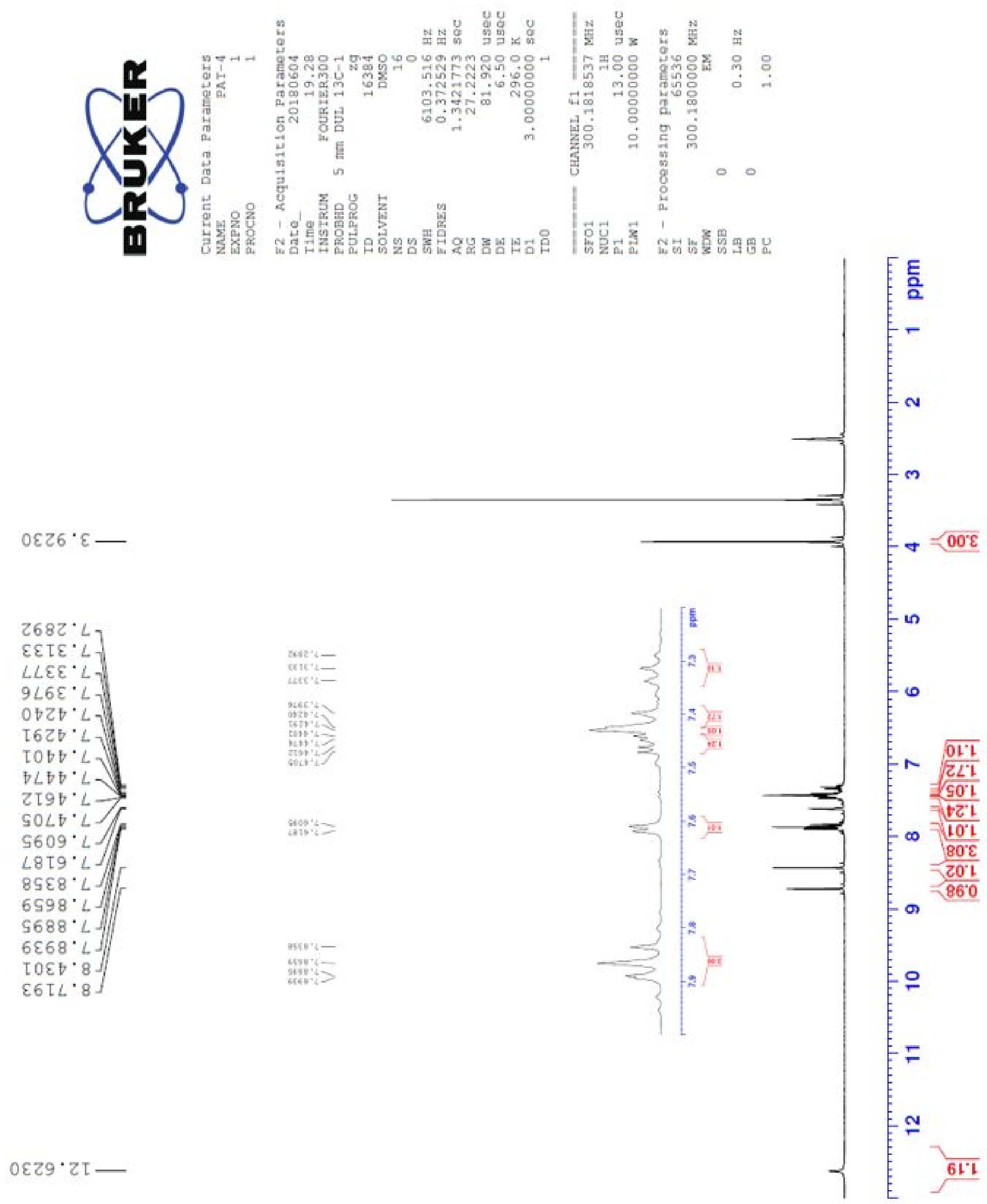

Figure S10. *Compound 4d ^{1}HNMR spectrum*

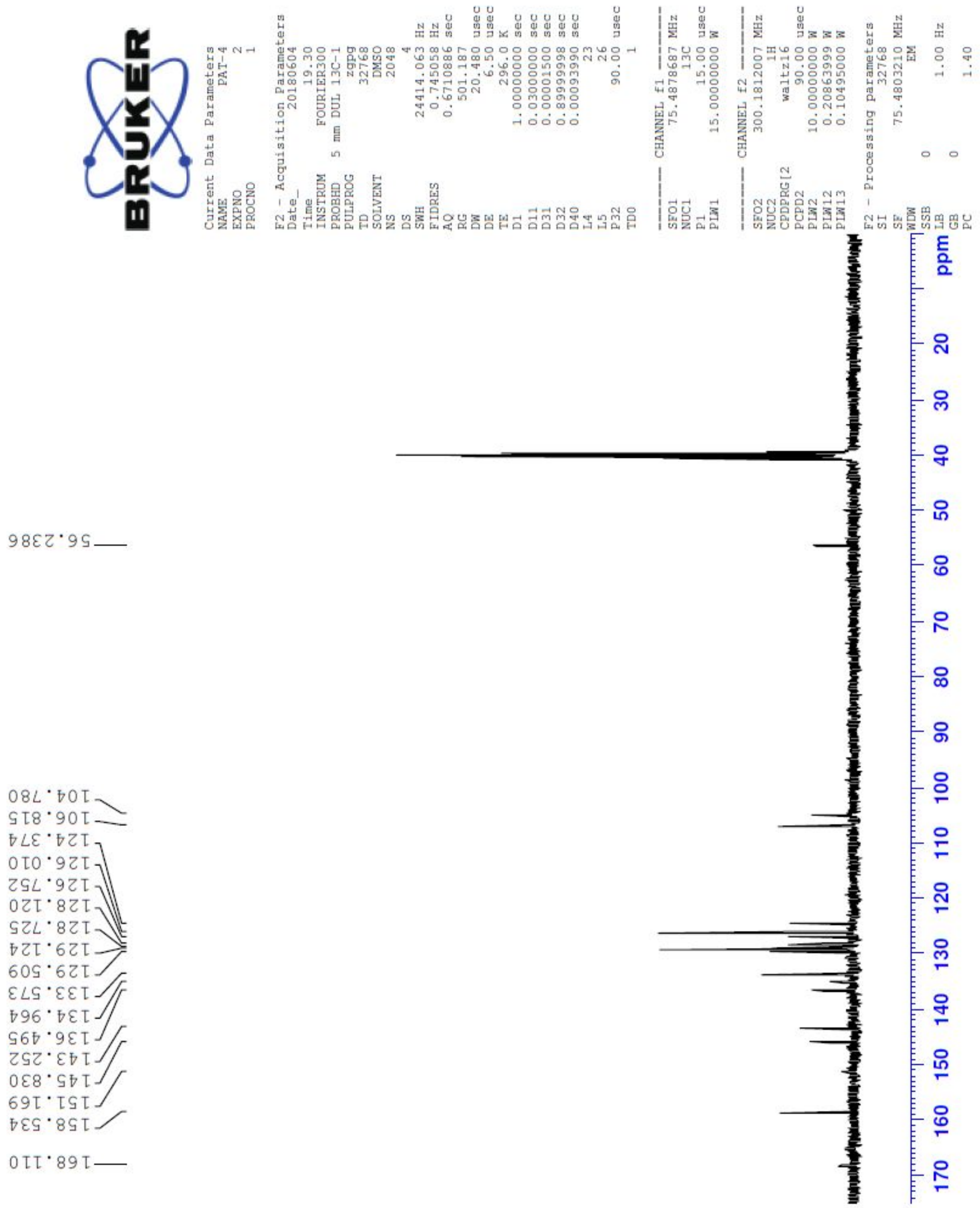

Figure S11. *Compound 4d* ^{13}*CNMR spectrum*

Data File: C:\LabSolutions\Data\Analiz\Lyuttas\PAT-5_18.lcd

Elmt	Val.	Min	Max	Elmt	Val.	Min	Max	Elmt	Val.	Min	Max	Elmt	Val.	Min	Max	Use Adduct
H	1	0	31	O	2	1	3	S	2	0	1	Ru	2	0	0	H
C	4	18	25	F	1	0	0	Cl	1	1	2	Pd	2	0	0	
N	3	4	5	P	3	0	0	Br	1	0	0	I	3	0	0	

Error Margin (ppm): 10
HC Ratio: unlimited
Max Isotopes: 3
MSn Iso RI (%): 10.00

DBE Range: 8.0 - 17.0
Apply N Rule: yes
Isotope RI (%): 1.00
MSn Logic Mode: AND

Electron Ions: both
Use MSn Info: yes
Isotope Res: 9000
Max Results: 500

Event#: 1 MS(E+) Ret. Time : 6.160 -> 6.733 Scan# : 925 -> 1011

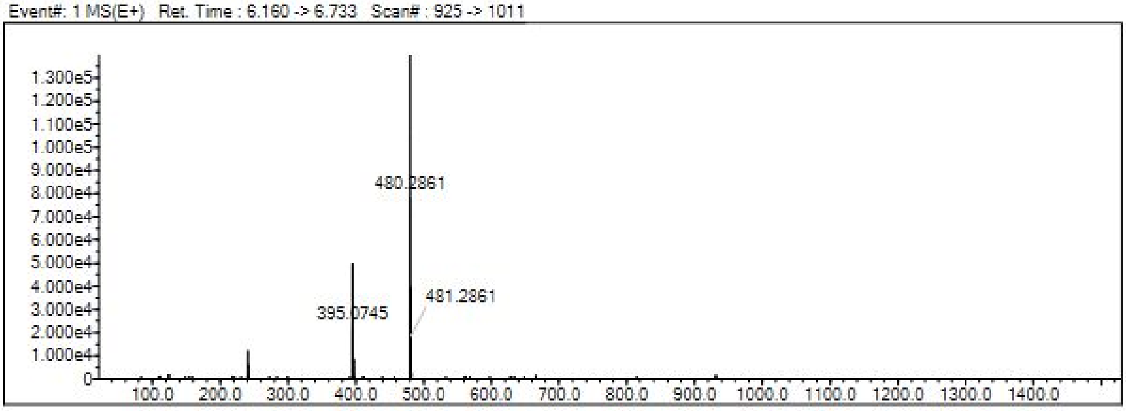

Measured region for 395.0745 m/z

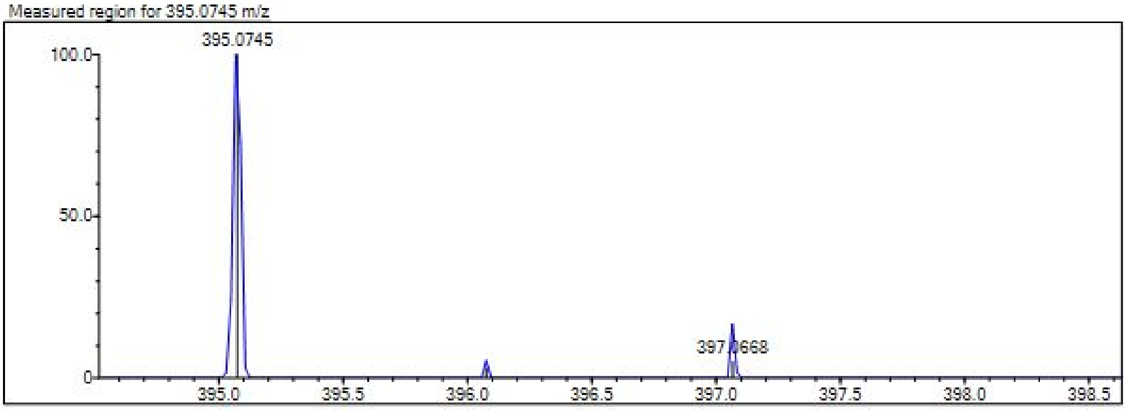

C20 H15 N4 O S Cl [M+H]+ : Predicted region for 395.0728 m/z

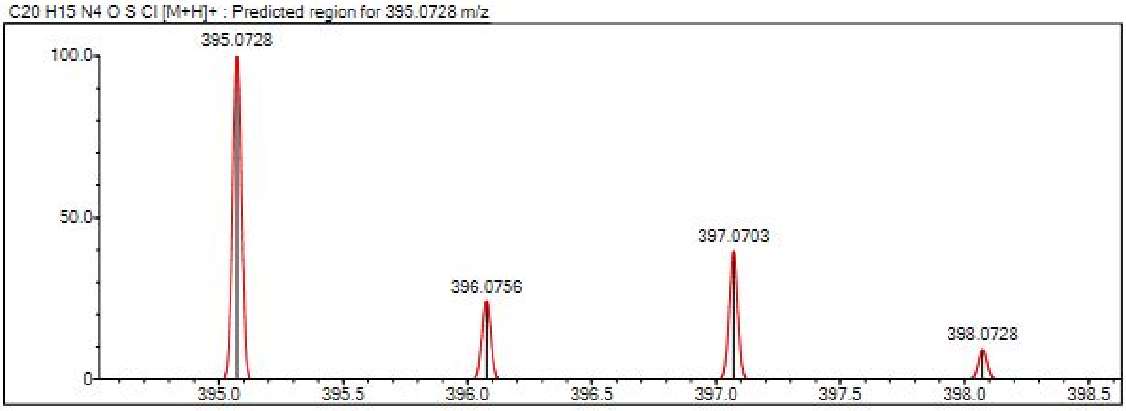

Rank	Score	Formula (M)	Ion	Meas. m/z	Pred. m/z	Df. (mDa)	Df. (ppm)	Iso	DBE
1	37.87	C20 H15 N4 O S Cl	[M+H]+	395.0745	395.0728	1.7	4.30	41.27	15.0

Figure S12. *Compound 4d HRMS spectrum*

1.5. 2-{2-[(2-Chloro-6-methoxyquinolin-3-yl)methylene]hydrazinyl}-4-(4-cyanophenyl)thiazole (4e)

M.p: 240-242 °C. Appearance: pale-yellow powder. Yield: %79.

^{1}H-NMR (300 MHz, DMSO-*d6*): δ: 3.91 (3H, s, O-CH_3), 7.43 (H, dd, J_1= 2.79 Hz, J_2= 9.19 Hz, quinoline H_7), 7.58 (H, d, *J*= 2.75 Hz, quinoline H_5), 7.72 (H, s, thiazole H_5), 7.81 (H, s, quinoline H_8), 7.86 (2H, d, *J*= 8.67 Hz, phenyl $H_{3,5}$), 8.03 (2H, d, *J*= 8.46 Hz, phenyl $H_{2,6}$), 8.41 (H, s, -N=C-H), 8.68 (H, s, quinoline H_4), 12.66 (H, brs, =N-N-H).

^{13}C-NMR (75 MHz, DMSO-*d6*): δ: 56.21 (C, s, O-CH_3), 106.78 (C, s, quinoline C_5),108.60 (C, s, thiazole C_5), 110.13 (C, s, phenyl C_4), 119.44 (C, s, cyano $\underline{C}$N), 124.38 (C, s, quinoline C_7), 126.59 (2C, s, phenyl $C_{2,6}$), 128.66 (C, s, quinoline C_3), 129.49 (C, s, quinoline C_{4a}), 133.16 (2C, s, phenyl $C_{3,5}$), 133.63 (C, s, quinoline C_4), 136.94 (C, s, phenyl C_1), 139.03 (C, s, quinoline C_8), 143.28 (C, s, N=C), 145.82 (C, s, quinoline C_{8a}), 149.42 (C, s, quinoline C_2), 158.51 (2C, s, quinoline C_6, thiazole C_4),), 168.43 (C, s, thiazole C_2).

HRMS (-m/z): [M+H]$^+$: For $C_{21}H_{14}N_5OSCl$ calculated: 420.0680, found: 420.0687

InChI=1S/C21H14ClN5OS/c1-28-17-6-7-18-15(9-17)8-16(20(22)25-18)11-24-27-21-26-19(12-29-21)14-4-2-13(10-23)3-5-14/h2-9,11-12H,1H3,(H,26,27)/b24-11+

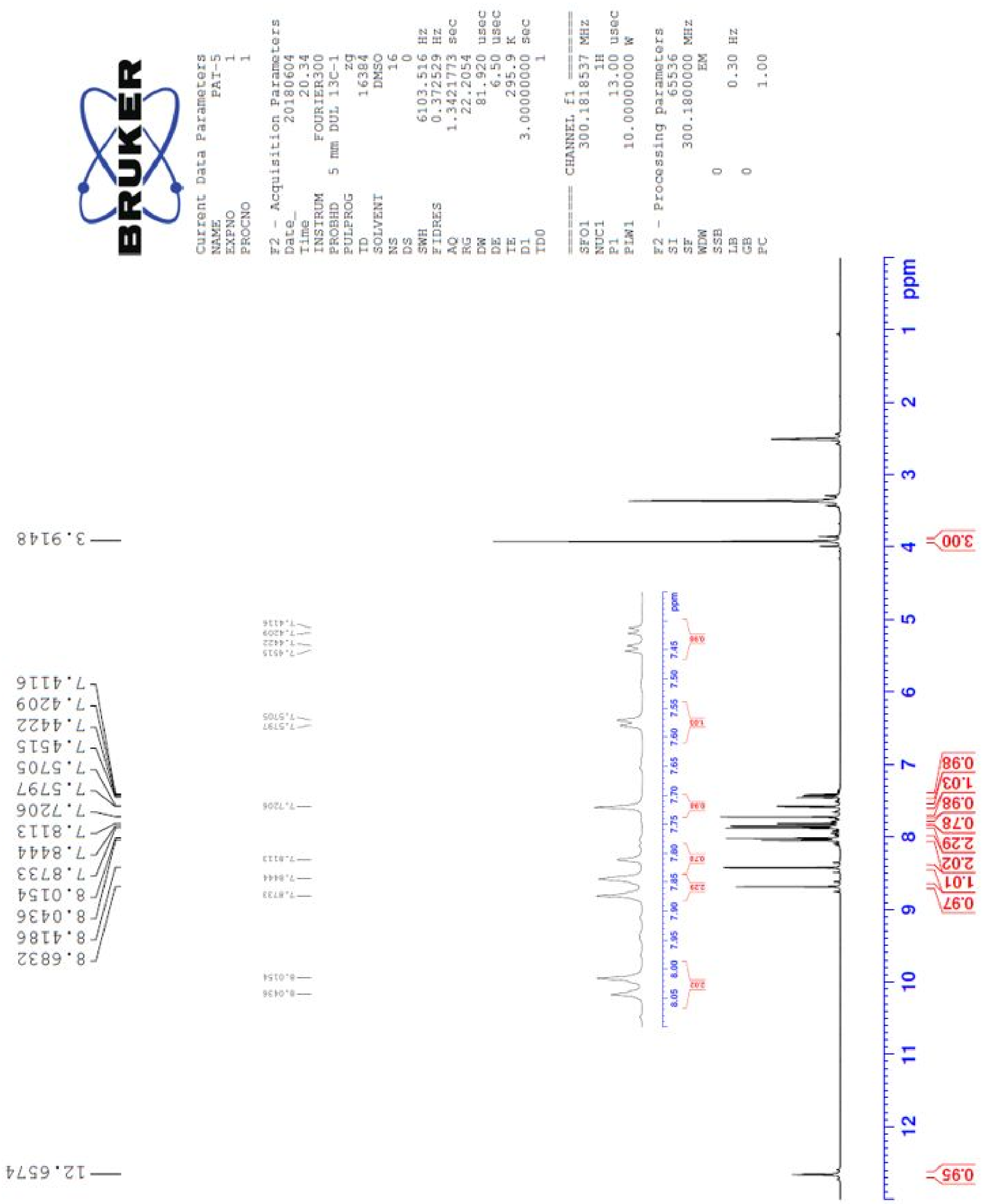

Figure S13. *Compound 4e ^{1}HNMR spectrum*

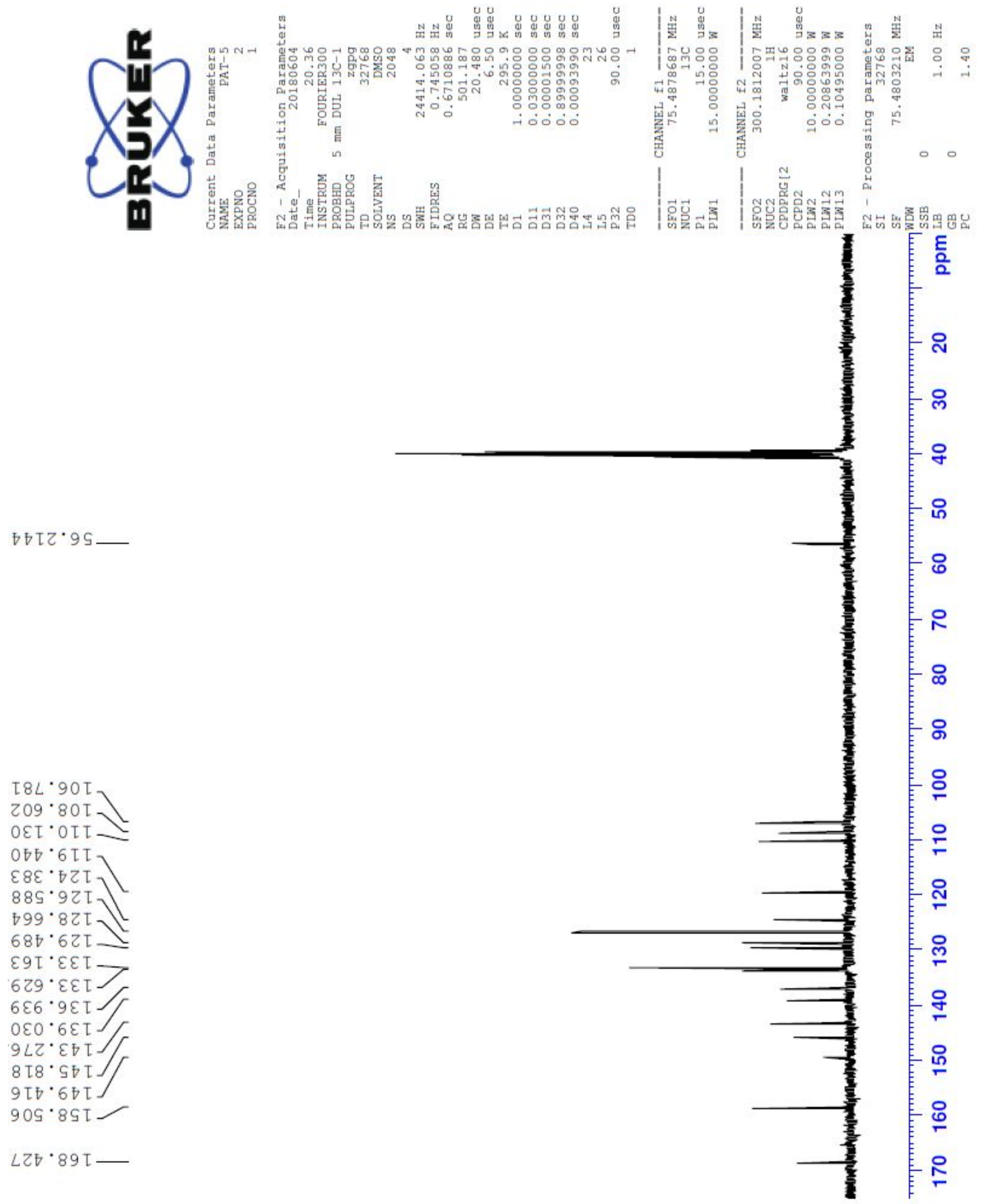

Figure S14. *Compound 4e* [13]*CNMR spectrum*

Data File: C:\LabSolutions\Data\Analiz\Lyuttas\PAT-5_18.lcd

Elmt	Val.	Min	Max	Elmt	Val.	Min	Max	Elmt	Val.	Min	Max	Elmt	Val.	Min	Max	Use Adduct
H	1	0	31	O	2	1	2	S	2	0	1	Ru	2	0	0	H
C	4	18	25	F	1	0	0	Cl	1	1	2	Pd	2	0	0	
N	3	4	5	P	3	0	0	Br	1	0	0	I	3	0	0	

Error Margin (ppm): 10
HC Ratio: unlimited
Max Isotopes: 3
MSn Iso RI (%): 10.00

DBE Range: 8.0 - 17.0
Apply N Rule: yes
Isotope RI (%): 1.00
MSn Logic Mode: AND

Electron Ions: both
Use MSn Info: yes
Isotope Res: 9000
Max Results: 500

Event#: 1 MS(E+) Ret. Time : 9.267 -> 9.373 Scan# : 1391 -> 1407

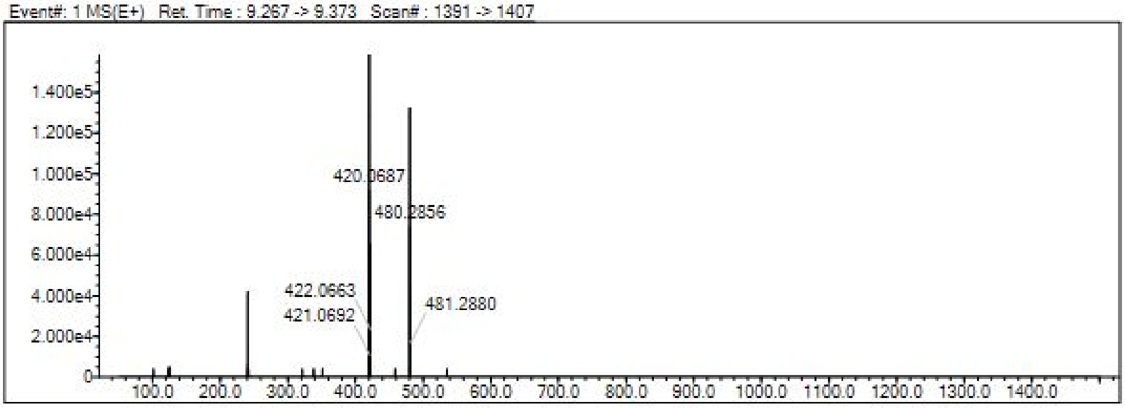

Measured region for 420.0687 m/z

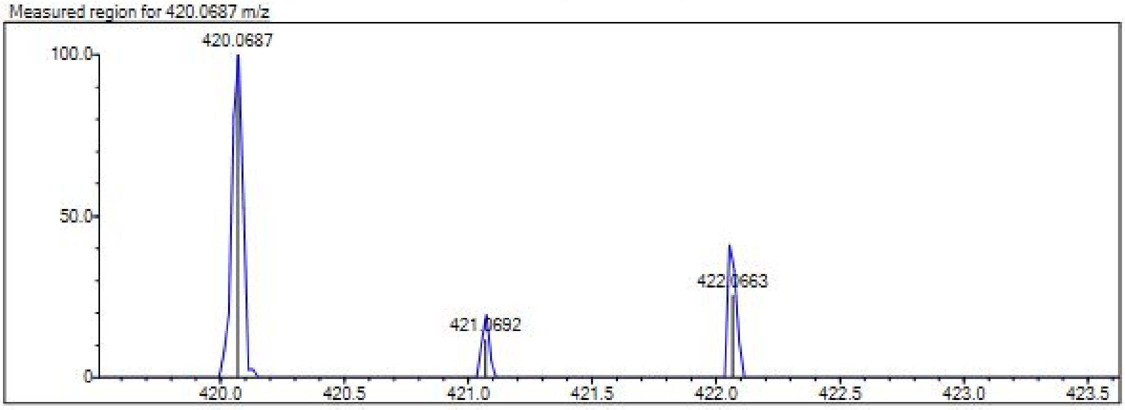

C21 H14 N5 O S Cl [M+H]+ : Predicted region for 420.0680 m/z

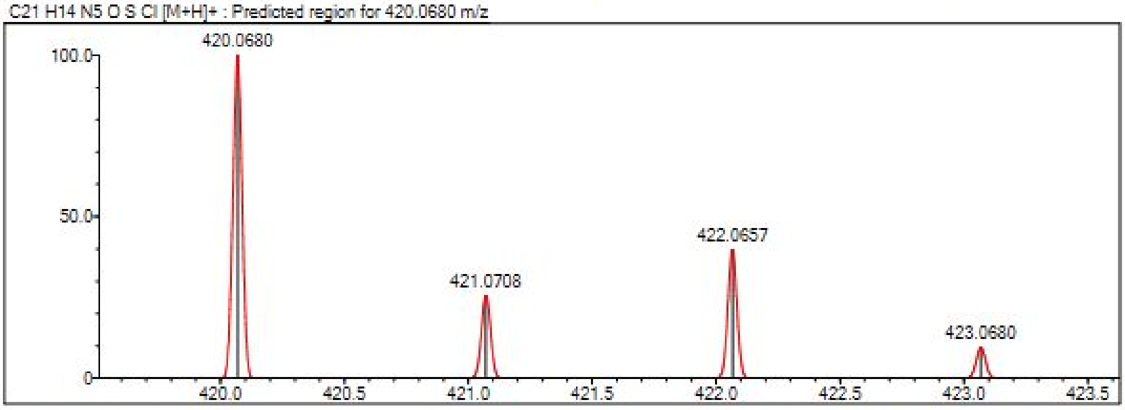

Rank	Score	Formula (M)	Ion	Meas. m/z	Pred. m/z	Df. (mDa)	Df. (ppm)	Iso	DBE
1	61.94	C21 H14 N5 O S Cl	[M+H]+	420.0687	420.0680	0.7	1.67	62.99	17.0

Figure S15. *Compound 4e HRMS spectrum*

1.6. 2-{2-[(2-Chloro-6-methoxyquinolin-3-yl)methylene]hydrazinyl}-4-(4-methylphenyl)thiazole (4f)

M.p: 194-196 °C. Appearance: Dark brown powder. Yield: %70.

^{1}H-NMR (300 MHz, DMSO-*d6*): δ: 2.31 (3H, s, CH_3), 3.91 (3H, s, O-CH_3), 7.22 (2H, d, *J*= 8.04 Hz, phenyl $H_{3,5}$), 7.34 (H, s, thiazole H_5), 7.44 (H, dd, J_1= 2.81 Hz, J_2= 9.16 Hz, quinoline H_7), 7.59 (H, d, *J*= 2.77 Hz, quinoline H_5), 7.75 (2H, d, *J*= 8.10 Hz, phenyl $H_{2,6}$), 7.84 (H, d, *J*= 9.19 Hz, quinoline H_8), 8.43 (H, s, -N=C-H), 8.70 (H, s, quinoline H_4).

^{13}C-NMR (75 MHz, DMSO-*d6*): δ: 21.27 (C, s, phenyl CH_3), 56.23 (C, s, O-CH_3), 103.85 (C, s, thiazole C_5), 106.80 (C, s, quinoline C_5), 124.36 (C, s, quinoline C_7), 125.95 (2C, s, phenyl $C_{2,6}$), 126.76 (C, s, quinoline C_3), 128.72 (C, s, quinoline C_{4a}), 129.50 (C, s, quinoline C_8), 129.68 (2C, s, phenyl $C_{3,5}$), 132.33 (C, s, phenyl C_1), 133.53 (C, s, phenyl C_4), 136.38 (C, s, quinoline C_4), 137.39 (2C, s, quinoline C_2, thiazole C_4), 143.24 (C, s, N=C), 145.82 (C, s, quinoline C_{8a}), 158.52 (C, s, quinoline C_6), 167.99 (C, s, thiazole C_2).

HRMS (-m/z): [M+H]$^+$: For $C_{21}H_{17}N_4OSCl$ calculated: 409.0884, found: 409.0868

InChI=1S/C21H17ClN4OS/c1-13-3-5-14(6-4-13)19-12-28-21(25-19)26-23-11-16-9-15-10-17(27-2)7-8-18(15)24-20(16)22/h3-12H,1-2H3,(H,25,26)/b23-11+

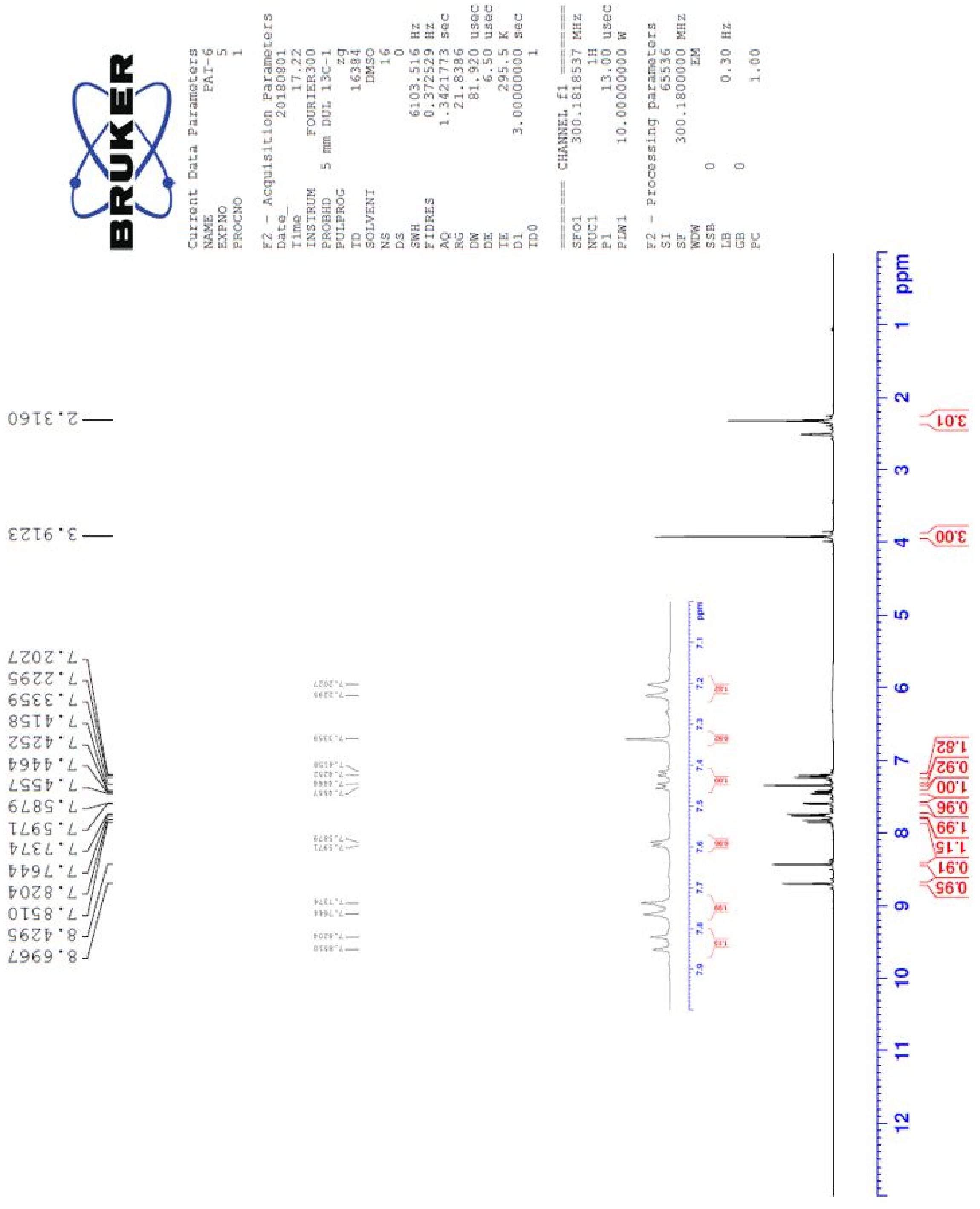

Figure S16. *Compound 4f* ^{1}*HNMR spectrum*

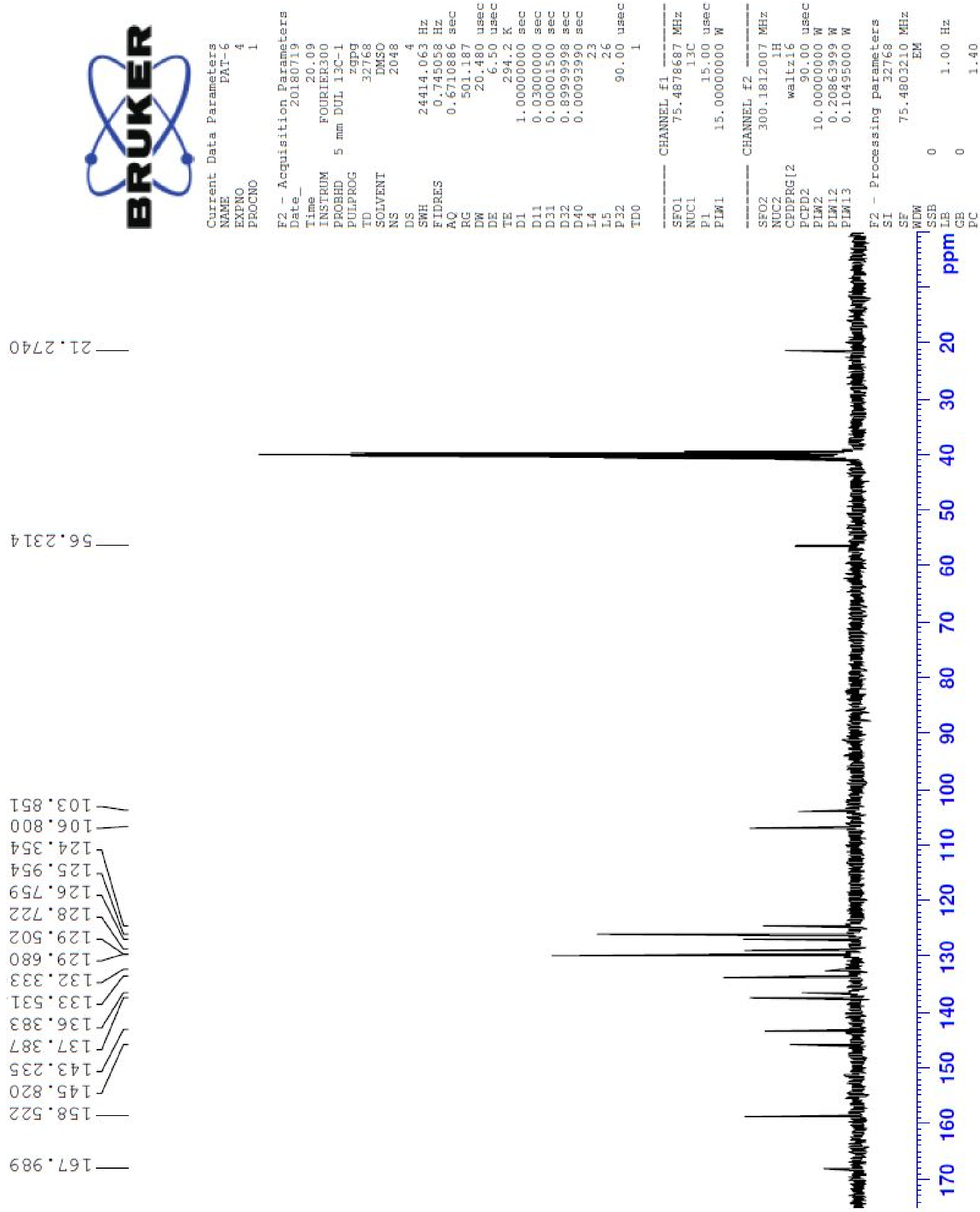

Figure S17. *Compound 4f* ^{13}*CNMR spectrum*

Data File: C:\LabSolutions\Data\Analiz\Lyuttas\PAT-7_20.lcd

Elmt	Val.	Min	Max	Elmt	Val.	Min	Max	Elmt	Val.	Min	Max	Elmt	Val.	Min	Max	Use Adduct
H	1	0	31	O	2	1	2	S	2	0	1	Ru	2	0	0	H
C	4	18	25	F	1	0	0	Cl	1	1	2	Pd	2	0	0	
N	3	4	5	P	3	0	0	Br	1	0	0	I	3	0	0	

Error Margin (ppm): 10
HC Ratio: unlimited
Max Isotopes: 3
MSn Iso RI (%): 10.00

DBE Range: 8.0 - 17.0
Apply N Rule: yes
Isotope RI (%): 1.00
MSn Logic Mode: AND

Electron Ions: both
Use MSn Info: yes
Isotope Res: 9000
Max Results: 500

Event#: 1 MS(E+) Ret. Time : 8.280 -> 8.680 Scan# : 1243 -> 1303

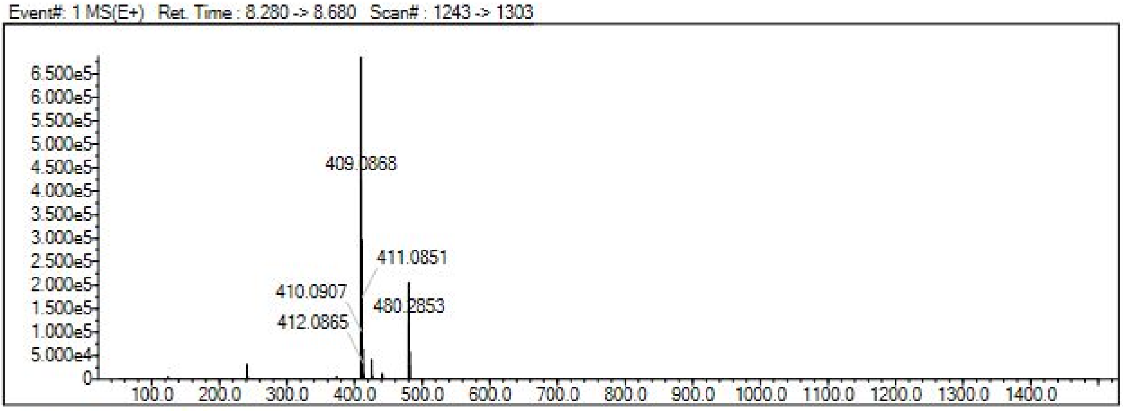

Measured region for 409.0868 m/z

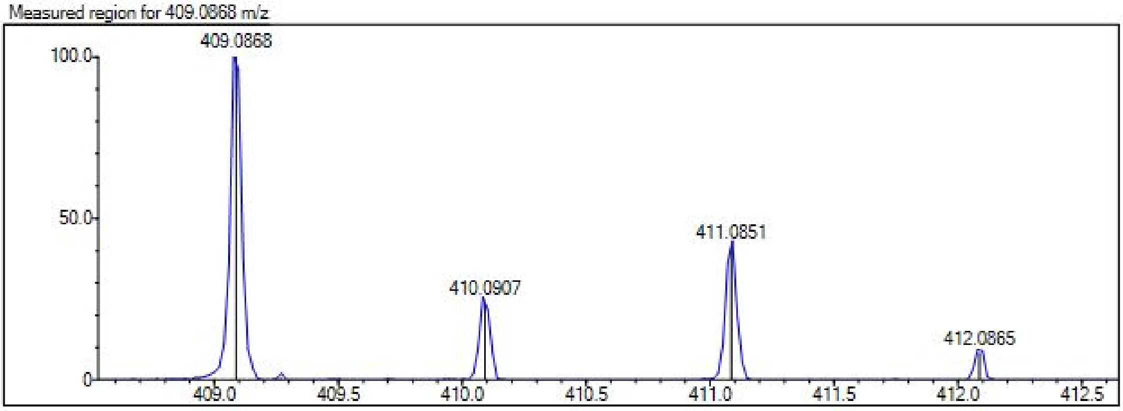

C21 H17 N4 O S Cl [M+H]+ : Predicted region for 409.0884 m/z

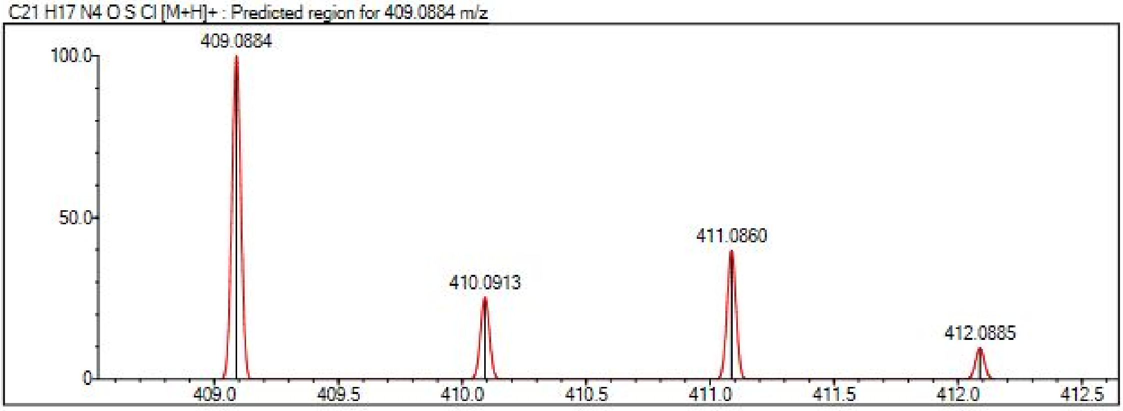

Rank	Score	Formula (M)	Ion	Meas. m/z	Pred. m/z	Df. (mDa)	Df. (ppm)	Iso	DBE
1	92.72	C21 H17 N4 O S Cl	[M+H]+	409.0868	409.0884	-1.6	-3.91	100.00	15.0

Figure S18. *Compound 4f HRMS spectrum*

1.7. 2-{2-[(2-Chloro-6-methoxyquinolin-3-yl)methylene]hydrazinyl}-4-(4-methoxyphenyl)thiazole (4g)

M.p: 176-180 °C. Appearance: Yellow powder. Yield: %73.

^{1}H-NMR (300 MHz, DMSO-*d6*): δ:3.78 (3H, s, phenyl O-CH_3), 3.91 (3H, s, O-CH_3), 6.97 (2H, d, *J*= 8.91 Hz, phenyl $H_{3,5}$), 7.24 (H, s, thiazole H_5), 7.43 (H, dd, J_1=2.79 Hz, J_2=9.19 Hz, quinoline H_7), 7.59 (H, d, *J*= 2.76 Hz, quinoline H_5), 7.79 (2H, d, *J*= 8.86 Hz, phenyl $H_{2,6}$), 7.82 (H, d, *J*=9.21 Hz, quinoline H_8), 8.42 (H, s, -N=C-H), 8.69 (H, s, quinoline H_4), 12.56 (H, brs, =N-N-H).

^{13}C-NMR (75 MHz, DMSO-*d6*): δ: 55.60 (C, s, phenyl O-CH_3), 56.22 (C, s, O-CH_3), 102.64 (C, s, thiazole C_5), 106.77 (C, s, quinoline C_5), 114.46 (2C, s, phenyl $C_{3,5}$), 124.34 (C, s, quinoline C_7), 126.72 (C, s, quinoline C_3), 127.37 (2C, s, phenyl $C_{2,6}$), 127. 67 (C, s, quinoline C_{4a}), 128.69 (C, s, quinoline C_8), 129.48 (C, s, phenyl C_1), 133.53 (C, s, quinoline C_4), 136.57 (C, s, thiazole C_4), 143.23 (C, s, N=C), 145.82 (C, s, quinoline C_{8a}), 150.65 (C, s, quinoline C_2), 158.51 (C, s, quinoline C_6), 159.34 (C, s, phenyl C_4), 168.02 (C, s, thiazole C_2).

HRMS (-m/z): $[M+H]^+$: For $C_{21}H_{17}N_4O_2SCl$ calculated: 425.0834, found: 425.0843.

InChI=1S/C21H17ClN4O2S/c1-27-16-5-3-13(4-6-16)19-12-29-21(25-19)26-23-11-15-9-14-10-17(28-2)7-8-18(14)24-20(15)22/h3-12H,1-2H3,(H,25,26)/b23-11+

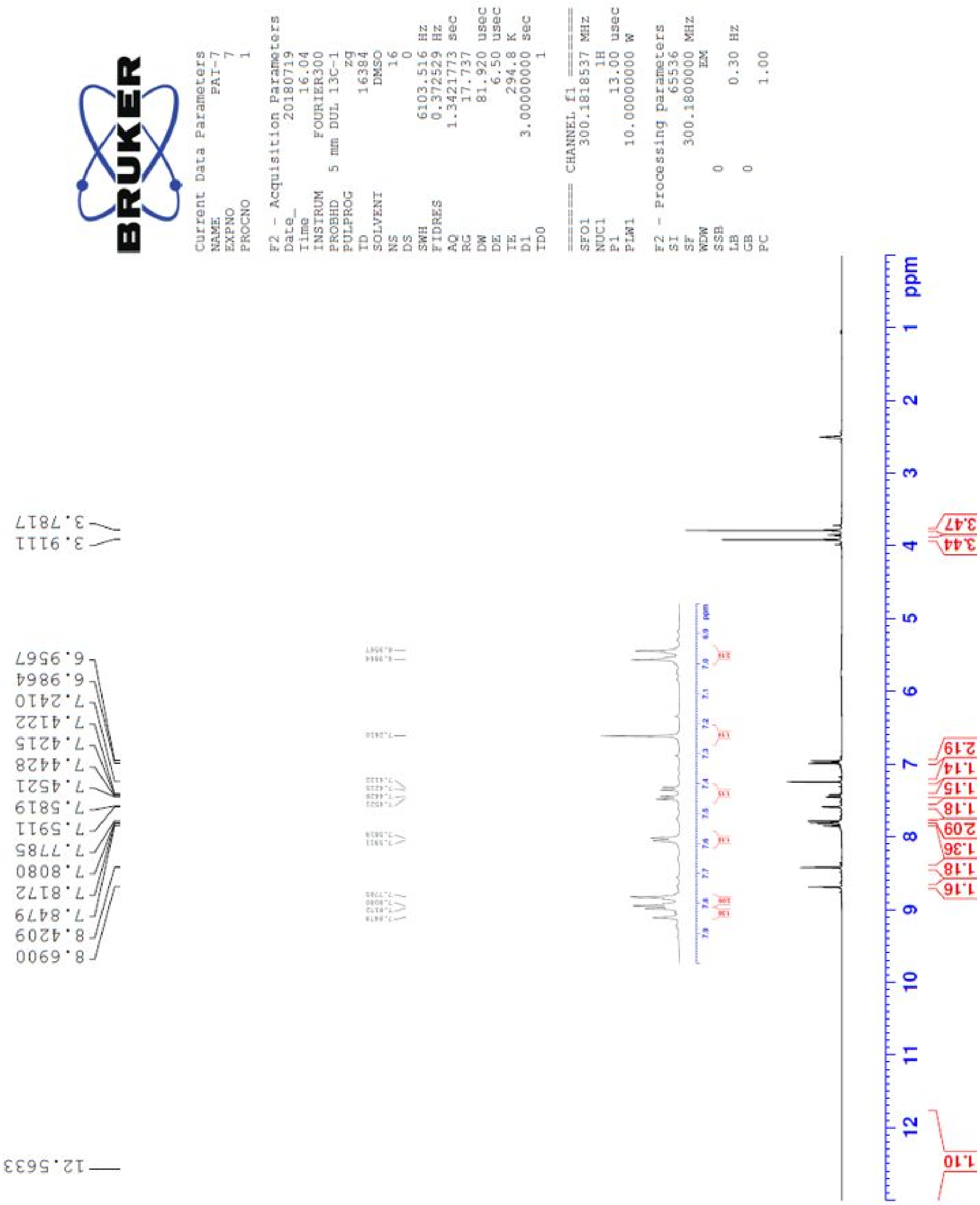

Figure S19. *Compound 4g ¹HNMR spectrum*

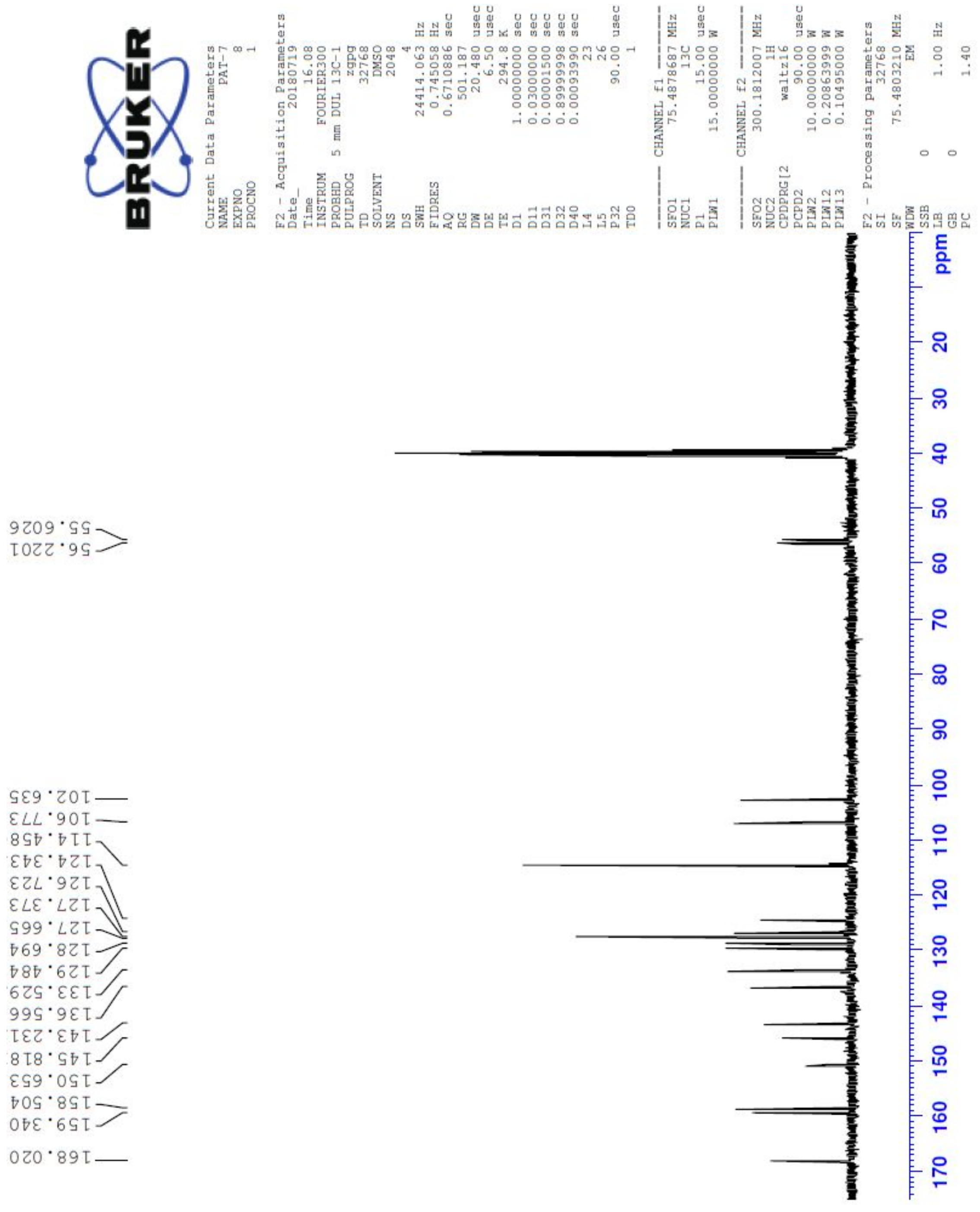

Figure S20. *Compound 4g* ^{13}*CNMR spectrum*

Data File: C:\LabSolutions\Data\Analiz\Lyuttas\PAT-7_26.lcd

Elmt	Val.	Min	Max	Elmt	Val.	Min	Max	Elmt	Val.	Min	Max	Elmt	Val.	Min	Max	Use Adduct
H	1	0	31	O	2	1	2	S	2	0	1	Ru	2	0	0	H
C	4	0	23	F	1	0	0	Cl	1	1	2	Pd	2	0	0	
N	3	4	5	P	3	0	0	Br	1	0	0	I	3	0	0	

Error Margin (ppm): 10
HC Ratio: unlimited
Max Isotopes: 3
MSn Iso RI (%): 10.00

DBE Range: 8.0 - 15.0
Apply N Rule: yes
Isotope RI (%): 1.00
MSn Logic Mode: AND

Electron Ions: both
Use MSn Info: yes
Isotope Res: 9000
Max Results: 500

Event#: 1 MS(E+) Ret. Time : 9.933 -> 10.107 Scan# : 1491 -> 1517

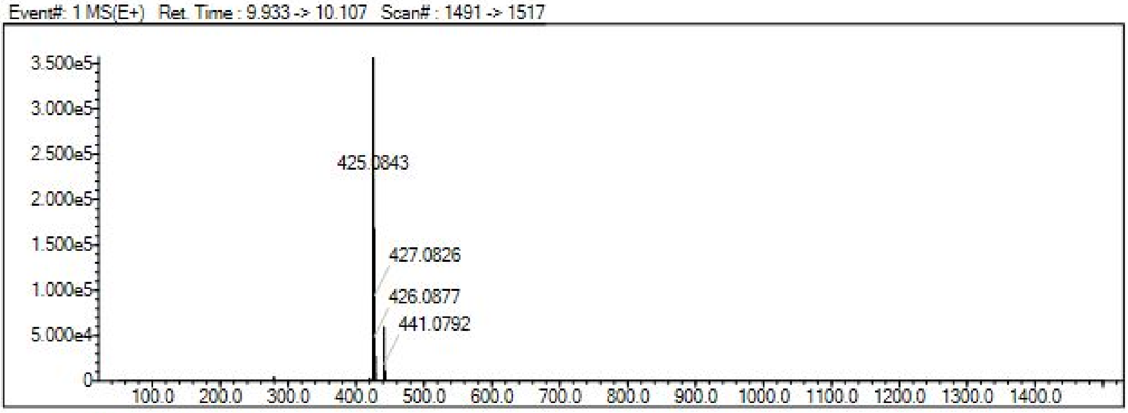

Measured region for 425.0843 m/z

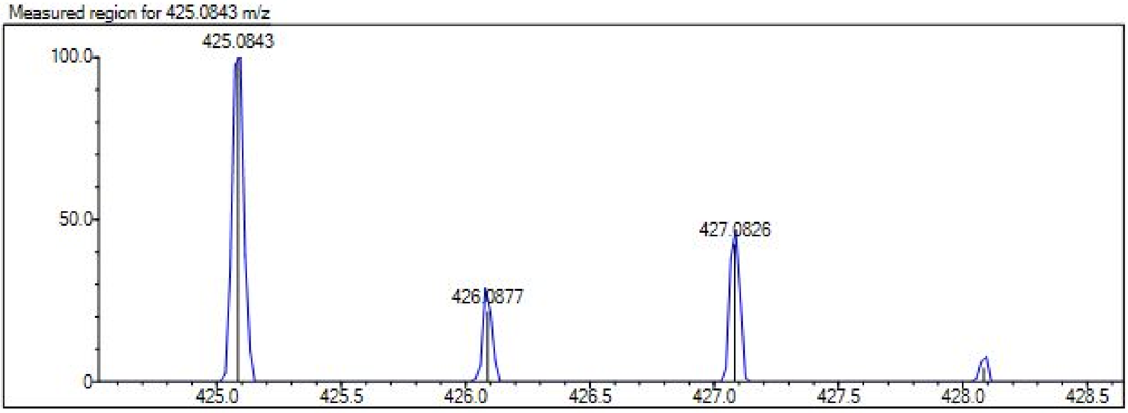

C21 H17 N4 O2 S Cl [M+H]+ : Predicted region for 425.0834 m/z

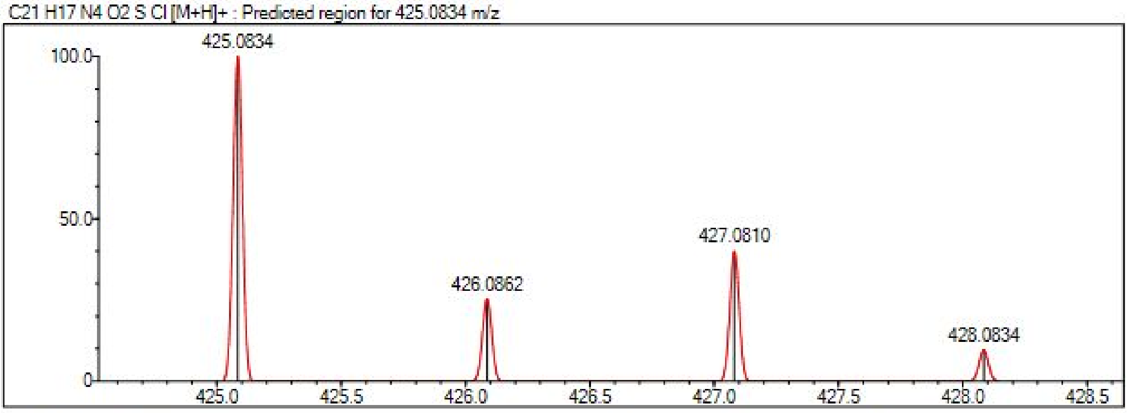

Rank	Score	Formula (M)	Ion	Meas. m/z	Pred. m/z	Df. (mDa)	Df. (ppm)	Iso	DBE
1	81.42	C21 H17 N4 O2 S Cl	[M+H]+	425.0843	425.0834	0.9	2.12	83.77	15.0

Figure S21. *Compound 4g HRMS spectrum*

1.8. 2-{2-[(2-Chloro-6-methoxyquinolin-3-yl)methylene]hydrazinyl}-4-(3-chlorophenyl)thiazole (4h)

M.p: 200-202 °C. Appearance: Yellow powder. Yield: %75.

^{1}H-NMR (300 MHz, DMSO-*d6*): δ: 3.92 (3H, s, O-CH_3), 7.35-7.38 (H, m, phenyl H_5), 7.42-7.48 (2H, m, quinoline H_7, phenyl H_6), 7.59 (H, s, thiazole H_5), 7.60 (H, d, *J*= 2.79 Hz, quinoline H_5), 7.82-7.86 (2H, m, quinoline H_8, phenyl H_4), 7.91 (H, s, phenyl H_2), 8.43 (H, s, -N=C-H), 8.71 (H, s, quinoline H_4), 12.63 (H, brs, =N-N-H).

^{13}C-NMR (75 MHz, DMSO-*d6*): δ: 56.23 (C, s, O-CH_3), 106.40 (C, s, thiazole C_5), 106.80 (C, s, quinoline C_5), 124.40 (C, s, quinoline C_7), 124.53 (C, s, phenyl C_6), 125.68 (C, s, phenyl C_4),126.67 (C, s, quinoline C_3), 127.81 (C, s, quinoline C_{4a}),128.70 (C, s, quinoline C_8), 129.50 (C, s, phenyl C_5), 131.04 (C, s, phenyl C_2), 133.63 (C, s, phenyl C_1), 133.96 (C, s, phenyl C_3), 136.76 (C, s, quinoline C_4), 136.97 (C, s, thiazole C_4), 143.27 (C, s, N=C), 145.82 (C, s, quinoline C_{8a}), 149.49 (C, s, quinoline C_2), 158.52 (C, s, quinoline C_6), 168.24 (C, s, thiazole C_2).

HRMS (-m/z): [M+H]$^+$: For $C_{20}H_{14}N_4OSCl_2$ calculated: 429.0338, found: 429.0344

InChI=1S/C20H14Cl2N4OS/c1-27-16-5-6-17-13(9-16)7-14(19(22)24-17)10-23-26-20-25-18(11-28-20)12-3-2-4-15(21)8-12/h2-11H,1H3,(H,25,26)/b23-10+

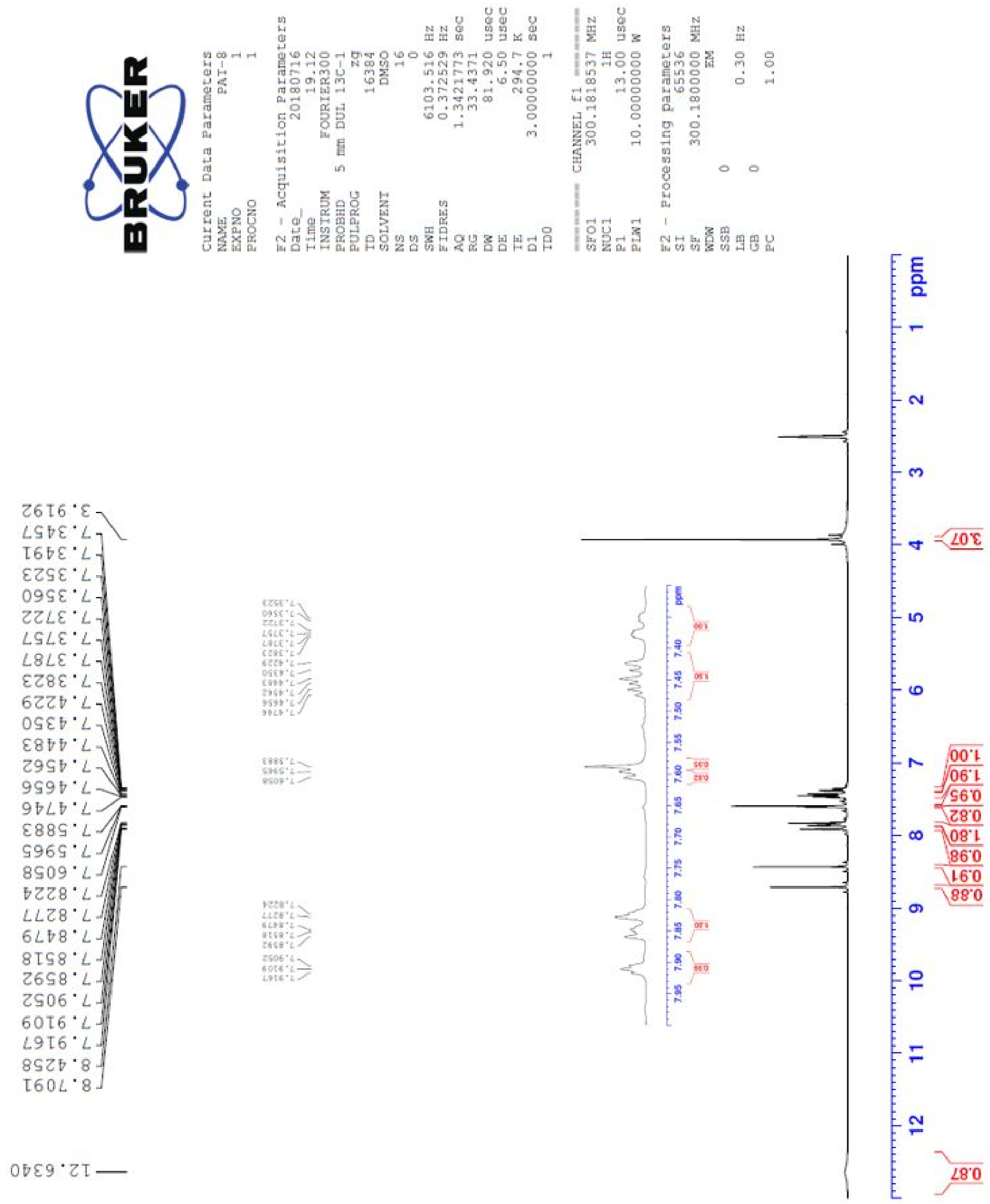

Figure S22. *Compound 4h* [1]*HNMR spectrum*

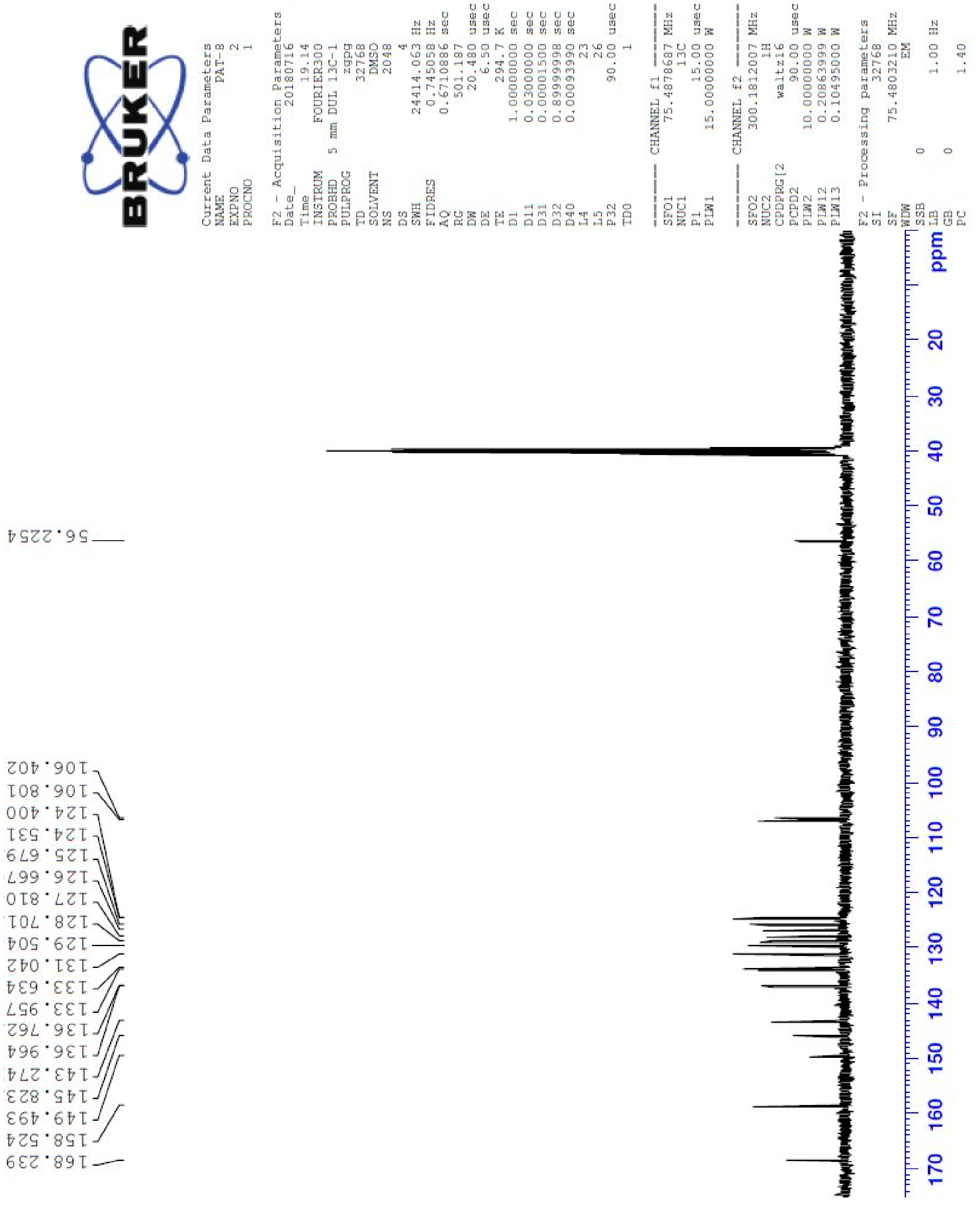

Figure S23. *Compound 4h ^{13}CNMR spectrum*

Formula Predictor Report - PAT-8'_23.lcd Page 1 of 1

Data File: C:\LabSolutions\Data\Analiz\Lyuttas\PAT-8'_23.lcd

Elmt	Val.	Min	Max	Elmt	Val.	Min	Max	Elmt	Val.	Min	Max	Elmt	Val.	Min	Max	Use Adduct
H	1	0	31	O	2	1	3	S	2	0	1	Ru	2	0	0	H
C	4	18	25	F	1	0	0	Cl	1	1	2	Pd	2	0	0	
N	3	4	5	P	3	0	0	Br	1	0	0	I	3	0	0	

Error Margin (ppm):	10	DBE Range:	8.0 - 17.0	Electron Ions:	both
HC Ratio:	unlimited	Apply N Rule:	yes	Use MSn Info:	yes
Max Isotopes:	3	Isotope RI (%):	1.00	Isotope Res:	9000
MSn Iso RI (%):	10.00	MSn Logic Mode:	AND	Max Results:	500

Event#: 1 MS(E+) Ret. Time : 8.773 -> 8.960 Scan# : 1317 -> 1345

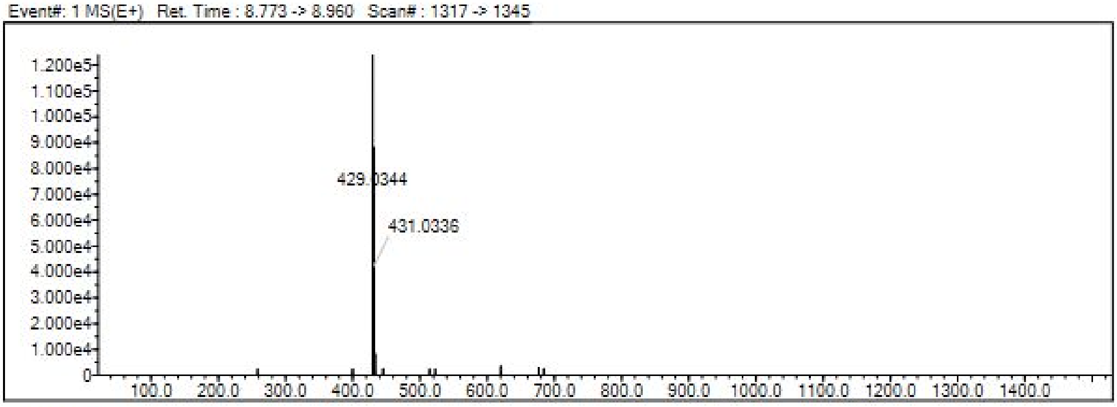

Measured region for 429.0344 m/z

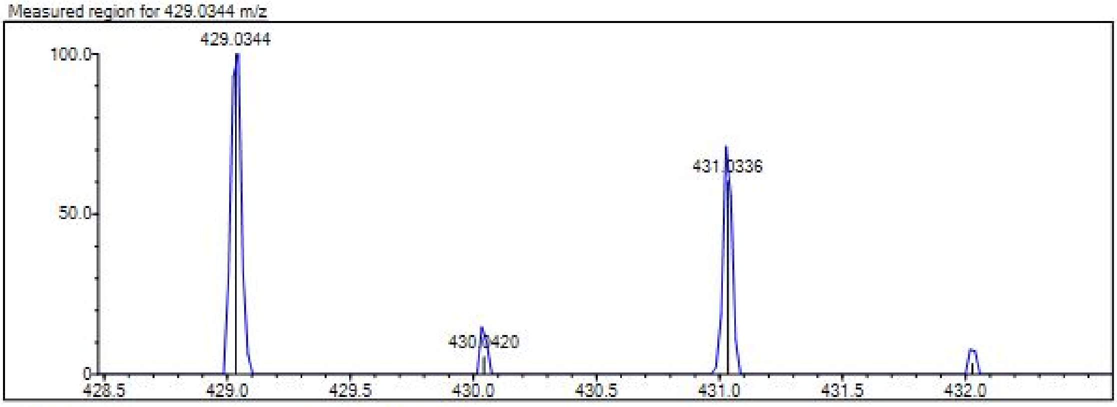

C20 H14 N4 O S Cl2 [M+H]+ : Predicted region for 429.0338 m/z

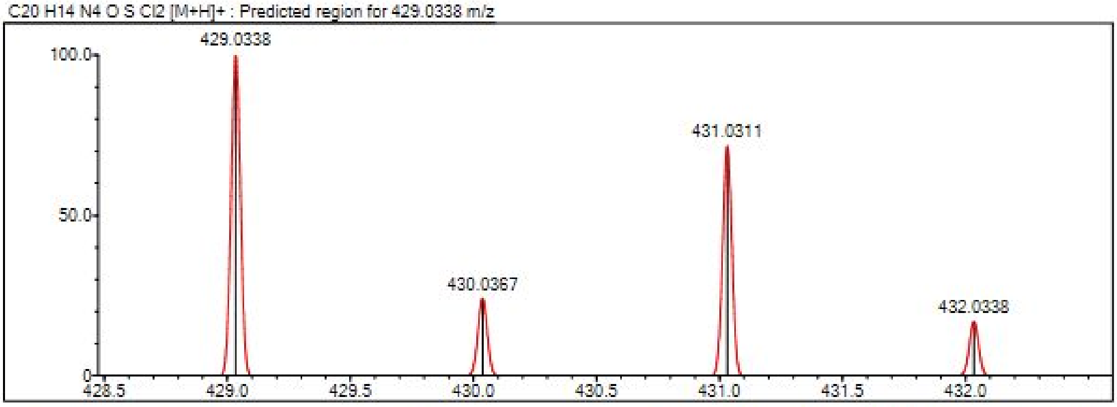

Rank	Score	Formula (M)	Ion	Meas. m/z	Pred. m/z	Df. (mDa)	Df. (ppm)	Iso	DBE
1	51.31	C20 H14 N4 O S Cl2	[M+H]+	429.0344	429.0338	0.6	1.40	51.82	15.0

Figure S24. *Compound 4h HRMS spectrum*

1.9. 2-{2-[(2-Chloro-6-methoxyquinolin-3-yl)methylene]hydrazinyl}-4-(3-nitrophenyl)thiazole (4i)

M.p: 212-214 °C. Appearance: Dark yellow powder. Yield: %79.

^{1}H-NMR (300 MHz, DMSO-*d6*): δ: 3.92 (3H, s, O-CH_3), 7.43 (H, dd, J_1=2.79 Hz, J_2=9.17 Hz, quinoline H_7), 7.59 (H, d, *J*= 2.76 Hz, quinoline H_5), 7.70 (H, t, *J*=8.02 Hz, phenyl H_5), 7.75 (H, s, thiazole H_5), 7.83 (H, d, *J*=9.19 Hz, quinoline H_8), 8.12-8.16 (H, m, phenyl H_4), 8.29 (H, brd, *J*= 8.17 Hz, phenyl H_6), 8.41 (H, s, -N=C-H), 8.65 (H, s, phenyl H_2), 8.70 (H, s, quinoline H_4), 12.72 (H, brs, =N-N-H).

^{13}C-NMR (75 MHz, DMSO-*d6*): δ: 56.22 (C, s, O-CH_3), 106.79 (C, s, quinoline C_5), 107.49 (C, s, thiazole C_5), 120.39 (C, s, phenyl C_5), 122.57 (C, s, phenyl C_4), 124.40 (C, s, quinoline C_7), 126.60 (C, s, quinoline C_3), 128.67 (C, s, quinoline C_{4a}), 129.50 (2C, s, quinoline C8, phenyl C_2), 130.71 (C, s, phenyl C_1), 132.02 (C, s, phenyl C_6), 133.65 (C, s, phenyl C_3), 136.42 (C, s, quinoline C_4), 136.89 (C, s, thiazole C_4), 143.28 (C, s, N=C), 145.81 (C, s, quinoline C_{8a}), 148.70 (C, s, quinoline C_2), 158.51 (C, s, quinoline C_6), 168.47 (C, s, thiazole C_2).

HRMS (-m/z): [M+H]$^+$: For $C_{20}H_{14}N_5O_3SCl$ calculated: 440.0579, found: 440.0599

InChI=1S/C20H14ClN5O3S/c1-29-16-5-6-17-13(9-16)7-14(19(21)23-17)10-22-25-20-24-18(11-30-20)12-3-2-4-15(8-12)26(27)28/h2-11H,1H3,(H,24,25)/b22-10+

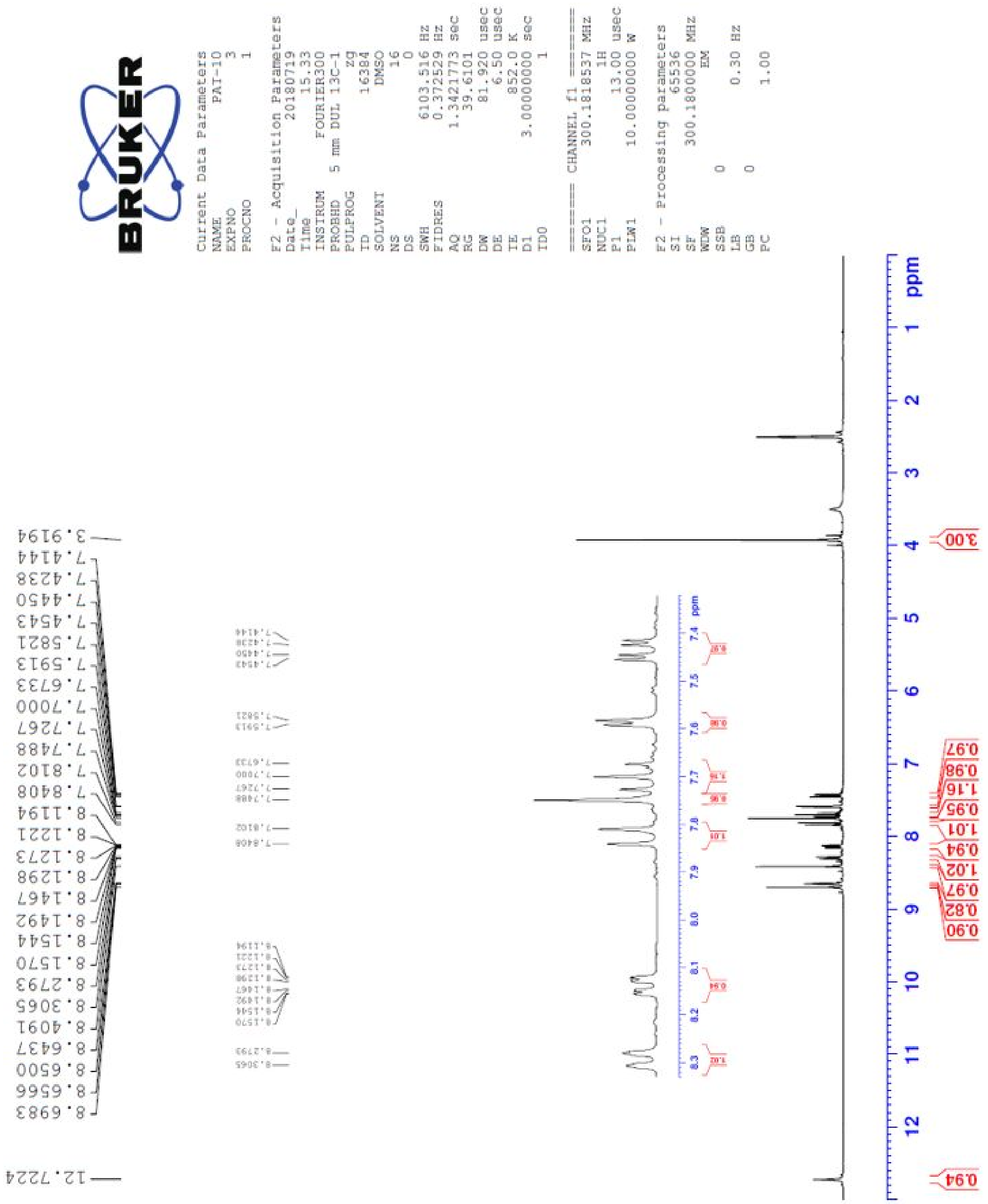

Figure S25. *Compound 4i ^{1}HNMR spectrum*

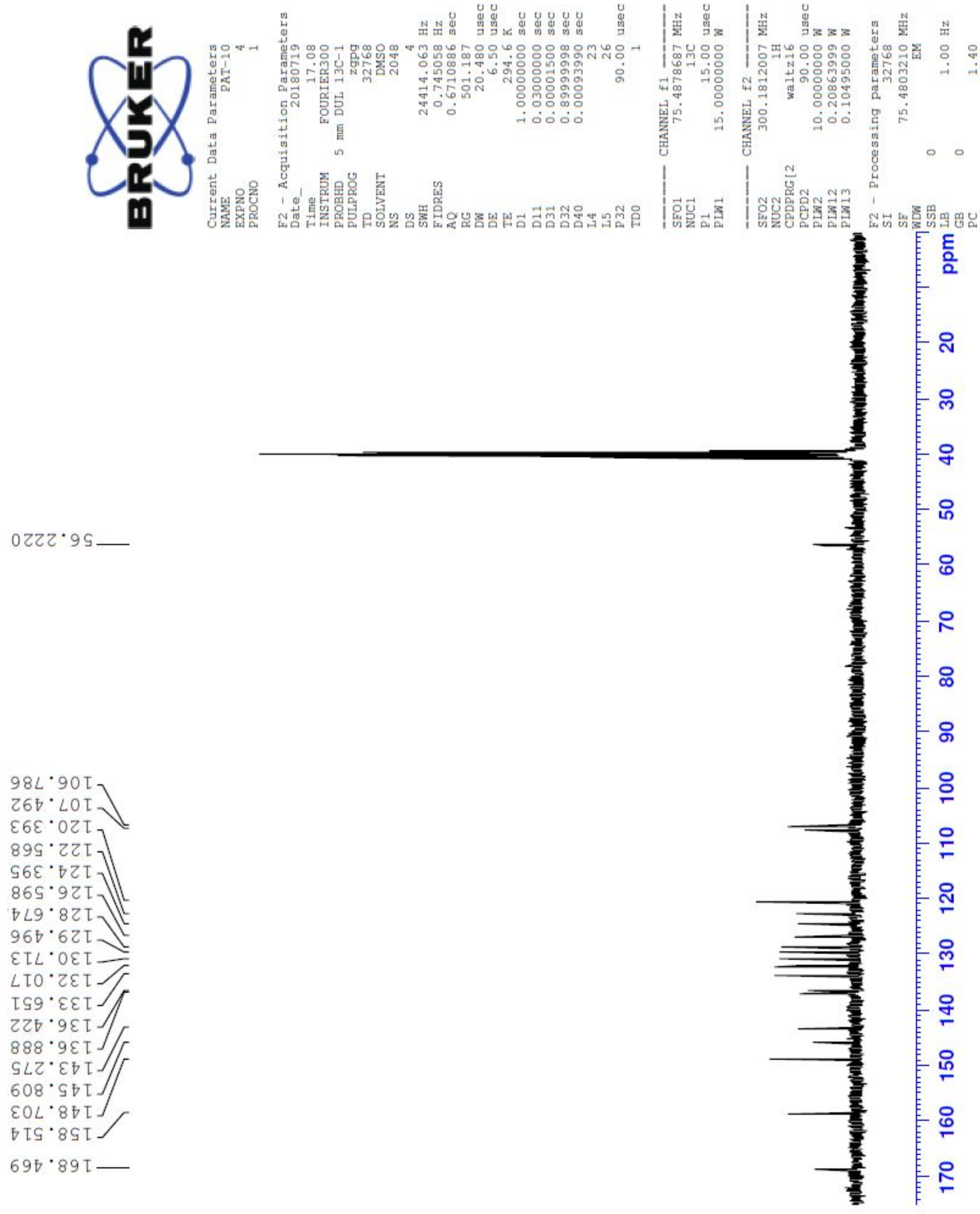

Figure S26. *Compound 4i ^{13}CNMR spectrum*

Data File: C:\LabSolutions\Data\Analiz\Lyuttas\PAT-10_24.lcd

Elmt	Val.	Min	Max	Elmt	Val.	Min	Max	Elmt	Val.	Min	Max	Elmt	Val.	Min	Max	Use Adduct
H	1	0	31	O	2	1	3	S	2	0	1	Ru	2	0	0	H
C	4	0	25	F	1	0	1	Cl	1	1	2	Pd	2	0	0	
N	3	4	5	P	3	0	0	Br	1	0	0	I	3	0	0	

Error Margin (ppm):	10	DBE Range:	8.0 - 21.0	Electron Ions:	both
HC Ratio:	unlimited	Apply N Rule:	yes	Use MSn Info:	yes
Max Isotopes:	3	Isotope RI (%):	1.00	Isotope Res:	9000
MSn Iso RI (%):	10.00	MSn Logic Mode:	AND	Max Results:	500

Event#: 1 MS(E+) Ret. Time : 10.120 -> 10.267 Scan# : 1519 -> 1541

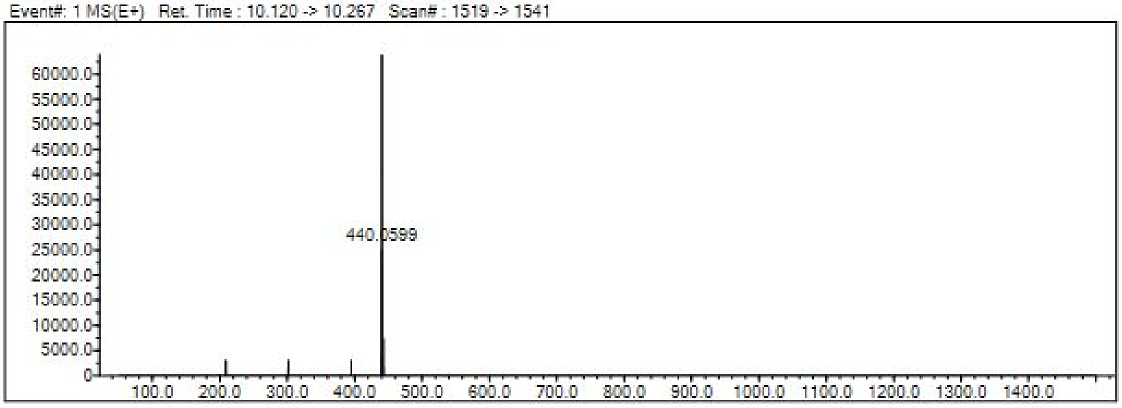

Measured region for 440.0599 m/z

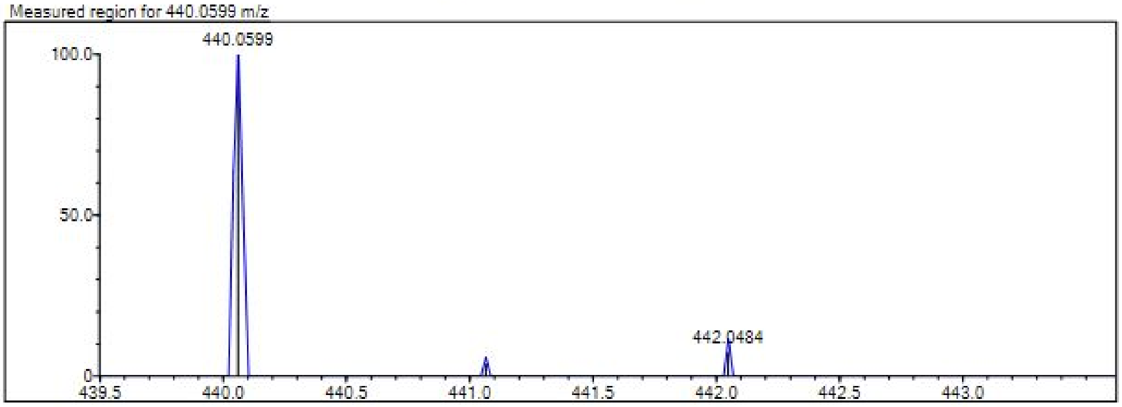

C20 H14 N5 O3 S Cl [M+H]+ : Predicted region for 440.0579 m/z

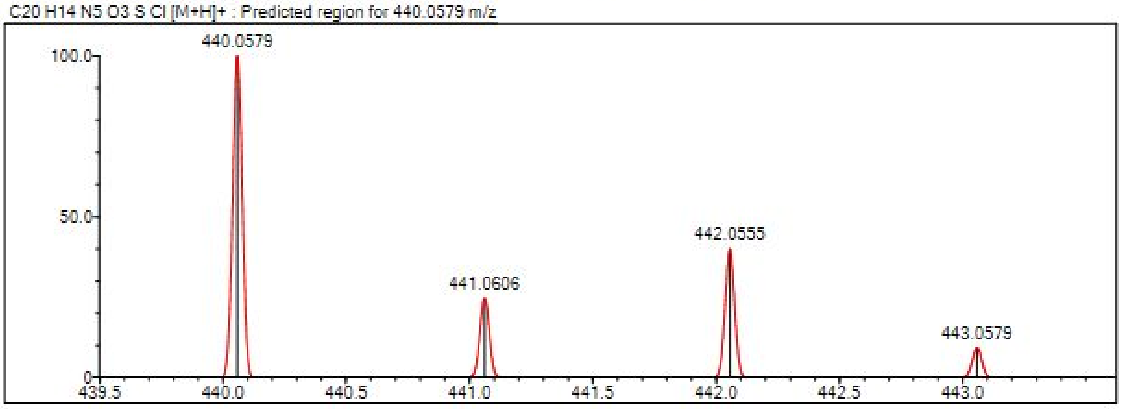

Rank	Score	Formula (M)	Ion	Meas. m/z	Pred. m/z	Df. (mDa)	Df. (ppm)	Iso	DBE
1	30.77	C20 H14 N5 O3 S Cl	[M+H]+	440.0599	440.0579	2.0	4.54	33.76	16.0

Figure S27. *Compound 4i HRMS spectrum*

1.10. 2-{2-[(2-Chloro-6-methoxyquinolin-3-yl)methylene]hydrazinyl}-4-(3-florophenyl)thiazole (4j)

M.p: 221-223 °C. Appearance: Yellow powder. Yield: %76.

^{1}H-NMR (300 MHz, DMSO-*d6*): δ: 3.92 (3H, s, O-CH_3), 7.14 (H, td, J_1= 2.46 Hz, J_2= 8.54 Hz, phenyl H_5), 7.43-7.50 (2H, m, quinoline H_7, phenyl H_2), 7.56 (H, s, thiazole H_5), 7.60 (H, d, *J*= 2.73 Hz, quinoline H_5), 7.63-7.68 (H, m, phenyl H_4), 7.72 (H, d, *J*= 7.86 Hz, phenyl H_6), 7.85 (H, d, *J*= 9.18 Hz, quinoline H_8), 8.43 (H, s, -N=C-H), 8.71 (H, s, quinoline H_4), 12.65 (H, brs, =N-N-H).

^{13}C-NMR (75 MHz, DMSO-*d6*): δ: 56.23 (C, s, O-CH_3), 106.29 (C, s, thiazole C_5), 106.80 (C, s, quinoline C_5), 112.37 and 112.67 (C, s, phenyl C_4), 114.62 and 114.90 (C, s, phenyl C_2), 122.05 (C, s, phenyl C_6), 124.39 (C, s, quinoline C_7), 126.67 (C, s, quinoline C_3), 128.70 (C, s, quinoline C_{4a}), 129.50 (C, s, phenyl C_5), 131.19 (2C, s, quinoline C_8, thiazole C_4), 133.62 (C, s, phenyl C_1), 136.72 (C, s, quinoline C_4), 143.27 (C, s, N=C), 145.82 (C, s, quinoline C_{8a}), 158.52 (C, s, quinoline C_6), 161.39 (C, s, phenyl C_3), 164.60 (C, s, quinoline C_2), 168.19 (C, s, thiazole C_2).

HRMS (-m/z): $[M+H]^+$: For $C_{20}H_{14}N_4OSFCl$ calculated: 413.0634, found: 413.0651

InChI=1S/C20H14ClFN4OS/c1-27-16-5-6-17-13(9-16)7-14(19(21)24-17)10-23-26-20-25-18(11-28-20)12-3-2-4-15(22)8-12/h2-11H,1H3,(H,25,26)/b23-10+

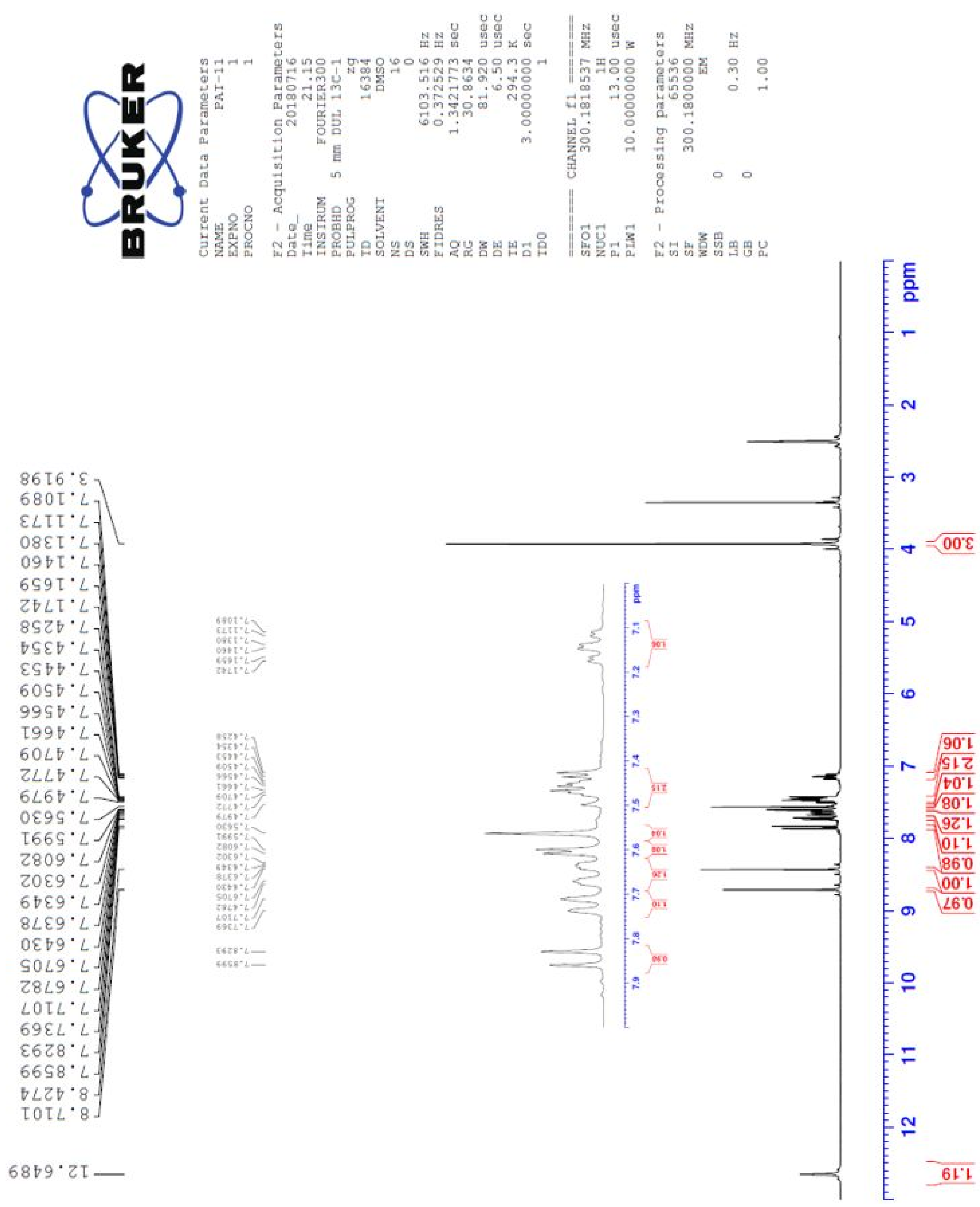

Figure S28. *Compound 4j ¹HNMR spectrum*

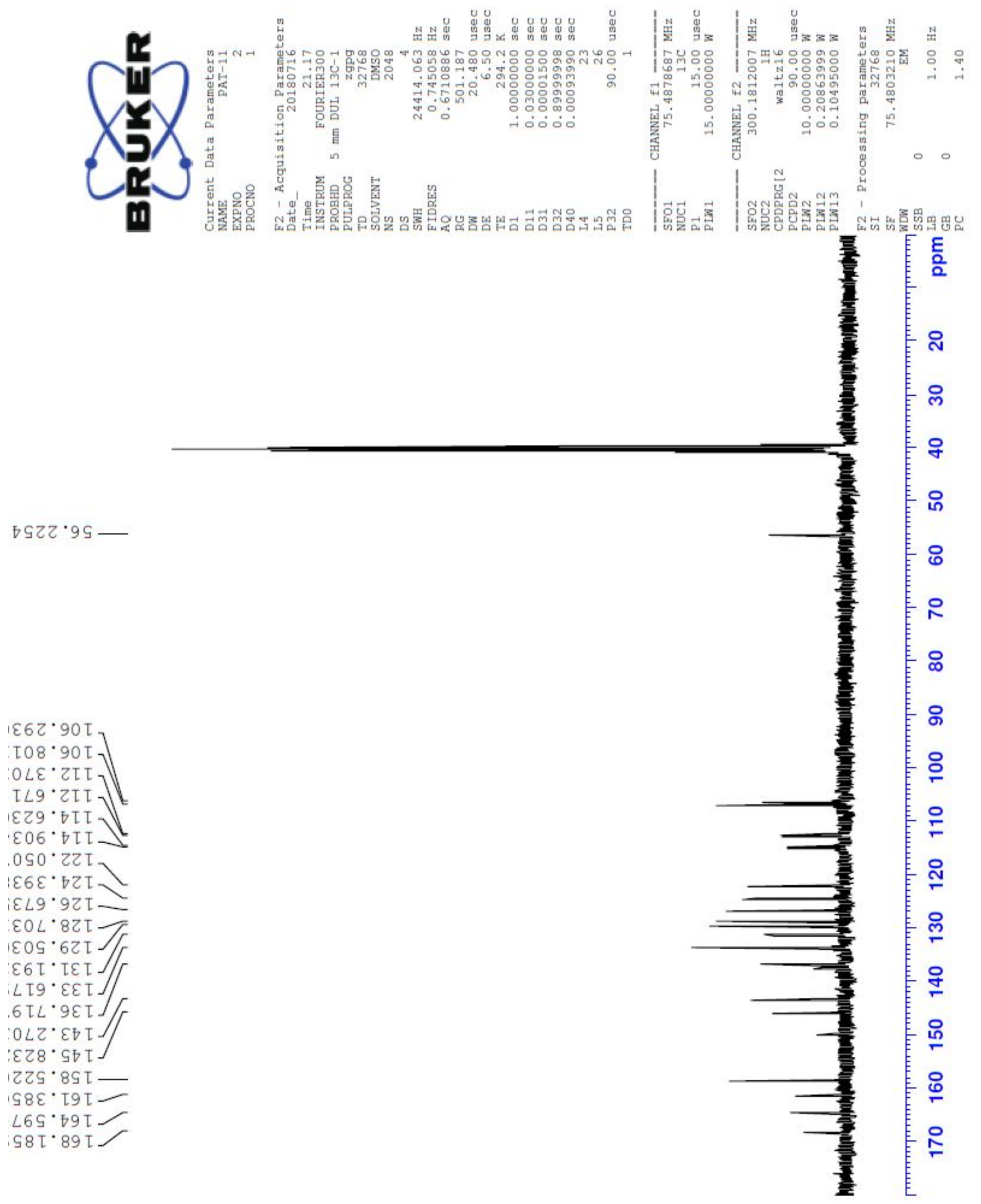

Figure S29. *Compound 4j* ^{13}*CNMR spectrum*

Data File: C:\LabSolutions\Data\Analiz\Lyuttas\PAT-12_26.lcd

Elmt	Val.	Min	Max	Elmt	Val.	Min	Max	Elmt	Val.	Min	Max	Elmt	Val.	Min	Max	Use Adduct
H	1	0	31	O	2	1	2	S	2	0	1	Ru	2	0	0	H
C	4	0	25	F	1	0	1	Cl	1	1	2	Pd	2	0	0	
N	3	4	5	P	3	0	0	Br	1	0	0	I	3	0	0	

Error Margin (ppm): 10
HC Ratio: unlimited
Max Isotopes: 3
MSn Iso RI (%): 10.00

DBE Range: 8.0 - 20.0
Apply N Rule: yes
Isotope RI (%): 1.00
MSn Logic Mode: AND

Electron Ions: both
Use MSn Info: yes
Isotope Res: 9000
Max Results: 500

Event#: 1 MS(E+) Ret. Time : 6.773 -> 7.027 Scan# : 1017 -> 1055

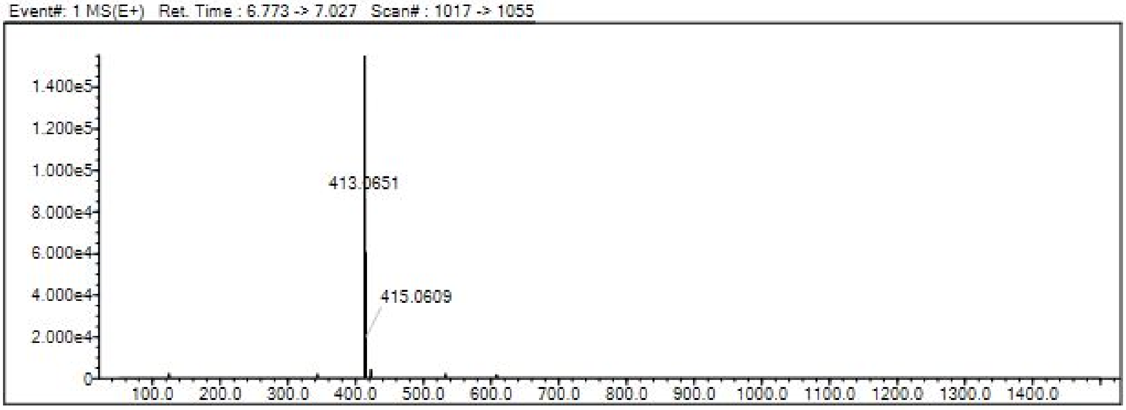

Measured region for 413.0651 m/z

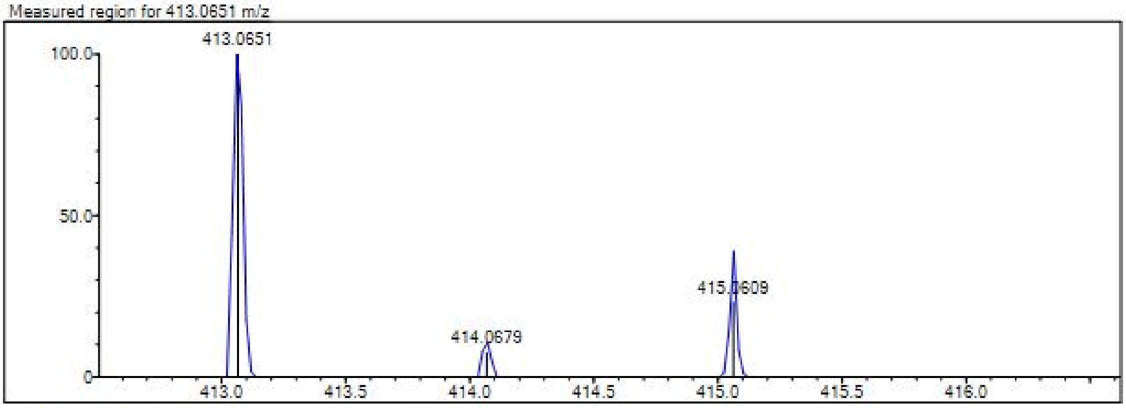

C20 H14 N4 O F S Cl [M+H]+ : Predicted region for 413.0634 m/z

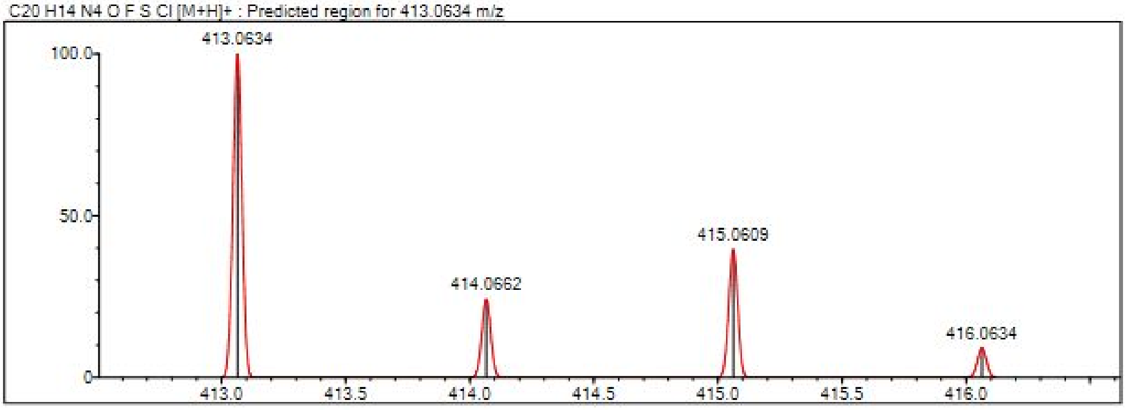

Rank	Score	Formula (M)	Ion	Meas. m/z	Pred. m/z	Df. (mDa)	Df. (ppm)	Iso	DBE
1	58.47	C20 H14 N4 O F S Cl	[M+H]+	413.0651	413.0634	1.7	4.12	63.41	15.0

Figure S30. *Compound 4j HRMS spectrum*

1.11. 2-{2-[(2-Chloro-6-methoxyquinolin-3-yl)methylene]hydrazinyl}-4-(3-methoxyphenyl)thiazole (4k)

M.p: 217-220 °C. Appearance: Dark yellow powder. Yield: %70.

^{1}H-NMR (300 MHz, DMSO-*d6*): δ: 3.80 (3H, s, phenyl O-CH_3), 3.92 (3H, s, O-CH_3), 6.87-6.90 (H, m, phenyl H_4), 7.33 (H, t, *J*=7.91 Hz, phenyl H_5), 7.43-7.46 (3H, m, quinoline H_7, phenyl H_2, H_6), 7.47 (H, s, thiazole H_5), 7.61 (H, d, *J*=2.77 Hz, quinoline H_5), 7.85 (H, d, *J*=9.18 Hz, quinoline H_8), 8.43 (H, s, -N=C-H), 8.72 (H, s, quinoline H_4), 12.66 (H, brs, =N-N-H).

^{13}C-NMR (75 MHz, DMSO-*d6*): δ: 55. 55 (C, s, phenyl O-CH_3), 56.24 (C, s, O-CH_3), 105.19 (C, s, thiazole C_5), 106.79 (C, s, quinoline C_5), 111. 22 (C, s, phenyl C_2), 113.96 (C, s, phenyl C_4), 118.40 (C, s, phenyl C_6), 124.40 (C, s, quinoline C_7), 126.72 (C, s, quinoline C_3), 128.72 (C, s, quinoline C_{4a}), 129.50 (C, s, quinoline C_8), 130.19 (C, s, phenyl C_5), 133.59 (C, s, phenyl C_1), 136.26 (C, s, thiazole C_4), 136.60 (C, s, quinoline C_4), 143.82 (C, s, N=C), 145.82 (C, s, quinoline C_{8a}), 150.79 (C, s, quinoline C_2), 158.52 (C, s, quinoline C_6), 159.98 (C, s, phenyl C_3), 168.01 (C, s, thiazole C_2).

HRMS (-m/z): $[M+H]^+$: For $C_{21}H_{17}N_4O_2SCl$ calculated: 425.0834, found: 425.0855

InChI=1S/C21H17ClN4O2S/c1-27-16-5-3-4-13(9-16)19-12-29-21(25-19)26-23-11-15-8-14-10-17(28-2)6-7-18(14)24-20(15)22/h3-12H,1-2H3,(H,25,26)/b23-11+

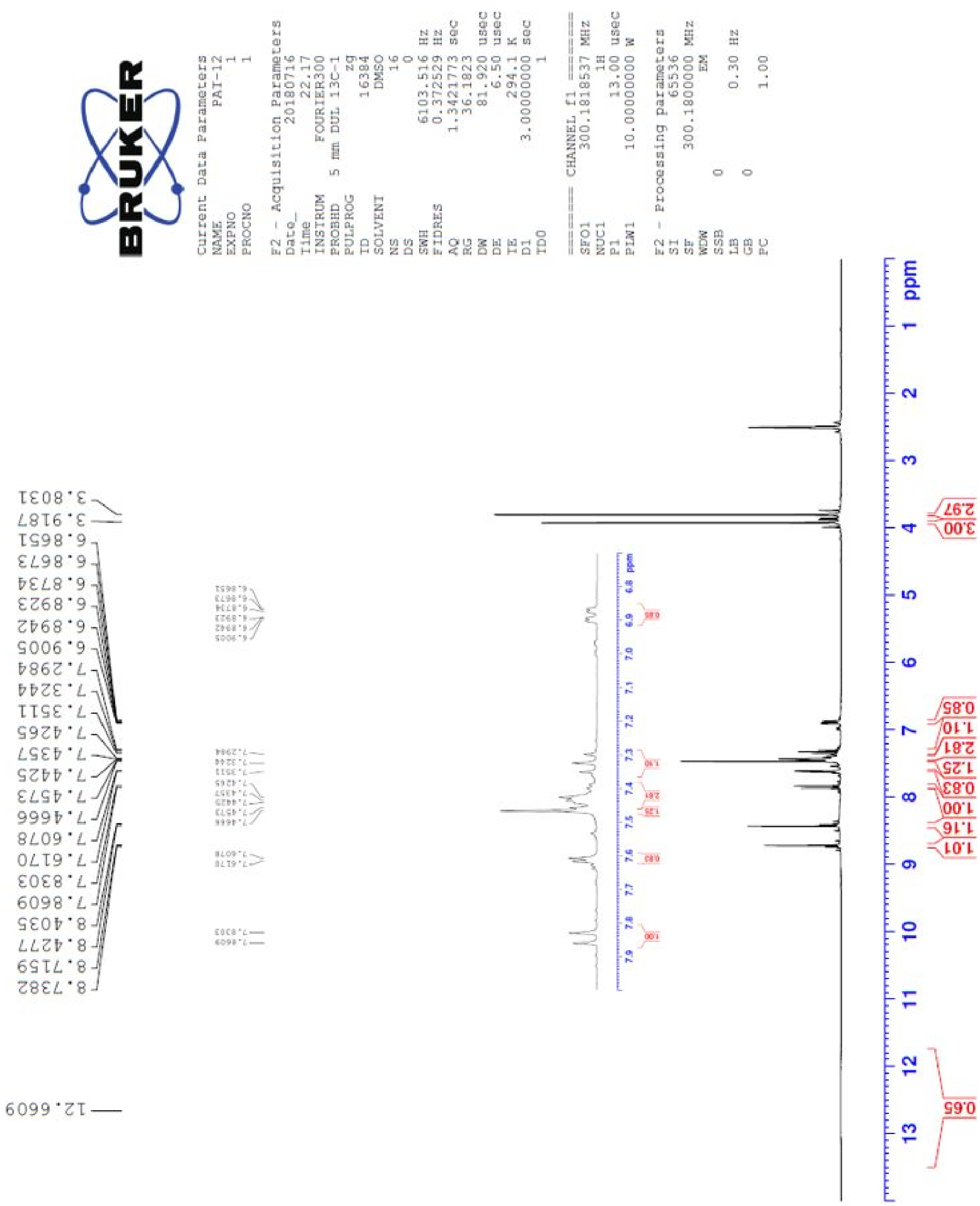

Figure S31. *Compound 4k ^{1}HNMR spectrum*

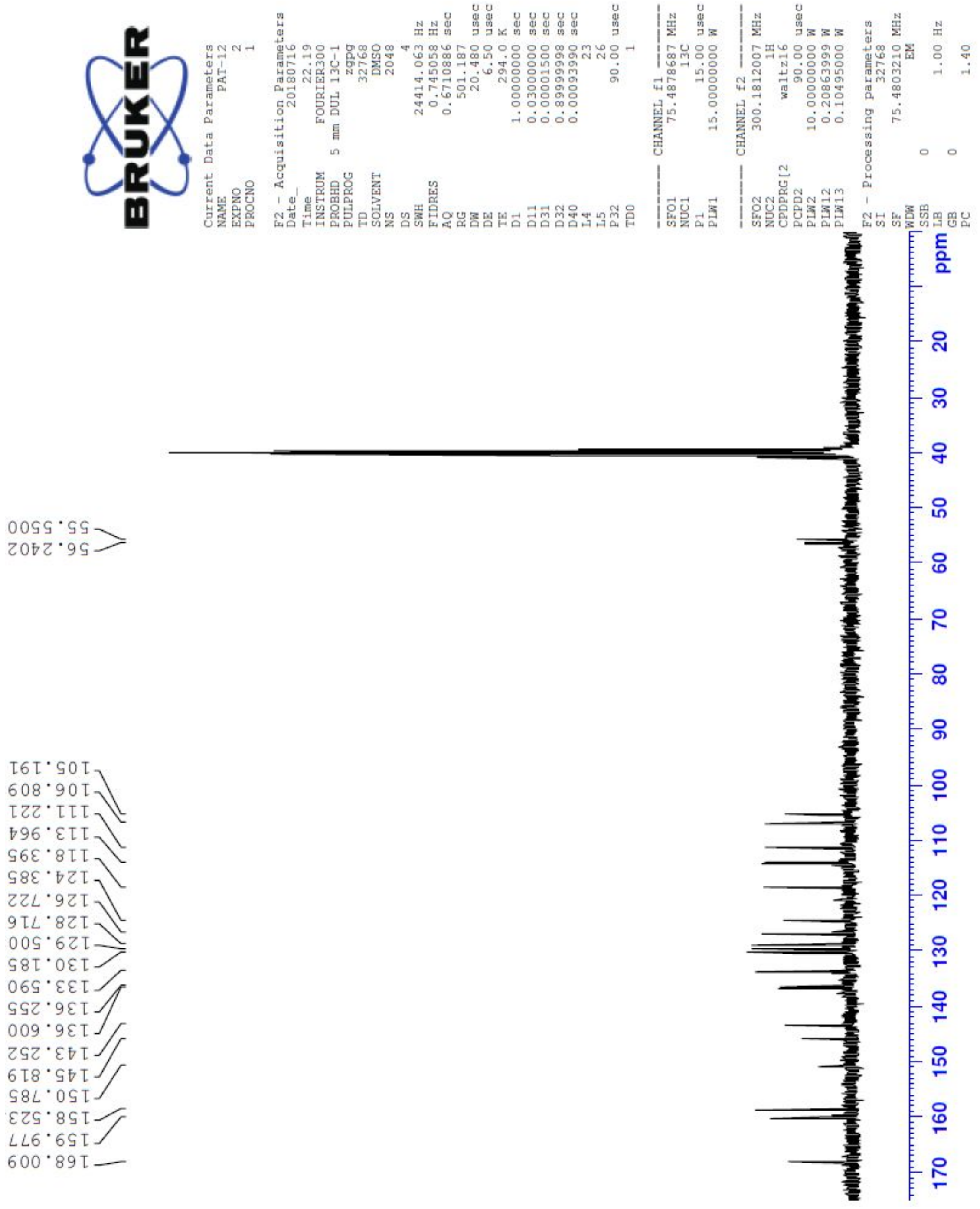

Figure S32. *Compound 4k* ^{13}*CNMR spectrum*

Data File: C:\LabSolutions\Data\Analiz\Lyuttas\PAT-12_26.lcd

Elmt	Val.	Min	Max	Elmt	Val.	Min	Max	Elmt	Val.	Min	Max	Elmt	Val.	Min	Max	Use Adduct
H	1	0	31	O	2	1	2	S	2	0	1	Ru	2	0	0	H
C	4	0	25	F	1	0	0	Cl	1	1	2	Pd	2	0	0	
N	3	4	5	P	3	0	0	Br	1	0	0	I	3	0	0	

Error Margin (ppm):	10	DBE Range:	8.0 - 20.0	Electron Ions:	both
HC Ratio:	unlimited	Apply N Rule:	yes	Use MSn Info:	yes
Max Isotopes:	3	Isotope RI (%):	1.00	Isotope Res:	9000
MSn Iso RI (%):	10.00	MSn Logic Mode:	AND	Max Results:	500

Event#: 1 MS(E+) Ret. Time : 9.933 -> 10.173 Scan# : 1491 -> 1527

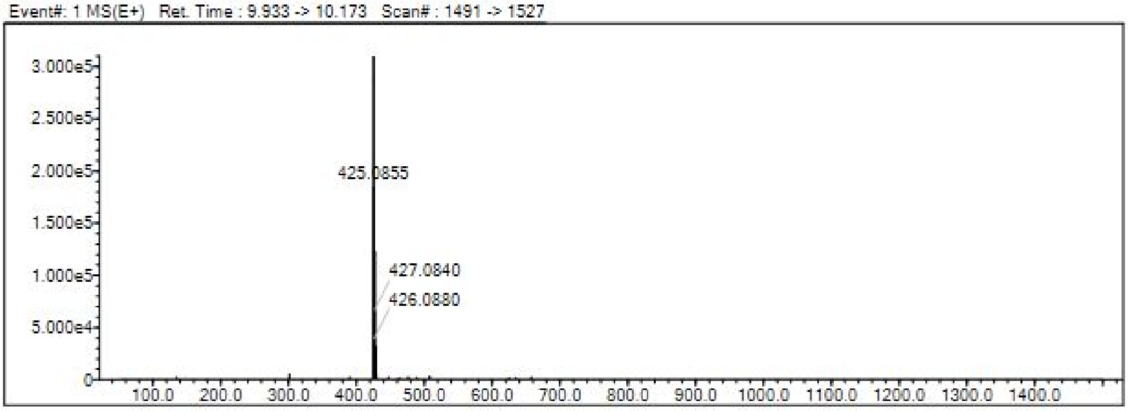

Measured region for 425.0855 m/z

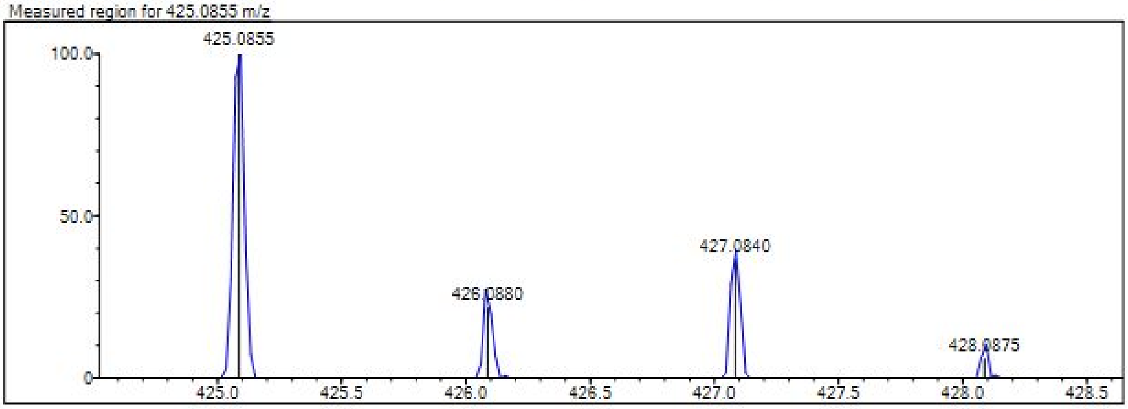

C21 H17 N4 O2 S Cl [M+H]+ : Predicted region for 425.0834 m/z

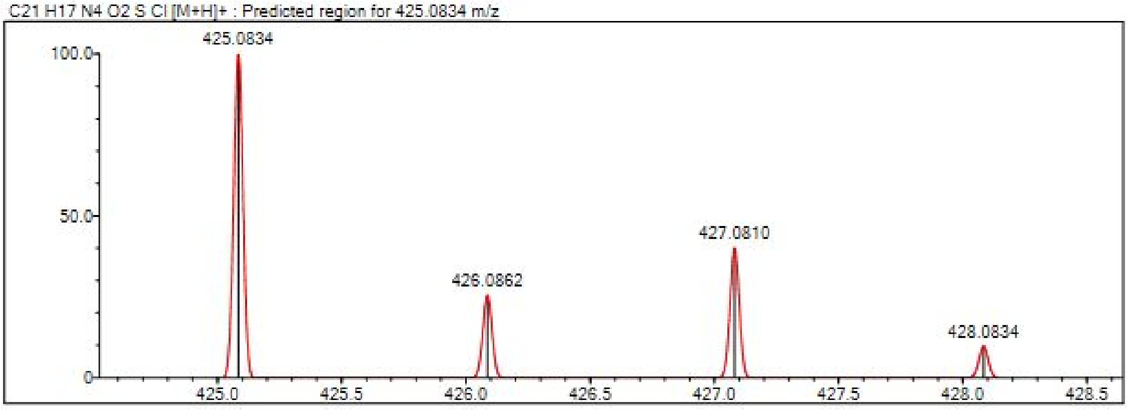

Rank	Score	Formula (M)	Ion	Meas. m/z	Pred. m/z	Df. (mDa)	Df. (ppm)	Iso	DBE
1	74.06	C21 H17 N4 O2 S Cl	[M+H]+	425.0855	425.0834	2.1	4.94	82.15	15.0

Figure S33. *Compound 4k HRMS spectrum*

1.12. 2-{2-[(2-Chloro-6-methoxyquinolin-3-yl)methylene]hydrazinyl}-4-(pyridin-4-yl)thiazole (4l)

M.p: 250-257 °C. Appearance: Dark yellow powder. Yield: %86.

^{1}H-NMR (300 MHz, DMSO-*d6*): δ: 3.92 (3H, s, O-CH_3), 7.45 (H, dd, J_1=2.80 Hz, J_2=9.12 Hz, quinoline H_7), 7.61 (H, d, *J*=2.77 Hz, quinoline H_5), 7.83-7.88 (4H, m, quinoline H_8, thiazole H_5, pyridine H_2, H_6), 8.44 (H, s, -N=C-H), 8.64 (2H, d, *J*=6.00 Hz, pyridine H_3, H_5), 8.72 (H, s, quinoline H_4), 12.73 (H, brs, =N-N-H).

^{13}C-NMR (75 MHz, DMSO-*d6*): δ: 56.23 (C, s, O-CH_3), 106.82 (C, s, quinoline C_5), 110.23 (C, s, thiazole C_5), 120.55 (C, s, pyridine $C_{2,6}$), 124.37 (C, s, quinoline C_7), 126.58 (C, s, quinoline C_3), 128.69 (C, s, quinoline C_{4a}), 129.52 (C, s, quinoline C_8), 133.57 (C, s, pyridine C_1), 137.12 (C, s, quinoline C_4), 142.47 (C, s, thiazole C_4), 143.31 (C, s, N=C), 145.83 (C, s, quinoline C_{8a}), 148.49 (C, s, quinoline C_2), 149.71 (C, s, pyridine $C_{3,5}$), 158.53 (C, s, quinoline C_6), 168.62 (C, s, thiazole C_2).

HRMS (-m/z): $[M+H]^+$: For $C_{19}H_{14}N_5OSCl$ calculated: 396.0680, found: 396.0668

InChI=1S/C19H14ClN5OS/c1-26-15-2-3-16-13(9-15)8-14(18(20)23-16)10-22-25-19-24-17(11-27-19)12-4-6-21-7-5-12/h2-11H,1H3,(H,24,25)/b22-10+

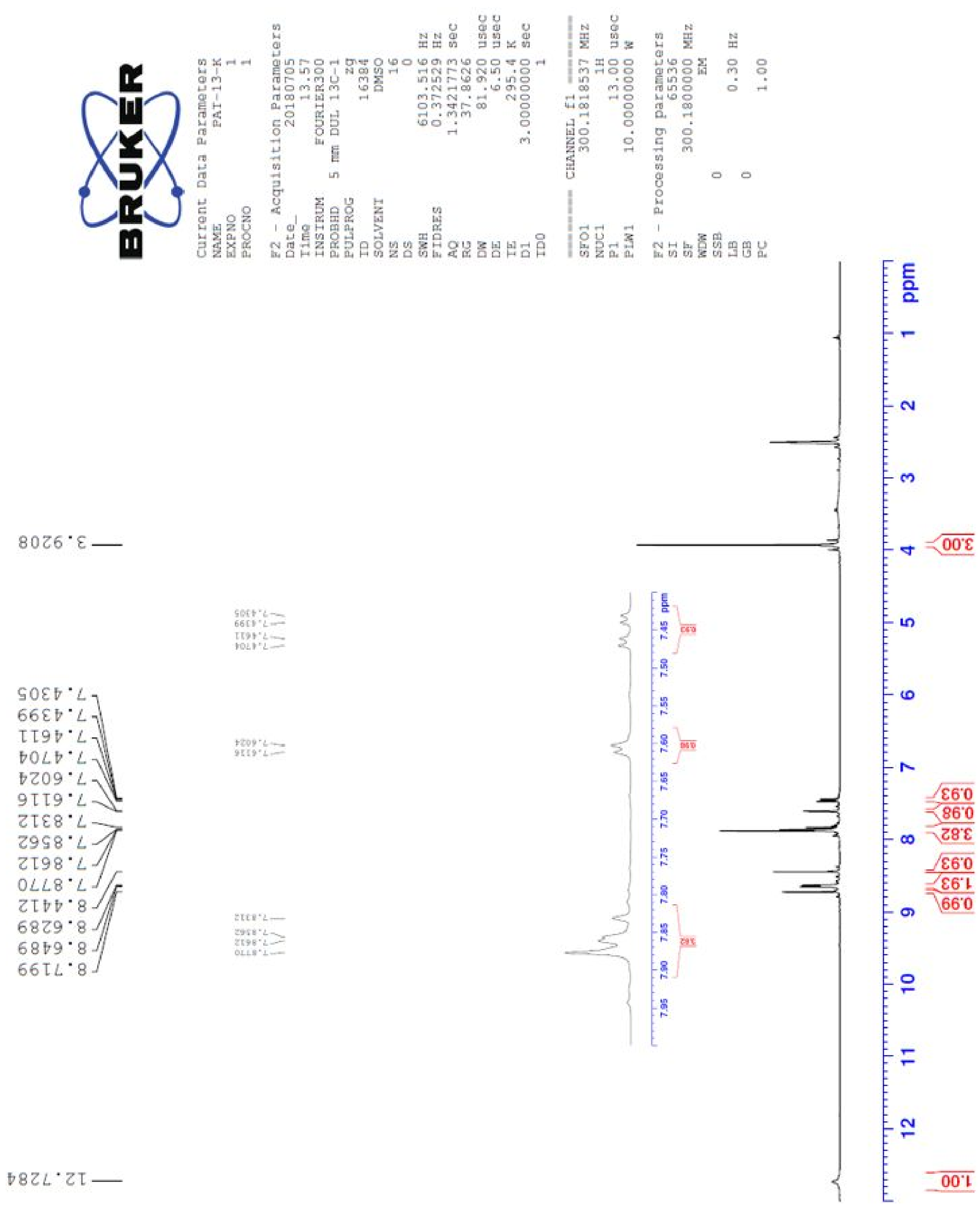

Figure S34. *Compound 4l* [1]*HNMR spectrum*

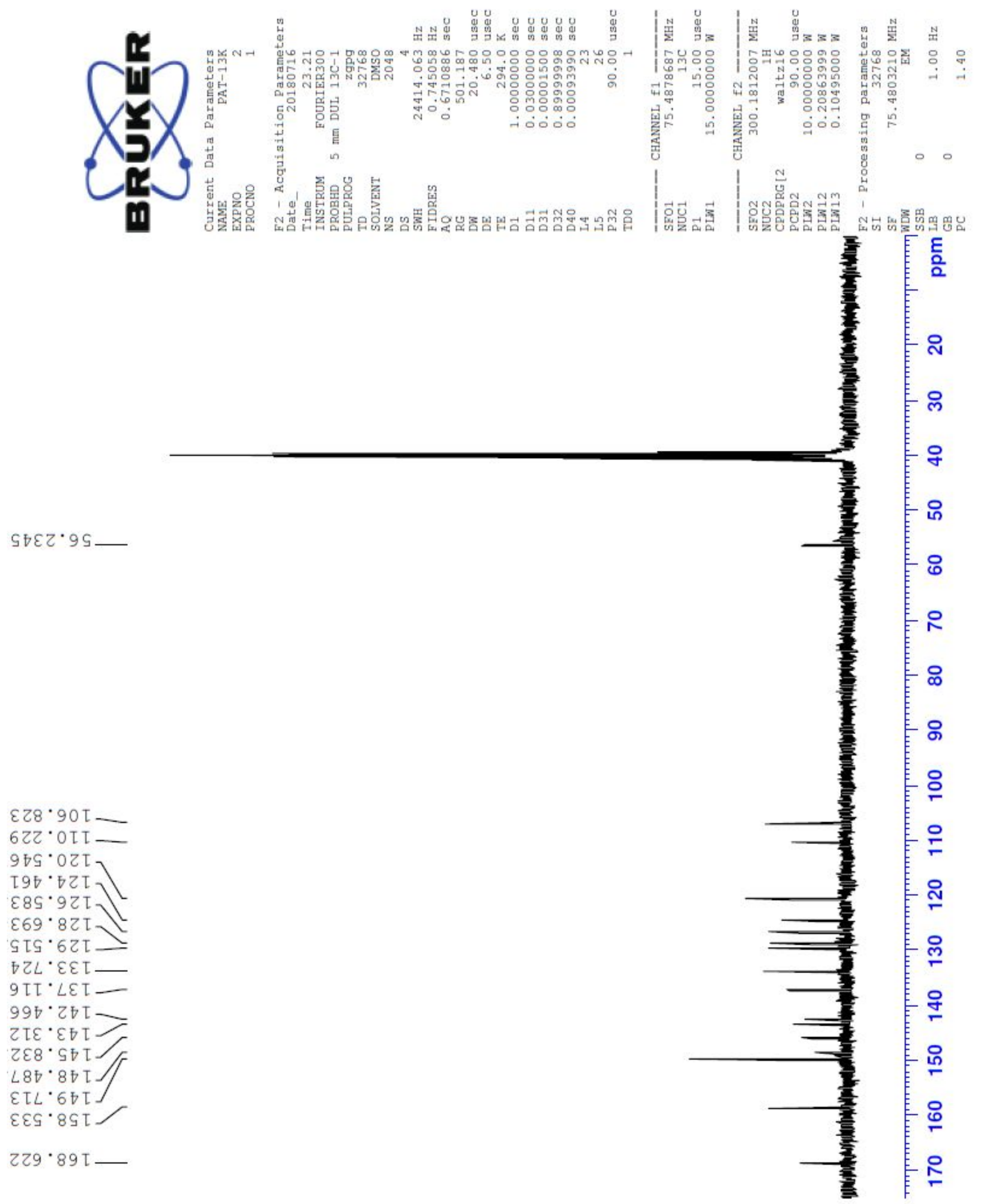

Figure S35. *Compound 4l* ^{13}*CNMR spectrum*

Formula Predictor Report - PAT-13A_1.lcd

Data File: C:\LabSolutions\Data\Analiz\Lyuttas\PAT-13A_1.lcd

Elmt	Val.	Min	Max	Elmt	Val.	Min	Max	Elmt	Val.	Min	Max	Elmt	Val.	Min	Max	Use Adduct
H	1	0	31	O	2	1	3	S	2	0	1	Ru	2	0	0	H
C	4	0	25	F	1	0	0	Cl	1	1	1	Pd	2	0	0	
N	3	0	5	P	3	0	0	Br	1	0	0	I	3	0	0	

Error Margin (ppm): 10
HC Ratio: unlimited
Max Isotopes: 3
MSn Iso RI (%): 10.00

DBE Range: 2.0 - 16.0
Apply N Rule: yes
Isotope RI (%): 1.00
MSn Logic Mode: AND

Electron Ions: both
Use MSn Info: yes
Isotope Res: 9000
Max Results: 500

Event#: 1 MS(E+) Ret. Time : 8.227 Scan# : 1235

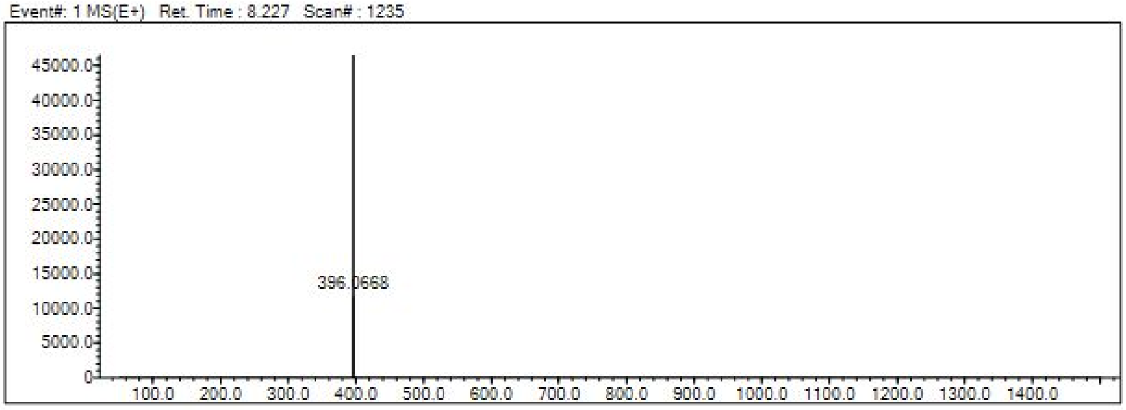

Measured region for 396.0668 m/z

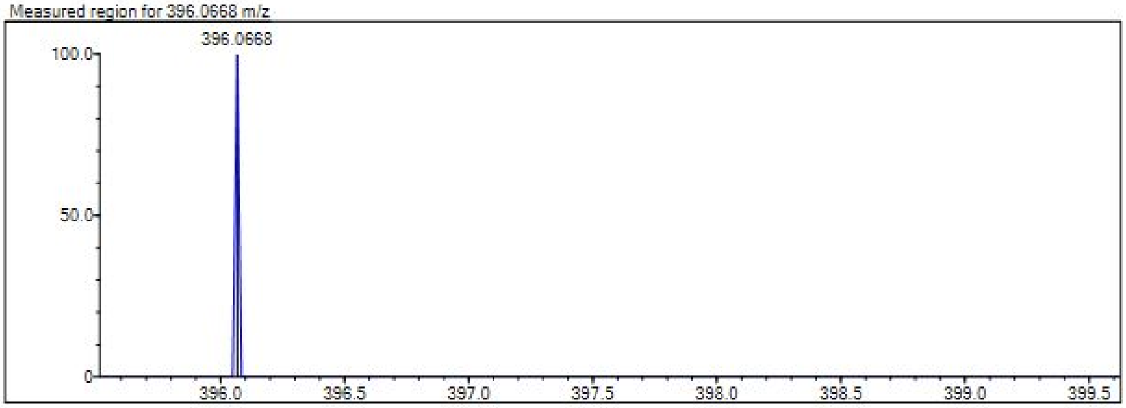

C19 H14 N5 O S Cl [M+H]+ : Predicted region for 396.0680 m/z

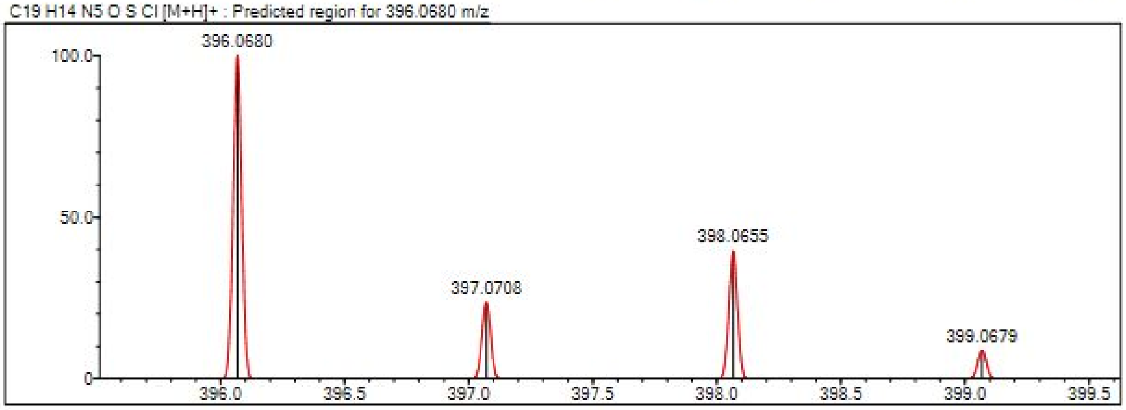

Rank	Score	Formula (M)	Ion	Meas. m/z	Pred. m/z	Df. (mDa)	Df. (ppm)	Iso	DBE
1	0.00	C19 H14 N5 O S Cl	[M+H]+	396.0668	396.0680	-1.2	-3.03	0.00	15.0

Figure S36. *Compound 4l HRMS spectrum*

1.13. 2-{2-[(2-Chloro-6-methoxyquinolin-3-yl)methylene]hydrazinyl}-4-(naphthalen-1-yl)thiazole (4m)

M.p: 174-179 °C. Appearance: Dark yellow powder. Yield: %77.

^{1}H-NMR (300 MHz, DMSO-*d6*): δ: 3.92 (3H, s, O-CH_3), 7.21 (H, s, thiazole H_5), 7.45 (H, dd, J_1=2.80 Hz, J_2=9.18 Hz, quinoline H_7), 7.53-7.58 (3H, m, naphthalene H_5, H_6, H_7), 7.61 (H, d, J= 2.79 Hz, quinoline H_5), 7.72 (H, dd, J_1= 1.12 Hz, J_2= 7.14 Hz, naphthalene H_2), 7.85 (H, d, J=9.24 Hz, quinoline 8), 7.93-7.99 (2H, m, naphthalene H_3, H_4), 8.43-8.47 (2H, m, -N=C-H, naphthalene H_8), 8.73 (H, s, quinoline H_4), 12.66 (H, brs, =N-N-H).

^{13}C-NMR (75 MHz, DMSO-*d6*): δ: 56.23 (C, s, O-CH_3), 106.78 (C, s, quinoline C_5), 108.38 (C, s, thiazole C_5), 124.36 (C, s, quinoline C_7), 125.94 (C, s, naphthalene C_2), 126.40 (C, s, naphthalene $C_{6\ or}$ naphthalene C_7), 126.46 (C, s, naphthalene $C_{6\ or}$ naphthalene C_7), 126.61 (C, s, quinoline C_3), 126.78 (C, s, naphthalene C_3), 127.40 (C, s, quinoline C_{4a}), 128.72 (C, s, naphthalene $C_{4\ or}$ naphthalene C_5), 128.81 (C, s, naphthalene $C_{4\ or}$ naphthalene C_5), 129.51 (C, s, quinoline C_8), 131.09 (C, s, naphthalene C_8), 133.40 (C, s, naphthalene C_{8a}), 133.52 (C, s, naphthalene C_{4a}), 133.97 (C, s, naphthalene C_1), 136.54 (C, s, quinoline C_4), 143.25 (C, s, N=C), 145.84 (2C, s, quinoline C_{8a}), 151.15 (C, s, quinoline C_2), 158.55 (C, s, quinoline C_6, thiazole C_4), 167.84 (C, s, thiazole C_2).

HRMS (-m/z): $[M+H]^+$: For $C_{24}H_{17}N_4OSCl$ calculated: 445.0884, found: 445.0891

InChI=1S/C24H17ClN4OS/c1-30-18-9-10-21-16(12-18)11-17(23(25)27-21)13-26-29-24-28-22(14-31-24)20-8-4-6-15-5-2-3-7-19(15)20/h2-14H,1H3,(H,28,29)/b26-13+

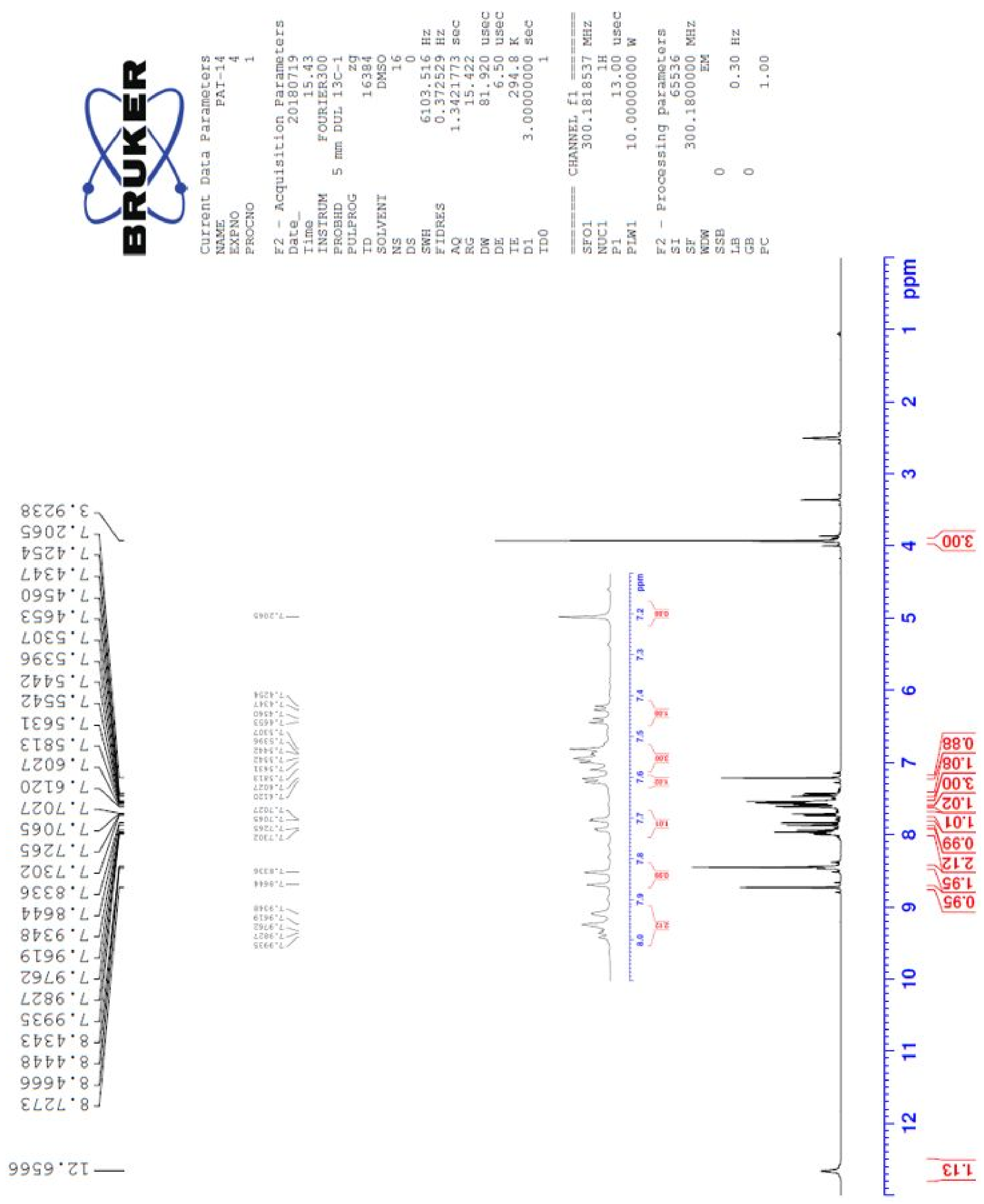

Figure S37. *Compound 4m ^{1}HNMR spectrum*

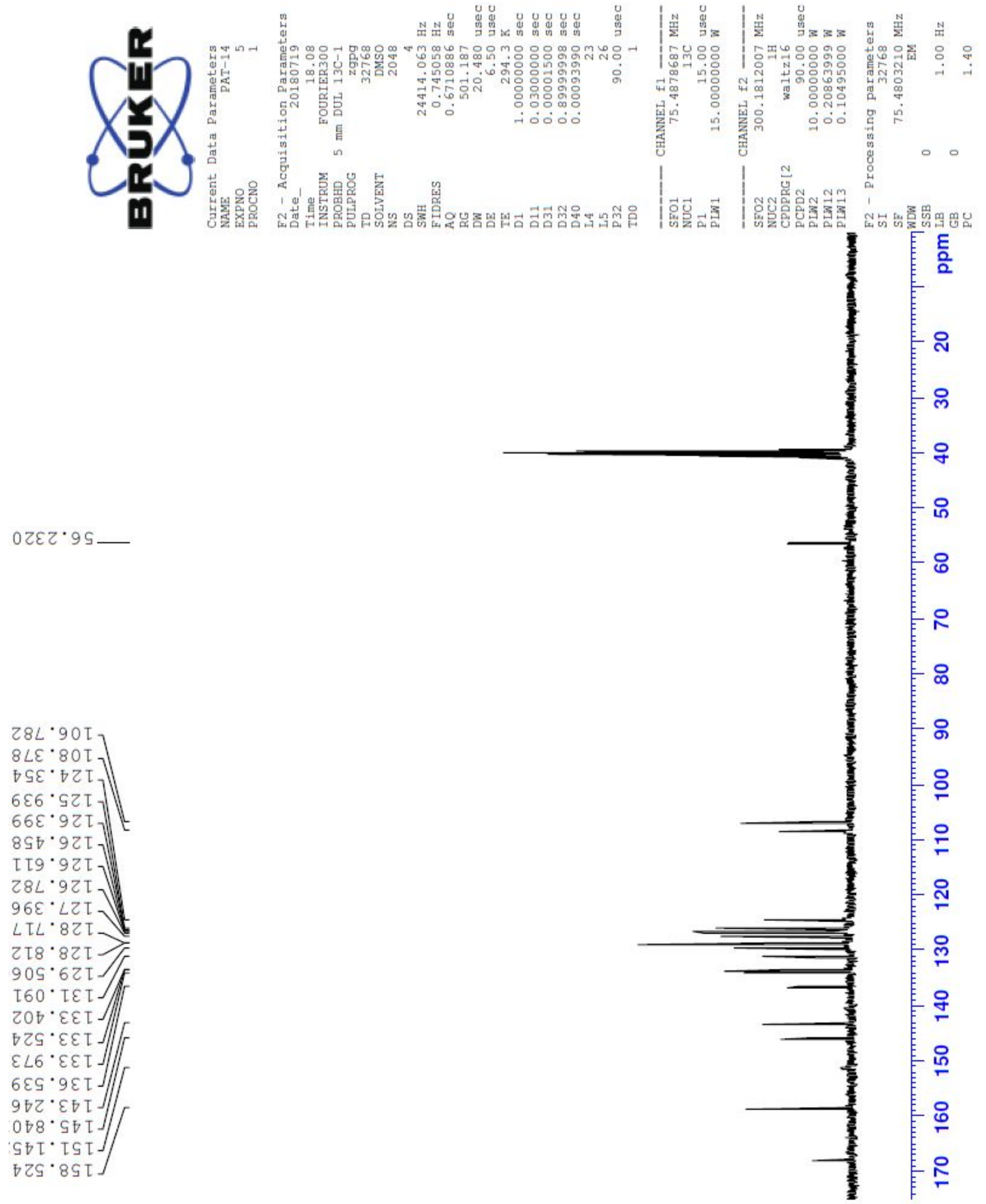

Figure S38. *Compound 4m* ^{13}*CNMR spectrum*

Data File: C:\LabSolutions\Data\Analiz\Lyuttas\ACN_32.lcd

Elmt	Val.	Min	Max	Elmt	Val.	Min	Max	Elmt	Val.	Min	Max	Elmt	Val.	Min	Max	Use Adduct
H	1	0	31	O	2	1	2	S	2	0	1	Ru	2	0	0	H
C	4	0	25	F	1	0	0	Cl	1	1	2	Pd	2	0	0	
N	3	4	5	P	3	0	0	Br	1	0	0	I	3	0	0	

Error Margin (ppm):	10	DBE Range:	8.0 - 20.0	Electron Ions:	both
HC Ratio:	unlimited	Apply N Rule:	yes	Use MSn Info:	yes
Max Isotopes:	3	Isotope RI (%):	1.00	Isotope Res:	9000
MSn Iso RI (%):	10.00	MSn Logic Mode:	AND	Max Results:	500

Event#: 1 MS(E+) Ret. Time : 9.493 -> 9.733 Scan# : 1425 -> 1461

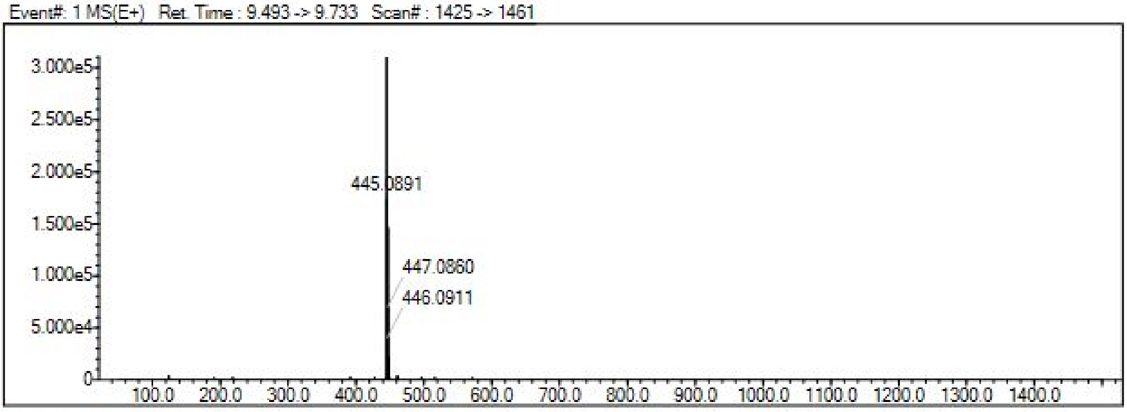

Measured region for 445.0891 m/z

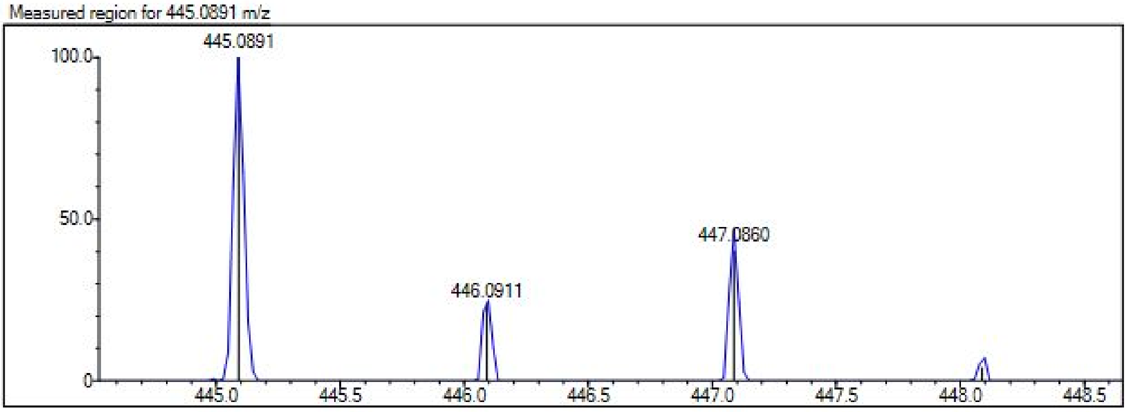

C24 H17 N4 O S Cl [M+H]+ : Predicted region for 445.0884 m/z

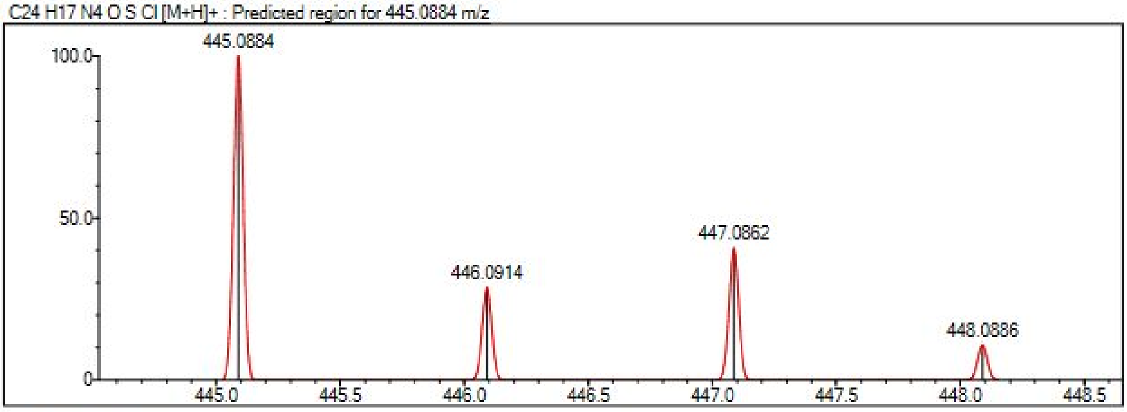

Rank	Score	Formula (M)	Ion	Meas. m/z	Pred. m/z	Df. (mDa)	Df. (ppm)	Iso	DBE
1	75.19	C24 H17 N4 O S Cl	[M+H]+	445.0891	445.0884	0.7	1.57	76.28	18.0

Figure S39. *Compound 4m HRMS spectrum*

1.14. 2-{2-[(2-Chloro-6-methoxyquinolin-3-yl)methylene]hydrazinyl}-4-(naphthalen-2-yl)thiazole (4n)

M.p: 199-202 °C. Appearance: light yellow powder. Yield: %76.

^{1}H-NMR (300 MHz, DMSO-*d6*): δ: 3.93 (3H, s, O-CH_3), 7.45 (H, dd, J_1= 2.80 Hz, J_2= 9.21 Hz, quinoline H_7), 7.50-7.54 (2H, m, naphthalene H_6, H_7), 7.59 (H, s, thiazole H_5), 7.62 (H, d, J= 2.76 Hz, quinoline H_5), 7.86 (H, d, J= 9.18 Hz, quinoline H_8), 7.90-7.98 (3H, m, naphthalene H_2, H_3, H_5), 8.03 (H, dd, J_1=1.52 Hz, J_2= 8.62 Hz, naphthalene H_4), 8.40 (H, s, naphthalene H_8), 8.45 (H, s, -N=C-H), 8.74 (H, s, quinoline H_4), 12.70 (H, brs, =N-N-H).

^{13}C-NMR (75 MHz, DMSO-*d6*): δ: 56.24 (C, s, O-CH_3), 105.63 (C, s, thiazole C_5), 106.82 (C, s, quinoline C_5), 124.37 (C, s, quinoline C_7), 124.61 (C, s, naphthalene C_{8a}), 126.53 (C, s, naphthalene C_6 or naphthalene C_7), 126.73 (C, s, quinoline C_3), 126.94 (C, s, naphthalene C_6 or naphthalene C_7), 128.06 (C, s, quinoline C_{4a}), 128.66 (2C, s, naphthalene C_5, C_8), 128.73 (C, s, quinoline C_8), 129.52 (C, s, naphthalene C_3), 132.42 (2C, s, naphthalene C_1,C_4), 132.94 (C, s, naphthalene C_2), 133.61 (C, s, naphthalene C_{4a}), 136.54 (C, s, quinoline C_4), 143.27 (C, s, N=C), 145.84 (C, s, quinoline C_{8a}), 151.11 (C, s, quinoline C_2), 158.53 (2C, s, quinoline C_6, thiazole C_4), 168.19 (C, s, thiazole C_2).

HRMS (-m/z): [M+H]$^+$: For C$_{24}H_{17}N_4OSCl$ calculated: 445.0884, found: 445.0885

InChI=1S/C24H17ClN4OS/c1-30-20-8-9-21-18(12-20)11-19(23(25)27-21)13-26-29-24-28-22(14-31-24)17-7-6-15-4-2-3-5-16(15)10-17/h2-14H,1H3,(H,28,29)/b26-13+

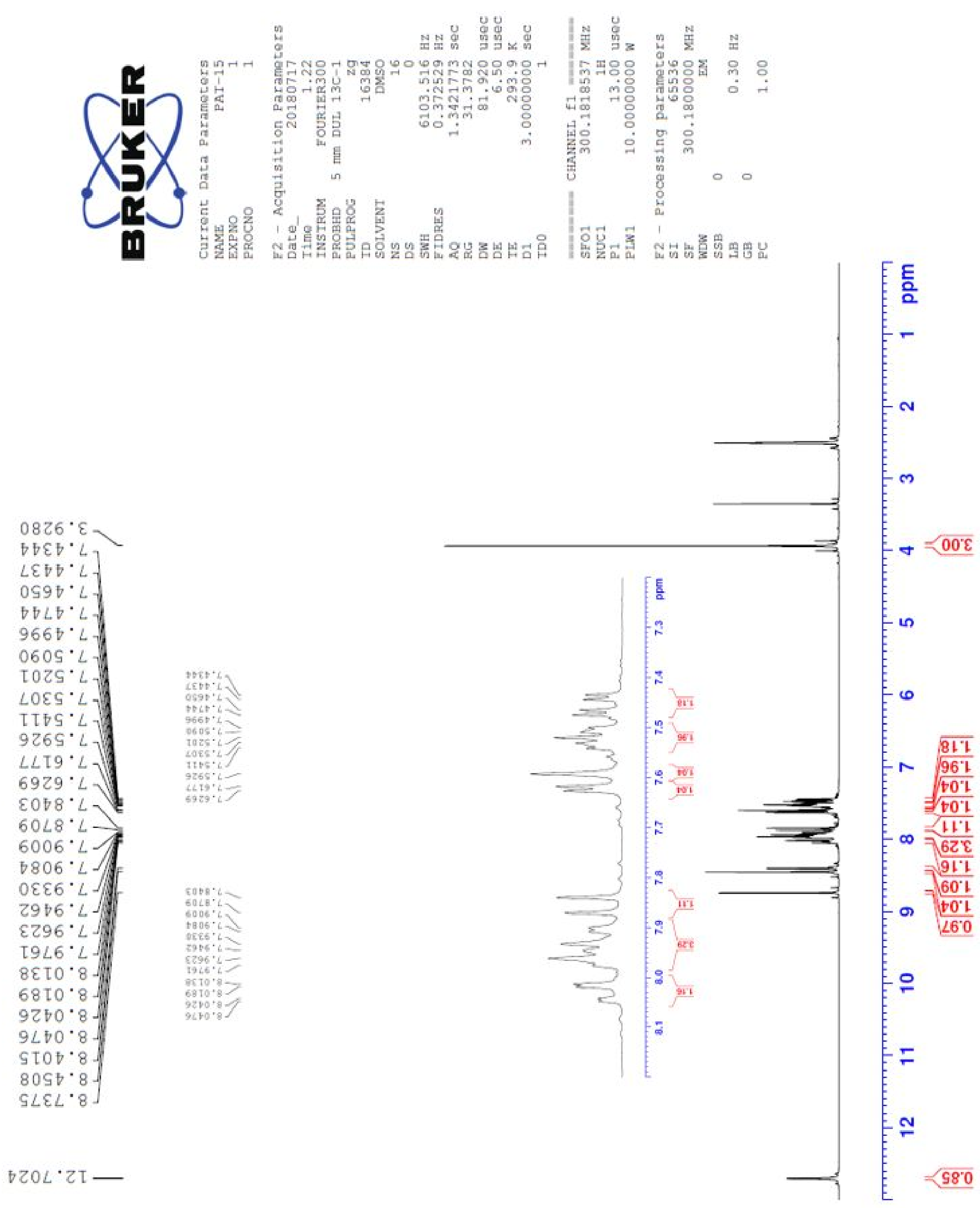

Figure S40. *Compound 4n* [1]*HNMR spectrum*

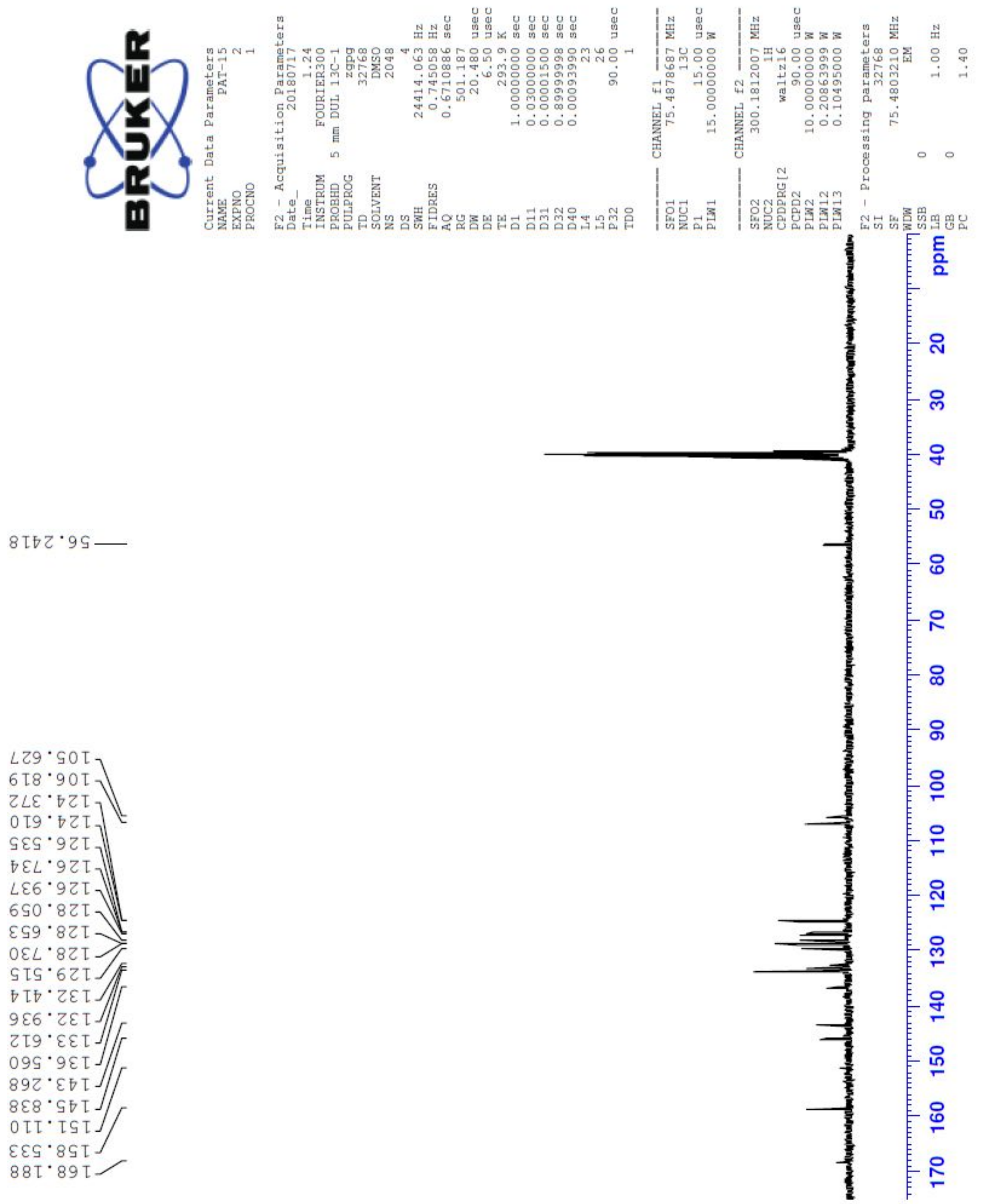

Figure S41. *Compound 4n ^{13}CNMR spectrum*

Data File: C:\LabSolutions\Data\Analiz\Lyuttas\PAT-15_29.lcd

Elmt	Val.	Min	Max	Elmt	Val.	Min	Max	Elmt	Val.	Min	Max	Elmt	Val.	Min	Max	Use Adduct
H	1	0	31	O	2	1	2	S	2	0	1	Ru	2	0	0	H
C	4	0	25	F	1	0	0	Cl	1	1	2	Pd	2	0	0	
N	3	4	5	P	3	0	0	Br	1	0	0	I	3	0	0	

Error Margin (ppm): 10
HC Ratio: unlimited
Max Isotopes: 3
MSn Iso RI (%): 10.00

DBE Range: 8.0 - 20.0
Apply N Rule: yes
Isotope RI (%): 1.00
MSn Logic Mode: AND

Electron Ions: both
Use MSn Info: yes
Isotope Res: 9000
Max Results: 500

Event#: 1 MS(E+) Ret. Time : 8.720 -> 8.960 Scan# : 1309 -> 1345

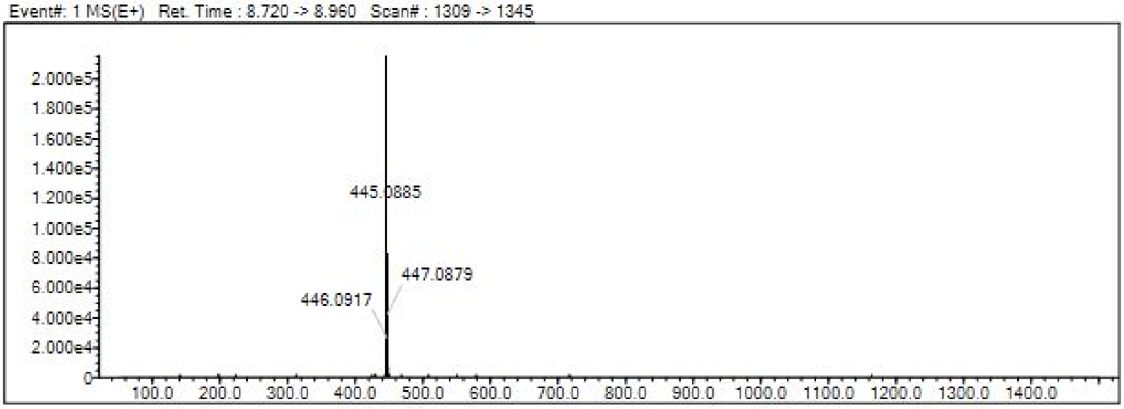

Measured region for 445.0885 m/z

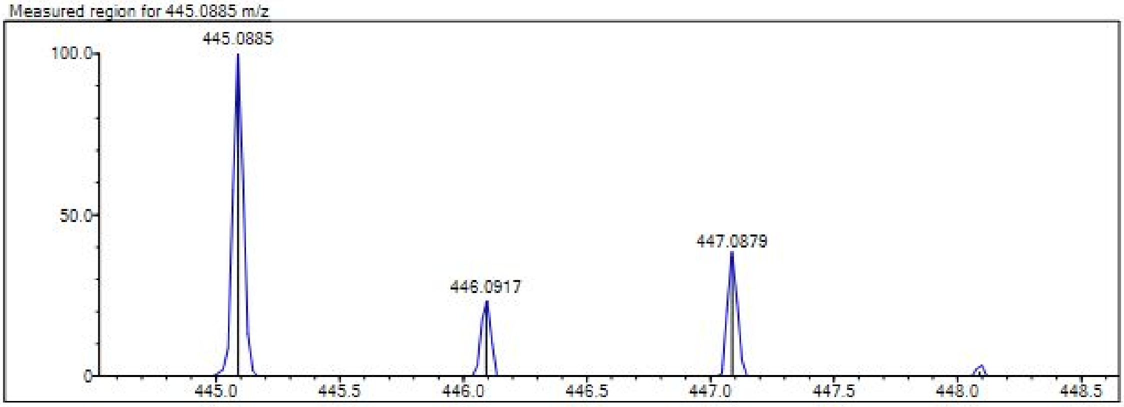

C24 H17 N4 O S Cl [M+H]+ : Predicted region for 445.0884 m/z

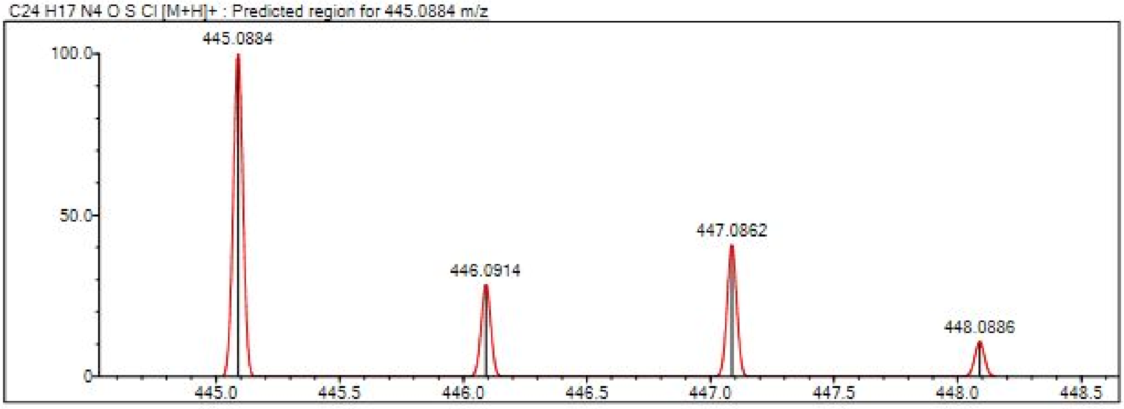

Rank	Score	Formula (M)	Ion	Meas. m/z	Pred. m/z	Df. (mDa)	Df. (ppm)	Iso	DBE
1	67.62	C24 H17 N4 O S Cl	[M+H]+	445.0885	445.0884	0.1	0.22	67.62	18.0

Figure S42. *Compound 4n HRMS spectrum*
